# Functionalized Cyclopentenes via the Formal [4+1] Cycloaddition of Photogenerated Siloxycarbenes from Acyl Silanes

**DOI:** 10.1021/acs.joc.2c00591

**Published:** 2022-06-23

**Authors:** João R. Vale, Rafael F. Gomes, Carlos A. M. Afonso, Nuno R. Candeias

**Affiliations:** † iMed.ULisboa, Faculty of Pharmacy, Universidade de Lisboa, Av. Prof. Gama Pinto, Lisbon 1649-003, Portugal; ‡ Faculty of Engineering and Natural Sciences, Tampere University, Korkeakoulunkatu 8, Tampere 33101, Finland; § LAQV-REQUIMTE, Department of Chemistry, University of Aveiro, Aveiro 3810-193, Portugal

## Abstract

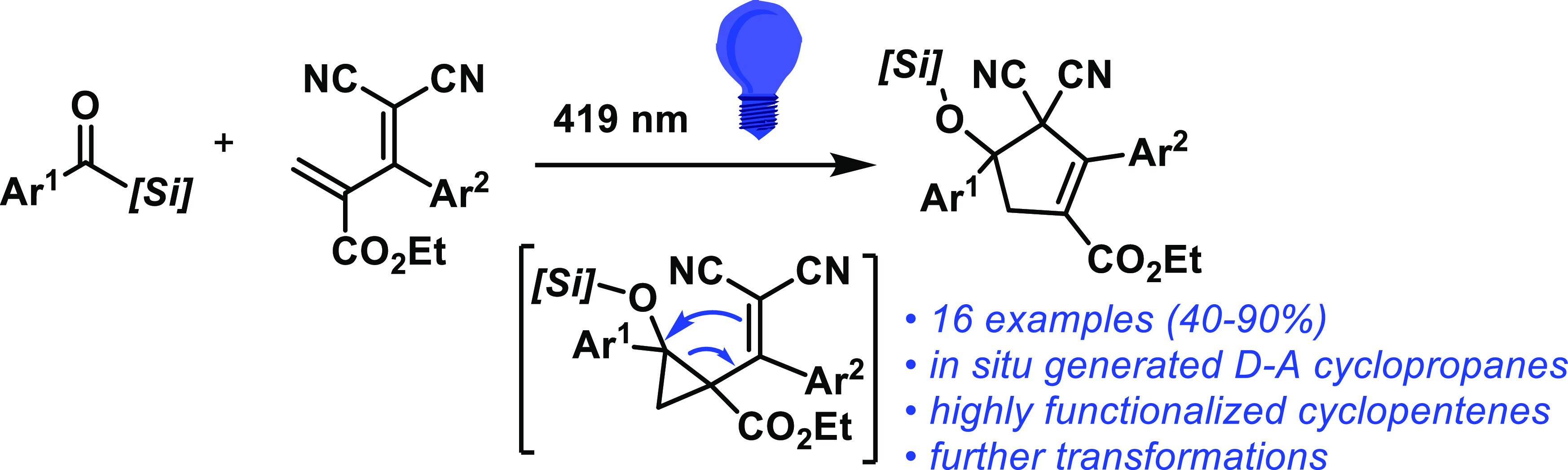

This work describes the first formal cycloaddition reaction of photogenerated nucleophilic carbenes derived from acylsilanes with electrophilic dienes. The resulting transient donor–acceptor cyclopropane rearranges to its stable and highly functionalized cyclopentene isomer in an unprecedented metal-free process. The cyclopropanation–vinyl cyclopropane rearrangement sequence was corroborated by computational calculations. The cyclopropane formation corresponds to a higher energetic barrier, and the vinylcyclopropane–cyclopentene rearrangement proceeds through different mechanisms, although of comparable energies, depending on the stereochemistry of the cyclopropane.

## Introduction

Acylsilanes have recently attracted considerable attention in the field of organic synthesis mainly due to their ability to generate nucleophilic carbenes.^1−3^ The metal-free UV–visible irradiation of acylsilanes presents an atom-efficient way to deliver siloxycarbenes upon a reversible 1,2-Brook rearrangement. Despite their clean production, these carbenes have seen limited applications due to their low reactivity and fast reversibility to the acylsilane precursor that can also undergo a homolytic cleavage.^4^ The siloxycarbenes generated by the irradiation of acylsilanes can undergo a plethora of X–H insertions, such as O–H,^5^ N–H,^6^ S–H,^4^ Si–H,^7,8^ and B–H.^9^ Although limited to its intramolecular version, C–H insertion of siloxycarbenes has been explored in the preparation of benzofurans,^10^ while thermolytic methods proved synthetically nonuseful.^11,12^ B–C insertion was also observed with organoboronic esters allowing an elegant photochemical transition metal-free cross-coupling.^13^ The repertoire of reactions of siloxycarbenes also encompasses the nucleophilic addition to aldehydes,^14,50^ trifluoromethyl ketones,^15^ and carbon dioxide.^16^

**Scheme 1:**
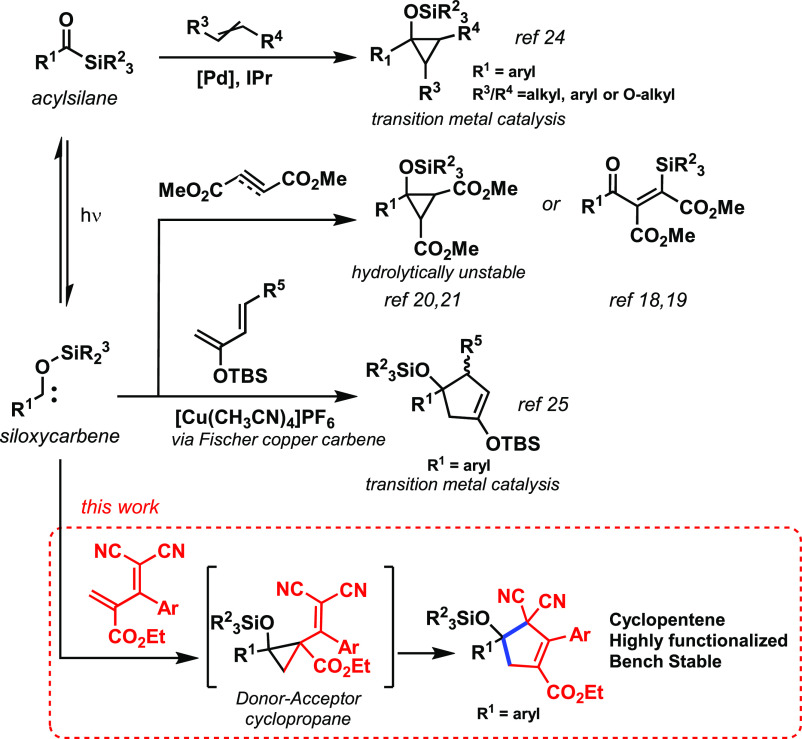
Cycloaddition Reactions of Siloxycarbenes

Cyclopropanation, a classical carbene reaction,^17^ is elusive due to the nucleophilic nature of the siloxycarbene. Activated alkynes have been shown to undergo slow inter- and intramolecular cyclopropenations following ring collapse to give β-silylated enones (Scheme 1),^18,19^ while ambiphilic donor–acceptor carbenes derived from trifluoroacetylsilanes were recently explored.^53,54^ Concerning olefins, only the highly electron-withdrawing dialkyl fumarate and maleate have been reported to undergo cyclopropanation,^20,21^ yielding cyclopropyl silyl ethers that are prone to ring opening through hydrolysis and are therefore not synthetically useful for cyclopropane synthesis. Despite the preference for acylsilane carbonyl to react in a [2+2]-photocycloaddition reaction,^22,23^ singlet nucleophilic carbenes with tethered olefins were recently shown to undergo rapid [2+1]-cycloaddition.^55^ All in all, formal cyclopropanation of olefins with acylsilane-derived carbenes remains poorly explored due to the required presence of electron-withdrawing groups in the olefin which renders instability to the resulting silyl ether cyclopropanes. Recently, further advances on the topic rely heavily on transition metal catalysis. Cyclopropanation of nonactivated olefins using acylsilanes was achieved through palladium catalysis via a Fischer-type carbene complex without the involvement of a photogenerated siloxycarbene (Scheme 1, top).^24^

The formation of cyclopentenes through the intermediacy of siloxycarbenes was recently achieved via the umpolung strategy (Scheme 1).^25^ The photogenerated carbene complexes with copper to form an electrophilic Fischer-type carbene that reacts with highly electron-rich dienes in (4 + 1) cycloaddition, thus circumventing the electronic mismatch between the traditional nucleophilic siloxycarbene and electron-rich cycloaddition partner. Such findings led us to hypothesize that a metal-free strategy using acylsilanes to synthesize cyclopentenes could be achieved by tuning the electronic nature of the diene. We envisioned that the reaction of an acylsilane-derived carbene with an electron-withdrawing diene would lead to a donor–acceptor (D–A) cyclopropane^26−29^ that would be a prone candidate for a vinyl cyclopropane rearrangement,^30−32^ ultimately forming a stable cyclopentene (Scheme 1, bottom). Notwithstanding the reactivity of D–A cyclopropanes, the vinylcyclopropane–cyclopentene rearrangement usually requires the presence of Lewis acids of variable strengths^33−36^ depending on the electronic nature of the cyclopropane substituents.

Dicyano-2-methylenebut-3-enoates have been previously used as electron-withdrawing dienes for the inverse-electron-demand Diels–Alder reactions,^37^ and their terminal olefin was observed to undergo cyclopropanation with diazo compounds.^38^ Hence, this highly electrophilic diene was considered a suitable candidate for the trapping of siloxycarbenes derived from benzoyl silanes.

## Results and Discussion

The studies were initiated using *p*-toluoyltrimethylsilane **1a** as a carbene precursor and diene **2a** in slight excess. Prolonged irradiation at 419 nm of a hexane/DCM solution gladly resulted in the domino production of cyclopentene **3** in a 20% yield (Table 1, entry 1), despite the absence of any Lewis acid catalyst. Additional measures taken to remove any traces of moisture, that is, using dry solvents and molecular sieves, proved futile, and *p*-tolualdehyde was the main side product.

**Table 1:**
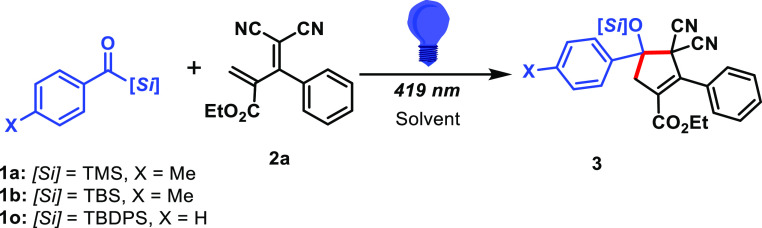
Optimization of the Cyclopropanation–Vinyl Cyclopropane Rearrangement Sequence

entry^a^	solvent	time (h)	1/[1] (mM)	diene **2a** (equiv)	**3** yield (%)
1	DCM/Hex	12	**1a**/20	1.2	20
2	toluene	12	**1a**/20	1.2	30
3	toluene	55	**1a**/20	2.8	65
4	toluene	55	**1a**/40	2.8	trace
5	toluene	23	**1a**/10	2.8	70
6	toluene	23	**1a**/10	1.4	66
7	toluene	55	**1b**/10	1.4	70
8	toluene	55	**1b**/20	1.4	74
9	toluene	48	**1o**/10	1.4	0

a General procedure: Acylsilane **1** (0.1 mmol), diene, and molecular sieves 4 Å (200 mg/mL of solvent) are dissolved in a solvent in a sealed Pasteur pipette. Solution was purged with argon for 15 min and irradiated at 419 nm until the reaction progress halted.

**Scheme 2:**
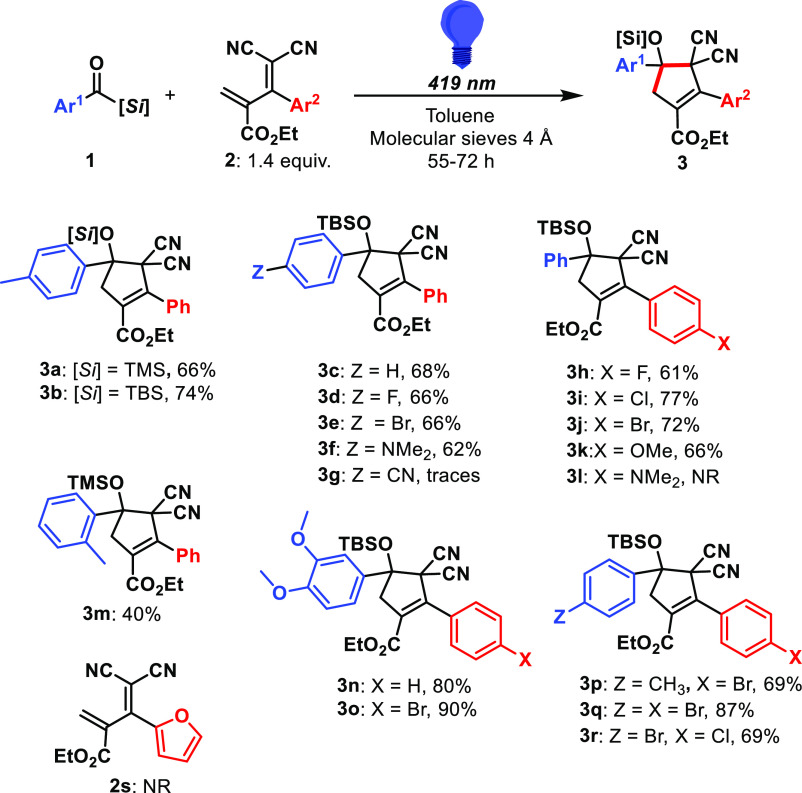
Scope of the Cyclopropanation–Vinyl Cyclopropane Rearrangement Sequence

Notably, no cyclopropane intermediate was isolated nor detected in the crude reaction mixture, indicating that the vinyl cyclopropane rearrangement is a highly favored process not requiring high temperatures or a catalyst. The use of dry toluene as a solvent and the addition of molecular sieves suppressed the aldehyde formation, and the increase in the amount of the diene to 2.8 equiv led to a cyclopentene **3** yield increasing to 70% (Table 1, entry 5). The amount of diene could be reduced by 1.4 equiv without significantly compromising the yield (Table 1, entry 6). As small amounts of the diallylated product **4** (Scheme 4) were detected in every experiment with **1a**, and suspecting the lability of the trimethylsilyl (TMS) group, benzoyl silane was decorated with a bulkier tert-butyldimethylsilyl (TBS). Despite the longer reaction times required to reach full conversion, the desilylated product was not detected, and **3b** was obtained in a slightly improved 74% yield (Table 1, entry 7). The cyclopentene structure was confirmed through X-ray diffraction analysis of product **3b** (see the Supporting Information). Irradiation of even bulkier benzoyl *t*-butyldiphenylsilane **1o** (Table 1, entry 9) led to no conversion and full recovery of the starting material. With the optimal reaction conditions cleared, we investigated the scope of the reaction by changing the aryl groups of the benzoyl silane and diene (Scheme 2). Para substitution within the aromatic ring of the benzoyl silane was well tolerated, allowing electron-donating (**3f**, **3n** and **3o**) and slightly electron-withdrawing substituents (**3d**, **3e**, **3q**, and **3r**). Meta- (**3n** and **3o**) and ortho- (**3m**) substitutions were also tolerated. Only the highly electron-withdrawing nitrile was unreactive toward the formation of cyclopentene **3g**, presumably due to the lower nucleophilicity of the generated carbene. Substitution on the aromatic ring of the diene was more challenging. Moderate electron-withdrawing groups such as halogens (**3h–3j** and **3p-r**) as well as electron-donating groups such as methoxy (**3k**) were compatible. However, highly electron-donating groups such as dimethylamine (**2l**) led to no reactivity, a result of its higher lowest unoccupied molecular orbital energy. Furan derivative **2s** was surprisingly unreactive. Nitro and nitrile derivatives would likely present suitable reactivity, as highly electron-withdrawing dienes, but their synthesis proved impossible with the used protocol.^37^

**Figure 1:**
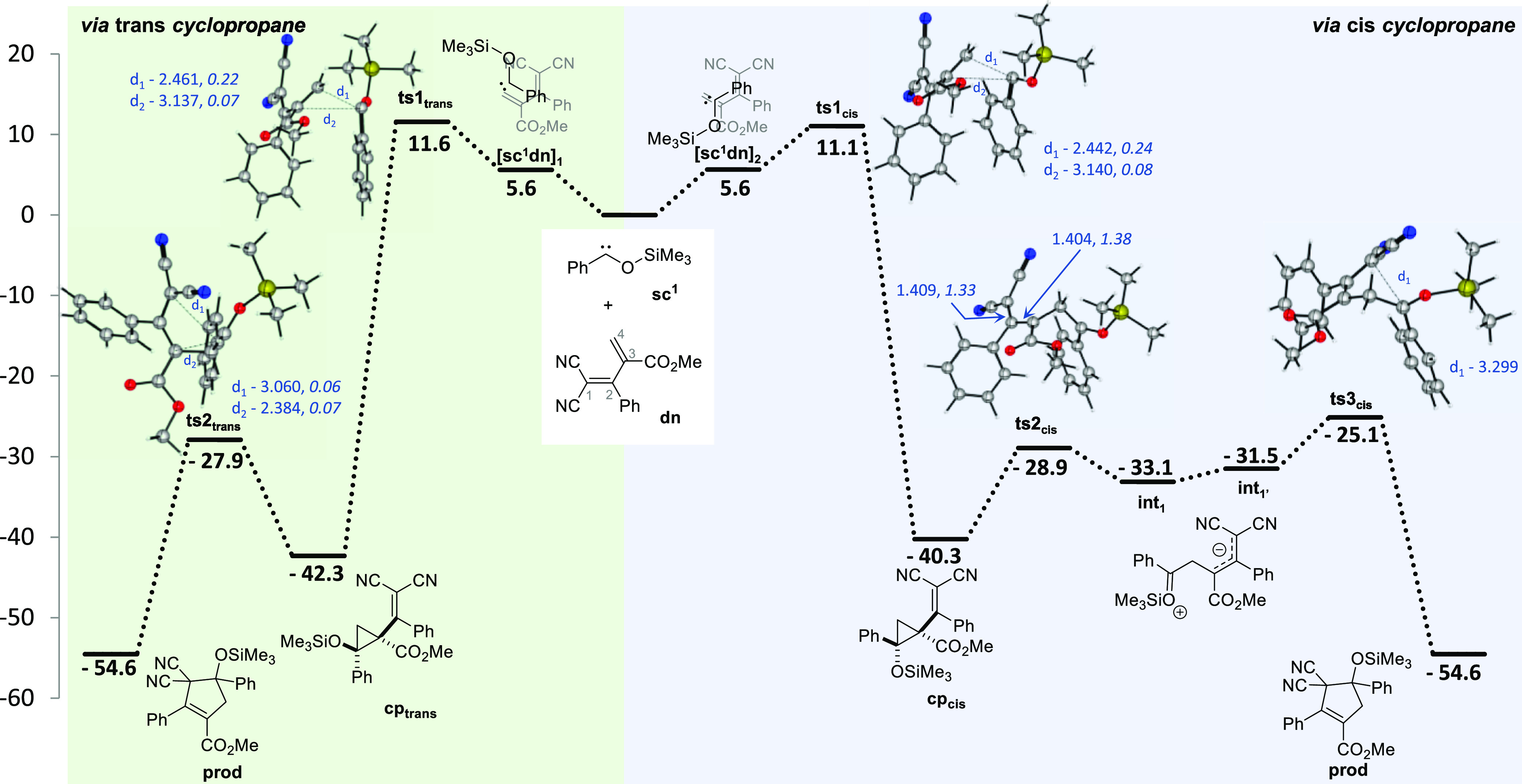
Free energy profile (M06-2X/6-311++G**//M06-2X/6-31+G**) and a mechanistic representation for the cyclopropanation rearrangement to cyclopentene of separated model substrates siloxycarbene **sc^1^** and diene **dn**. The geometries of the minima and the transition states were optimized, and the energy values (kcal/mol) refer to the optimized **sc^1^** and **dn** and include the thermal correction to Gibb’s free energy in toluene. Bond lengths (in Å) and Wiberg indices (WIs; italics) of relevant bonds are presented.

**Scheme 3:**
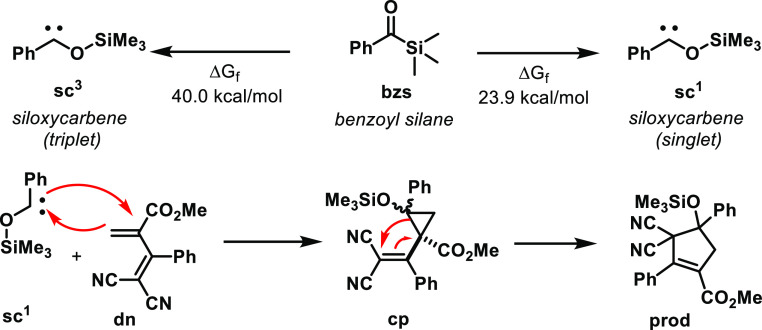
Thermodynamics of Benzoyl Silane-Derived Siloxycarbene Formation and Working Mechanism Investigated by DFT

While the ring enlargement of cyclopropanes has been studied computationally for several systems,^39−41^ similar studies for the expansion of vinylcyclopropanes containing an electron-deficient alkene remain elusive. Hence, mechanistic insights were obtained through computational calculations using density functional theory^42^ (DFT) studies at the M06-2X/6-311++G (d, p)//M06-2X/6-31+G(d, p) level of theory (Figure 1). The comparison of energies of the two siloxycarbenes attainable from benzoyl silane shows large stability of the singlet species **sc**^**1**^ over that of the triplet **sc**^**3**^ (Scheme 3, top). The 16.1 kcal/mol difference is well in agreement with the recent study by Priebbenow.^43^ The computational study proceeded considering the formation of cyclopropane derivatives **cp**, for which the two possible diastereomers were investigated (Scheme 3, bottom). Alternatively, the addition of carbene **sc1** to the terminal carbon of the diene was also considered. Despite the identification of a transition state for the C–C bond formation (with subsequent charge delocalization to the methylenemalononitrile unit), following the intrinsic reaction coordinates invariably resulted in the formation of cyclopropane derivatives. This clearly demonstrates the preference for a route that encompasses cyclopropane as an intermediate.

The interaction of the siloxycarbene with the diene is slightly unfavorable by 5.6 kcal/mol in comparison to the initial pair of reactants. In both cases, **[sc**^**1**^**dn]**_**1**_ and **[sc**^**1**^**dn]**_**2**_, a π–π attractive interaction between the phenyl substituents (3.4–4.4 Å) of both species seems to hold the two reactants together. Despite the overlap of the phenyl substituents in both pairs, the location of the silyl group relative to the methylenemalononitrile differs, with **[sc**^**1**^**dn]**_**1**_ keeping the units close to each other, in contrast with **[sc**^**1**^**dn]**_**2**_ in which one is kept apart from the other. Generally, regardless of the relative geometry of the substituents of the cyclopropane derivatives, the formation of the three-membered rings accounts for the most energy-demanding step of the whole process. The formation of the two diastereomeric cyclopropane derivatives requires the transposition of energy barriers of 11.6 or 11.1 kcal/mol for the formation of the trans (**cp**_**trans**_) or cis (**cp**_**cis**_) diastereomer, respectively. The cyclopropanation transition states are very similar in nature and energies; the forming C–C bonds of the siloxycarbene carbon with C4 are shorter (2.44–2.46 Å) than those forming with C3 (3.14 Å) but still incipient as also confirmed by the weak WIs (WI = 0.1–0.2). The two diastereomeric cyclopropane derivatives differ in energy by 2.0 kcal/mol with a slight preference toward the trans diastereomer, where the repulsion between the phenyl and methylenemalononitrile substituents is diminished. The steric constraints imposed by the cyclopropane substituents are also noticeable in the C–C bond lengths as the C–C bond between the quaternary carbons is the most distended of the cyclopropane ring (1.55 Å in **cp**_**trans**_ and 1.57 Å in **cp**_**cis**_). The cyclopropane rearrangement to the cyclopentene differs slightly depending on the relative positions of the substituents in the cyclopropane. The **ts2**_**trans**_ transition state was calculated for the ring enlargement of **cp**_**trans**_, which is 14.4 kcal/mol less stable than its immediate precursor. Also, in this case, the C–C bonds involved are incipient (2.38–3.06 Å) and weak (WI = 0.1). The formation of the cyclopentane **prod** from the siloxycarbene is highly favored as determined by a Δ*G*_f_ of −54.6 kcal/mol. Despite numerous attempts to calculate a similar synchronous transition state for the ring enlargement from **cp**_**cis**_, such a process seems more likely to proceed through charged intermediates **int**_**1**_ and **int**_**1′**_ due to the steric clash of the bulky phenyl and methylenemalononitrile substituents in the cis positions. Hence, the charged intermediate **int**_**1**_ is reached by the cleavage of the elongated C–C bond between the quaternary carbons of **cp**_**cis**_ (1.57 Å in **cp**_**cis**_ and 2.39 Å in **ts2**_**cis**_) as the C–OSi bond becomes stronger (*d* = 1.38 Å; WI = 0.95 in **cp**_**cis**_ and d = 1.28 Å; WI = 1.29 in **ts2**_**cis**_). A change in conformation in **int**_**1**_ by rotation of the C–C bond between C3 and C4 gives rise to **int**_**1′**_ which undergoes C–C bond formation through a 6.4 kcal/mol energy barrier. The determined transition state **ts3**_**cis**_ is again an early transition one as the forming C–C bond is still very incipient with a 3.30 Å length. When considering the energies involved in both pathways, that is, through each diastereomer of the cyclopropane, they likely compete with each other, and a preference for one of the diastereomeric cyclopropanes is improbable.

**Scheme 4:**
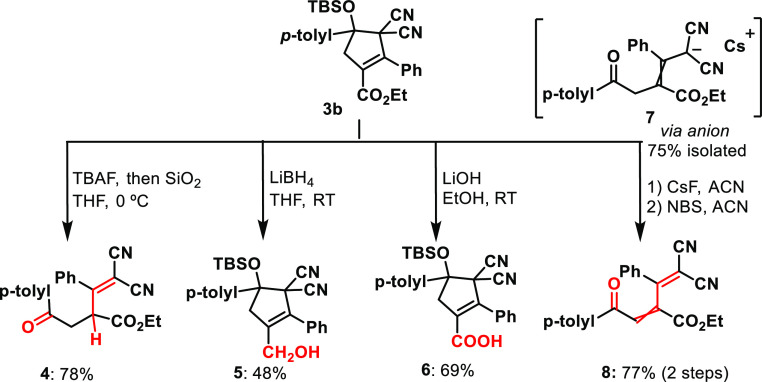
Chemical Transformations of the Siloxycyclopentene Core

After the development of the protocol for the cycloaddition of photogenerated carbene with dicyano-2-methylenebut-3-enoates, we set out to investigate further transformations to the resulting cyclopentene molecules (Scheme 4). TBAF promoted the desilylation of **3b**, followed by purification via silica column chromatography which delivered the ring-opened product **4** in a good yield via acid-promoted retro-aldol. The ester moiety was selectively reduced to the primary alcohol **5** via LiBH_4_ reduction and hydrolyzed to carboxylic acid **6** with LiOH. Attempts at palladium-catalyzed hydrogenation of olefin or its oxidation (*m*-CPBA or H_2_O_2_) invariably led to the recovery of the starting material, demonstrating the remarkable stability of the carbon–carbon double bond. Efforts to reduce nitrile (with DIBAL, LiAlH_4_, or BH_3_) or hydrolyze it under acidic or basic conditions led in all cases to an unidentifiable mixture of compounds. Desilylation under anhydrous conditions using CsF yielded **7** (1:0.2 trans/cis ratio), a room-temperature (RT) stable allylic anion, that could be quenched with equimolar *N*-bromosuccinimide (NBS) to yield the novel diene **8** (as an interconvertible 1:0.2 *cis*/*trans* mixture).

## Conclusions

In summary, we expanded the scope of acylsilane-derived carbene reactivity toward the preparation of new highly functionalized cyclopentene scaffolds, which can be modified without disruption of the cyclic core. To the best of our knowledge, this work presents the first entry on the cycloaddition of photogenerated siloxycarbenes with dienes without the use of transition metal catalysis, paving the way for the use of easily prepared acylsilanes toward the metal-free synthesis of other cyclopentanes. While sensitive to stereochemical constraints, the metal-free cyclopropane ring expansion to cyclopentene proceeds with similar energy requirements for both diastereomeric cyclopropanes, as demonstrated by DFT calculations.

## Experimental Section

### General Information

NMR spectra were recorded using a Bruker Fourier 300 (Bruker, Massachusetts, USA), a Bruker AVANCE III (300 MHz), or a Bruker Fourier 400 (Bruker, Massachusetts, USA) using CDCl_3_, D_2_O, or (CD_3_)_2_SO as a deuterated solvent. All coupling constants are expressed in hertz and chemical shifts (δ) in parts per million. Multiplicities are given as follows: s (singlet), d (doublet), dd (double doublet), dt (double triplet), t (triplet), td (triple triplet), tt (triple triplet), q (quartet), quint (quintuplet), and m (multiplet). Irradiation experiments were performed in a homemade Rayonet-inspired reactor with 16 lamps (419 nm). High-resolution mass spectra were recorded using a Thermo Scientific Q Exactive hybrid quadrupole-Orbitrap mass spectrometer (Thermo Scientific Q Exactive Plus). Reaction mixtures were analyzed by thin layer chromatography (TLC) using Merck silica gel 60F254 aluminum plates and visualized by UV light or stained with potassium permanganate or a phosphomolybdic acid stain. Column chromatography was performed with silica gel Geduran Si 60 (0.040–0.063 mm) purchased from Merck. All solvents were distilled before use. Dry tetrahydrofuran (THF) and dichloromethane (DCM) were obtained from the INERT PureSolv micro apparatus. Toluene was dried by standing in freshly activated 4 Å molecular sieves (20% m/v). Acetonitrile (ACN) was dried by refluxing with CaH. All reagents used were purchased from Fluorochem, Alfa Aesar, TCI, or Sigma-Aldrich. X-ray crystallographic analysis of 3b was conducted using a Bruker D8 VENTURE diffractometer equipped with a Photon 100 complementary metal oxide semiconductor (CMOS) detector and an Oxford Cryostream cooler using graphite monochromated Mo Kα radiation (λ = 0.71073 Å). Dienes **2** were synthesized according to a reported procedure^37^ and used immediately after purification. Dithianes **8** were prepared according to a reported procedure.^44,45^

#### Ethyl 4,4-Dicyano-3-(4-(dimethylamino)phenyl)-2-methylenebut-3-enoate (**2l**)

Following the reported procedure,^37^**2l** was obtained in 12% yield (53 mg) as a red oil. Column eluent hexane/DCM (20:80). ^1^H NMR (300 MHz, CDCl_3_): δ 7.68–7.62 (m, 2H), 6.84 (s, 1H), 6.70–6.64 (m, 2H), 6.03 (s, 1H), 4.19 (q, *J* = 7.1 Hz, 2H), 3.10 (s, 6H), 1.20 (t, *J* = 7.1 Hz, 3H). ^13^C{1H} NMR (75 MHz, CDCl_3_): δ 167.8, 163.8, 153.7, 138.7, 133.9, 132.0, 120.6, 115.3, 114.7, 111.4, 62.1, 40.1, 14.1. HRMS *m*/*z*: [M + H]^+^ calcd for C_17_H_18_N_3_O_2_^+^, 296.1394; found, 296.1383.

#### General Procedure for the Preparation of Silyl Dithianes **9a–f** and **9n**, Adapted from a Reported Procedure^46^

Dithiane **8** (9.5 mmol) was dissolved in 40 mL of dry THF in a dried, argon-filled round-bottom flask. The solution was cooled to −78 °C, and *n*BuLi (2.5 M solution in hexanes, 1.2 equiv, 11.4 mmol) was added dropwise. The solution was stirred at −78 °C for 10 min after which *tert*-butyldimethylsilyl chloride (TBSCl; 11.4 mmol, 1.2 equiv) was added dropwise at this temperature. The solution was stirred at −78 °C for an additional 10 min and then left to warm to RT for a minimum of 1 h. The reaction was quenched with 40 mL of a saturated aqueous NH_4_Cl solution. The layers were separated, and the organic phase was collected. The aqueous phase was extracted with methyl tert-butyl ether (MTBE) (2 × 40 mL), and the organic phases were combined, dried over MgSO_4_, and filtered. After vacuum evaporation of the solvent, the crude was purified via silica column chromatography (eluent hexane/EtOAc mixture) to yield silyldithiane **9**.

#### Trimethyl(2-(*p*-tolyl)-1,3-dithian-2-yl)silane (**9a**)^47^

Following the general procedure, **9a** was obtained in 89% yield (2.38 g) as a colorless oil. Trimethylsilyl chloride was used instead of TBSCl. Column eluent 100% hexane. ^1^H NMR (300 MHz, CDCl_3_): δ 7.79–7.74 (m, 2H), 7.17 (d, *J* = 8.0 Hz, 2H), 2.83–2.74 (m, 2H), 2.45–2.38 (m, 2H), 2.35 (s, 3H), 2.09–1.83 (m, 2H), 0.06 (s, 9H).

#### *tert*-Butyldimethyl(2-(*p*-tolyl)-1,3-dithian-2-yl)silane (**9b**)

Following the general procedure, **9b** was obtained in 89% yield (2.017 g) as a colorless oil. Column eluent 100% hexane. ^1^H NMR (300 MHz, CDCl_3_): δ 7.83 (d, *J* = 8.4 Hz, 2H), 7.17 (d, *J* = 8.0 Hz, 2H), 2.83–2.73 (m, 2H), 2.41–2.35 (m, 2H), 2.35 (s, 3H), 2.08–1.80 (m, 2H), 0.82 (s, 9H), 0.13 (s, 6H). ^13^C{1H} NMR (75 MHz, CDCl_3_): δ 137.6, 135.0, 130.2, 129.2, 48.6, 28.0, 25.3, 25.3, 21.0, 19.9, −6.8. HRMS *m*/*z*: [M + H]^+^ calcd for C_17_H_29_S_2_Si^+^, 325.1474; found, 325.1470.

#### *tert*-Butyldimethyl(2-phenyl-1,3-dithian-2-yl)silane (**9c**)^48^

Following the general procedure, **9c** was obtained in 82% yield (508 mg) as a colorless oil. Column eluent 100% hexane. ^1^H NMR (300 MHz, CDCl_3_): δ 7.99–7.96 (m, 2H), 7.40–7.33 (m, 2H), 7.20–7.15 (m, 1H), 2.83–2.73 (m, 2H), 2.43–2.36 (m, 2H), 2.10–1.82 (m, 2H), 0.81 (s, 9H), 0.15 (s, 6H). HRMS *m*/*z*: [M + H]^+^ calcd for C_16_H_27_S_2_Si^+^, 311.1318; found, 311.1313.

#### *tert*-Butyl(2-(4-fluorophenyl)-1,3-dithian-2-yl)dimethylsilane (**9d**)^49^

Following the general procedure, **9d** was obtained in 73% yield (483 mg) as a colorless oil. Column eluent 100% hexane. ^1^H NMR (300 MHz, CDCl_3_): δ 7.96–7.89 (m, 2H), 7.09–7.02 (m, 2H), 2.80–2.70 (m, 2H), 2.40 (dt, *J* = 14.3, 3.9 Hz, 2H), 2.09–1.83 (m, 2H), 0.83 (s, 9H), 0.13 (s, 6H).

#### (2-(4-Bromophenyl)-1,3-dithian-2-yl)(*tert*-butyl)dimethylsilane (**9e**)

Following the general procedure, **9e** was obtained in 69% yield (536 mg) as a colorless oil. A freshly prepared solution of LDA was used as a base. Column eluent 100% hexane. ^1^H NMR (300 MHz, CDCl_3_): δ 7.87–7.83 (m, 2H), 7.50–7.45 (m, 2H), 2.78–2.68 (m, 2H), 2.43–2.36 (m, 2H), 2.08–1.83 (m, 2H), 0.84 (s, 9H), 0.12 (s, 6H). ^13^C{1H} NMR (75 MHz, CDCl_3_): δ 140.5, 132.2, 131.5, 119.6, 48.4, 28.1, 25.3, 25.2, 20.0, −6.9. HRMS *m*/*z*: [M + H]^+^ calcd for C_16_H_26_BrS_2_Si^+^, 389.0423; found, 389.0417.

#### 4-(2-(*tert*-Butyldimethylsilyl)-1,3-dithian-2-yl)-*N*,*N*-dimethylaniline (**9f**)

Following the general procedure, **9f** was obtained in 94% yield (667 mg) as a white amorphous solid. Column eluent hexane/EtOAc (99:1). ^1^H NMR (300 MHz, CDCl_3_): δ 7.79–7.74 (m, 2H), 6.76–6.71 (m, 2H), 2.97 (s, 6H), 2.82 (td, *J* = 14.1, 2.8 Hz, 2H), 2.36 (dt, *J* = 14.3, 3.9 Hz, 2H), 2.07–1.80 (m, 2H), 0.84 (s, 9H), 0.11 (s, 6H). ^13^C{1H} NMR (75 MHz, CDCl_3_): δ 148.3, 131.1, 128.0, 112.5, 48.4, 40.7, 28.1, 25.5, 25.2, 19.8, −6.8. HRMS *m*/*z*: [M + H]^+^ calcd for C_18_H_32_NS_2_Si^+^, 354.1740; found, 354.1731.

#### *tert*-Butyl(2-(3,4-dimethoxyphenyl)-1,3-dithian-2-yl)dimethylsilane (**9n**)

Following the general procedure, **9f** was obtained in 89% yield (270 mg) as a white amorphous solid. Column eluent hexane/EtOAc (94:6). ^1^H NMR (300 MHz, CDCl_3_): δ 7.57 (d, *J* = 2.4 Hz, 1H), 7.50 (dd, *J* = 8.5, 2.4 Hz, 1H), 6.87 (d, *J* = 8.5 Hz, 1H), 3.90 (s, 3H), 3.89 (s, 3H), 2.81 (td, *J* = 14.0, 2.8 Hz, 2H), 2.40 (dt, *J* = 14.2, 3.7 Hz, 2H), 2.08–1.84 (m, 2H), 0.82 (s, 9H), 0.14 (s, 6H). ^13^C{1H} NMR (75 MHz, CDCl_3_): δ 148.8, 146.9, 133.2, 122.7, 113.8, 110.9, 56.1, 56.0, 48.5, 28.0, 25.4, 25.3, 19.9, −6.7. HRMS *m*/*z*: [M + H]^+^ calcd for C_18_H_31_O_2_S_2_Si^+^, 371.1529; found, 371.1524.

#### General Procedure for the Preparation of Acylsilanes **1a-f** and **1n**, Adapted from a Reported Procedure^50^

Dithiane **9** (3.5 mmol) was dissolved in 17 mL of ACN (sonication and gentle heating were usually required). Then, 5 mL of a saturated aqueous NaHCO_3_ solution was added, and the mixture was cooled to 0 °C. Then, I_2_ (35 mmol, 10 equiv) was added slowly in portions. After addition, the reaction was left at room temperature for 1 h. 20 mL of water was added, followed by continuous addition of NaS_2_O_3_. The mixture was vigorously stirred until the dark brown color of iodine faded to give a bright yellow solution. Then, the aqueous phase was extracted with MTBE (3 × 20 mL), and the organic phases were combined, dried over MgSO_4_, and filtered. After vacuum evaporation of the solvent, the crude was purified via silica column chromatography (eluent hexane/EtOAc mixture) to yield benzoyl silane **1**.

#### *p*-Tolyl(trimethylsilyl)methanone (**1a**)^51^

Following the general procedure, **1a** was obtained in 99% yield (677 mg) as a yellow oil. Column eluent hexane/DCM (6:4). ^1^H NMR (300 MHz, CDCl_3_): δ 7.59 (d, *J* = 8.2 Hz, 2H), 7.13–7.10 (m, 2H), 2.25 (s, 3H), 0.21 (s, 9H).

#### (*tert*-Butyldimethylsilyl)(*p*-tolyl)methanone (**1b**)^52^

Following the general procedure, **1b** was obtained in 77% yield (1.117 g) as a yellow amorphous solid. Column eluent hexane/DCM (8:2). ^1^H NMR (300 MHz, CDCl_3_): δ 7.62 (d, *J* = 8.1 Hz, 2H), 7.16 (d, *J* = 7.9 Hz, 2H), 2.30 (s, 3H), 0.86 (s, 9H), 0.26 (s, 6H). HRMS *m*/*z*: [M + H]^+^ calcd for C_14_H_23_OSi^+^, 235.1513; found, 235.1512.

#### (*tert*-Butyldimethylsilyl)(phenyl)methanone (**1c**)^48^

Following the general procedure, **1c** was obtained in 91% yield (303 mg) as a yellow oil. Column eluent hexane/DCM (8:2). ^1^H NMR (300 MHz, CDCl_3_): δ 7.71–7.68 (m, 2H), 7.47–7.33 (m, 3H), 0.86 (s, 9H), 0.27 (s, 6H). HRMS *m*/*z*: [M + H]^+^ calcd for C_13_H_21_OSi^+^, 221.1356; found, 221.1354.

#### (*tert*-Butyldimethylsilyl)(4-fluorophenyl)methanone (**1d**)^49^

Following the general procedure, **1d** was obtained in 87% yield (291 mg) as a yellow oil. Column eluent hexane/EtOAc (98:2). ^1^H NMR (300 MHz, CDCl_3_): δ 7.77–7.70 (m, 2H), 7.07–6.99 (m, 2H), 0.85 (s, 9H), 0.27 (s, 3H).

#### (4-Bromophenyl)(*tert*-butyldimethylsilyl)methanone (**1e**)

Following the general procedure, **1e** was obtained in 87% yield (350 mg) as a yellow oil. Column eluent hexane/EtOAc (98:2). ^1^H NMR (300 MHz, CDCl_3_): δ 7.58–7.48 (m, 4H), 0.85 (s, 9H), 0.26 (s, 6H). ^13^C{1H} NMR (75 MHz, CDCl_3_): δ 234.7, 141.4, 132.0, 129.2, 127.8, 26.8, 17.1, −4.6. HRMS *m*/***z***: [M + H]^+^ calcd for C_13_H_20_BrOSi^+^, 299.0461; found, 299.0460.

#### (*tert*-Butyldimethylsilyl)(4-(dimethylamino)phenyl)methanone (**1f**)

Following the general procedure, **1f** was obtained in 40% yield (194 mg) as a yellow amorphous sold. Column eluent hexane/EtOAc (85:15). ^1^H NMR (300 MHz, CDCl_3_): δ 7.71–7.66 (m, 2H), 6.59–6.54 (m, 2H), 2.95 (s, 6H), 0.86 (s, 9H), 0.25 (s, 6H). ^13^C{1H} NMR (75 MHz, CDCl_3_): δ 230.5, 153.2, 132.4, 130.3, 110.7, 40.2, 27.0, 17.0, −4.2. HRMS *m*/***z***: [M + H]^+^ calcd for C_15_H_26_NOSi^+^, 264.1778; found, 264.1773.

#### 4-((Trimethylsilyl)carbonyl)benzonitrile (**1g**)

Dithiane **8g** (443 mg, 2 mmol) was dissolved in 9 mL of dry THF in a dried, argon-filled round-bottom flask. The solution was cooled to −78 °C, and *n*BuLi (0.96 mL of 2.5 M solution in hexanes, 1.2 equiv, 2.4 mmol) was added dropwise. The solution was stirred at −78 °C for 10 min after which trimethylsilyl chloride (303 μL, 2.4 mmol, 1.2 equiv) was added dropwise at this temperature. The solution was stirred at −78 °C for an additional 10 min and then left to warm to RT for a minimum of 1 h. The reaction was quenched with 10 mL of a saturated aqueous NH_4_Cl solution. The layers were separated, and the organic phase was collected. The aqueous phase was extracted with MTBE (2 × 10 mL), and the organic phases were combined, dried over MgSO_4_, and filtered. The crude containing **9g** was redissolved in 12 mL of ACN. Then, 4 mL of a saturated aqueous NaHCO_3_ solution was added, and the mixture was cooled to 0 °C. Then, I_2_ (5.08 g, 20 mmol, 10 equiv) was added slowly in portions. After addition, the reaction was left at room temperature for 1 h. Water was added (20 mL), followed by NaS_2_O_3_, and the mixture was vigorously stirred until the dark brown color of iodine faded to give a bright yellow solution. Then, the aqueous phase was extracted with MTBE (3 × 20 mL), and the organic phases were combined, dried over MgSO_4_, and filtered. After vacuum evaporation of the solvent, the crude was purified via silica column chromatography eluent hexane/EtOAc (96:4) to yield acylsilane **1g** in 54% yield (220 mg, 1.08 mmol) as a bright yellow oil, as previously reported.^50^^1^H NMR (300 MHz, CDCl_3_): δ 7.89–7.86 (m, 2H), 7.79–7.76 (m, 2H), 0.38 (s, 9H).

#### *o*-Tolyl(trimethylsilyl)methanone (**1m**)

The benzotriazole hemiaminal ether (535 mg, 2 mmol) was dissolved in dry THF (9 mL) in a dried, argon-filled round-bottom flask. The solution was cooled to −78 °C, and *n*BuLi (0.96 mL of 2.5 M solution in hexanes, 1.2 equiv, 2.4 mmol) was added dropwise. The solution was stirred at −78 °C for 10 min after which trimethylsilyl chloride (303 μL, 2.4 mmol, 1.2 equiv) was added dropwise at this temperature. The solution was stirred at −78 °C for an additional 10 min and then left to warm to RT for a minimum of 1 h. The reaction was quenched with 10 mL of a 1 M HCl solution. The layers were separated, and the organic phase was collected. The aqueous phase was extracted with MTBE (2 × 10 mL), and the organic phases were combined, dried over MgSO_4_, and filtered. The crude oil was redissolved in 10 mL of acetone, and FeCl_3_·6H_2_O was added. After 1 h, acetone was evaporated, and the crude oil was dissolved in 10 mL of hexane and filtered. The liquid was evaporated under reduced pressure, and the crude was purified with the silica column chromatography eluent hexane 100% to give **1m** in 95% yield (364 mg, 1.9 mmol) as a bright yellow oil, with similar spectral characterization to those previously reported.^51^^1^H NMR (300 MHz, CDCl_3_): δ 7.58 (dd, *J* = 7.2, 1.8 Hz, 1H), 7.37–7.22 (m, 3H), 2.42 (s, 3H), 0.32 (s, 9H).

#### (*tert*-Butyldimethylsilyl)(3,4-dimethoxyphenyl)methanone (**1n**)

Following the general procedure, **1n** was obtained in 90% yield (176 mg) as a yellow amorphous solid. Column eluent hexane/EtOAc (92:8). ^1^H NMR (300 MHz, CDCl_3_): δ 7.52 (dd, *J* = 8.3, 1.9 Hz, 1H), 7.36 (d, *J* = 1.9 Hz, 1H), 6.91 (d, *J* = 8.3 Hz, 1H), 3.94 (s, 3H), 3.91 (s, 3H), 0.96 (s, 9H), 0.36 (s, 6H). ^13^C{1H} NMR (75 MHz, CDCl_3_): δ 232.5, 153.1, 149.3, 136.7, 124.6, 110.0, 108.0, 56.2, 55.9, 26.9, 17.0, −4.3. HRMS *m*/*z*: [M + H]^+^ calcd for C_15_H_25_O_3_Si^+^, 281.1567; found, 281.1561.

### General Procedure for the Synthesis of Cyclopentenes **3**

Acylsilane **1** (0.1 mmol) and diene **2** (0.14 mmol, 1.4 equiv) were dissolved in 0.5 mL of dry toluene in a sealed Pasteur pipette. 100 mg of molecular sieves 4 Å was added, and the solution was purged with argon for 15 min and irradiated at 419 nm from a minimum of 24 to a maximum of 72 h. Reaction progress was monitored by TLC and stopped upon full consumption of acylsilane **1**. Toluene was evaporated under reduced pressure, and the crude was purified via silica column chromatography (eluent hexane/EtOAc) to give cyclopentenes **3**.

#### Ethyl 3,3-Dicyano-2-phenyl-4-(*p*-tolyl)-4-((trimethylsilyl)oxy)cyclopent-1-ene-1-carboxylate (**3a**)

Following the general procedure, **3a** was obtained in 66% yield (29.5 mg) as an off-white amorphous solid. 23 h reaction. Column eluent hexane/EtOAc (95:5). ^1^H NMR (300 MHz, CDCl_3_): δ 7.58 (d, *J* = 8.3 Hz, 2H), 7.45–7.38 (m, 5H), 7.27 (d, *J* = 8.0 Hz, 2H), 4.13 (q, *J* = 7.1 Hz, 2H), 3.88 (d, *J* = 17.1 Hz, 1H), 3.36 (d, *J* = 17.1 Hz, 1H), 2.40 (s, 3H), 1.07 (t, *J* = 7.1 Hz, 3H), 0.07 (s, 9H). ^13^C{1H} NMR (75 MHz, CDCl_3_): δ 163.6, 143.7, 140.0, 135.2, 134.5, 131.7, 129.9, 129.6, 128.5, 128.2, 126.6, 112.4, 112.1, 89.0, 61.5, 60.6, 43.4, 21.3, 13.8, 1.3. HRMS *m*/*z*: [M + H]^+^ calcd for C_26_H_29_N_2_O_3_Si^+^, 445.1942; found, 445.1942.

#### Ethyl 4-((*tert*-Butyldimethylsilyl)oxy)-3,3-dicyano-2-phenyl-4-(*p*-tolyl)cyclopent-1-ene-1-carboxylate (**3b**)

Following the general procedure, **3b** was obtained in 74% yield (36.2 mg) as an off-white amorphous solid. 55 h reaction. Column eluent hexane/EtOAc (94:6). ^1^H NMR (300 MHz, CDCl_3_): δ 7.61 (d, *J* = 8.3 Hz, 2H), 7.46–7.36 (m, 5H), 7.27 (d, *J* = 7.9 Hz, 2H), 4.12 (qd, *J* = 7.1, 2.0 Hz, 2H), 3.90 (d, *J* = 17.1 Hz, 1H), 3.36 (d, *J* = 17.0 Hz, 1H), 1.07 (t, *J* = 7.1 Hz, 3H), 0.93 (s, 9H), 0.00 (s, 3H), −0.22 (s, 3H). ^13^C{1H} NMR (75 MHz, CDCl_3_): δ 163.5, 144.1, 140.2, 135.0, 134.5, 131.7, 129.9, 129.7, 128.6, 128.2, 126.9, 112.4, 112.3, 89.0, 61.5, 60.1, 43.1, 25.7, 21.4, 18.5, 13.9, −3.3, −3.5. HRMS *m*/*z*: [M + H]^+^ calcd for C_29_H_35_N_2_O_3_Si^+^, 487.2411; found, 487.2412.

#### Ethyl 4-((*tert*-Butyldimethylsilyl)oxy)-3,3-dicyano-2,4-diphenylcyclopent-1-ene-1-carboxylate (**3c**)

Following the general procedure, **3c** was obtained in 68% yield (32.1 mg) as an off-white amorphous solid. 55 h reaction. Column eluent hexane/EtOAc (94:6). ^1^H NMR (300 MHz, CDCl_3_): δ 7.76–7.73 (m, 2H), 7.49–7.38 (m, 8H), 4.13 (qd, *J* = 7.1, 1.9 Hz, 2H), 3.94 (d, *J* = 17.0 Hz, 2H), 3.40 (d, *J* = 17.0 Hz, 1H), 1.08 (t, *J* = 7.1 Hz, 3H), 0.94 (s, 9H), 0.02 (s, 3H), −0.22 (s, 3H). ^13^C{1H} NMR (75 MHz, CDCl_3_): δ 163.4, 144.0, 138.0, 134.4, 131.6, 130.2, 129.9, 129.0, 128.6, 128.2, 126.9, 112.3, 112.2, 89.0, 61.5, 60.0, 43.0, 25.7, 18.5, 13.8, −3.3, −3.6. HRMS *m*/*z*: [M + H]^+^ calcd for C_28_H_33_N_2_O_3_Si^+^, 473.2255; found, 473.2255.

#### Ethyl 4-((*tert*-Butyldimethylsilyl)oxy)-3,3-dicyano-4-(4-fluorophenyl)-2-phenylcyclopent-1-ene-1-carboxylate (**3d**)

Following the general procedure, **3d** was obtained in 66% yield (32.6 mg) as an off-white amorphous solid. 72 h reaction. Column eluent hexane/EtOAc (93:7). ^1^H NMR (300 MHz, CDCl_3_): δ 7.77–7.71 (m, 2H), 7.46–7.37 (m, 5H), 7.21–7.14 (m, 2H), 4.12 (qd, *J* = 7.1, 1.8 Hz, 2H), 3.89 (d, *J* = 17.1 Hz, 1H), 3.39 (d, *J* = 17.0 Hz, 1H), 1.07 (t, *J* = 7.1 Hz, 3H), 0.94 (s, 9H), 0.04 (s, 3H), −0.21 (s, 3H). ^13^C{1H} NMR (75 MHz, CDCl_3_): δ 165.3, 163.3, 162.0, 144.0, 134.2, 134.1, 134.1, 131.5, 130.0, 129.1, 128.9, 128.6, 128.2, 116.2, 116.0, 112.1, 112.1, 88.5, 61.5, 60.1, 43.1, 25.7, 18.5, 13.8, −3.2, −3.5. ^19^F{1H} NMR (376 MHz, CDCl_3_): δ −110.6. HRMS *m*/*z*: [M]^+^ calcd for C_28_H_31_FN_2_O_3_Si^+^, 490.2082; found, 490.2082.

#### Ethyl 4-(4-Bromophenyl)-4-((*tert*-butyldimethylsilyl)oxy)-3,3-dicyano-2-phenylcyclopent-1-ene-1-carboxylate (**3e**)

Following the general procedure, **3e** was obtained in 66% yield (36.5 mg) as an off-white amorphous solid. 55 h reaction. Column eluent hexane/EtOAc (90:10). ^1^H NMR (300 MHz, CDCl_3_): δ 7.62 (s, 4H), 7.46–7.37 (m, 5H), 4.12 (qd, *J* = 7.1, 1.9 Hz, 2H), 3.87 (d, *J* = 17.1 Hz, 1H), 3.38 (d, *J* = 17.0 Hz, 1H), 1.07 (t, *J* = 7.1 Hz, 3H), 0.94 (s, 9H), 0.05 (s, 3H), −0.19 (s, 3H). ^13^C{1H} NMR (75 MHz, CDCl_3_): δ 163.3, 143.9, 137.1, 134.2, 132.3, 131.4, 130.0, 128.6, 128.6, 128.2, 124.6, 112.0, 112.0, 88.5, 61.6, 60.0, 42.8, 25.7, 18.5, 13.8, −3.2, −3.5. HRMS *m*/*z*: [M + H]^+^ calcd for C_28_H_32_BrN_2_O_3_Si^+^, 551.1360; found, 551.1361.

#### Ethyl 4-((*tert*-Butyldimethylsilyl)oxy)-3,3-dicyano-4-(4-(dimethylamino)phenyl)-2-phenylcyclopent-1-ene-1-carboxylate (**3f**)

Following the general procedure, **3f** was obtained in 62% yield (32 mg) as an off-white amorphous solid. 48 h reaction. Column eluent hexane/DCM (65:35). ^1^H NMR (300 MHz, CDCl_3_): δ 7.55 (d, *J* = 8.9 Hz, 2H), 7.45–7.37 (m, 5H), 6.73 (d, *J* = 9.0 Hz, 2H), 4.11 (qd, *J* = 7.1, 2.1 Hz, 2H), 3.87 (d, *J* = 17.0 Hz, 1H), 3.33 (d, *J* = 17.0 Hz, 1H), 3.01 (s, 6H), 1.07 (t, *J* = 7.1 Hz, 3H), 0.93 (s, 9H), −0.00 (s, 3H), −0.17 (s, 3H). ^13^C{1H} NMR (75 MHz, CDCl_3_): δ 163.7, 151.2, 144.2, 134.5, 131.9, 129.7, 128.5, 128.2, 128.0, 124.8, 112.7, 112.5, 111.8, 89.3, 61.3, 60.3, 43.3, 40.2, 25.7, 18.5, 13.8, −3.3, −3.5. HRMS *m*/*z*: [M + H]^+^ calcd for C_30_H_38_N_3_O_3_Si^+^, 516.26770; found, 516.2670.

#### Ethyl 4-((*tert*-Butyldimethylsilyl)oxy)-3,3-dicyano-2-(4-fluorophenyl)-4-phenylcyclopent-1-ene-1-carboxylate (**3h**)

Following the general procedure, **3h** was obtained in 61% yield (30.2 mg) as an off-white amorphous solid. 55 h reaction. Column eluent hexane/EtOAc (93:7). ^1^H NMR (300 MHz, CDCl_3_): δ 7.75–7.71 (m, 2H), 7.49–7.47 (m, 3H), 7.42–7.38 (m, 2H), 7.19–7.11 (m, 2H), 4.15 (qd, *J* = 7.1, 2.4 Hz, 2H), 3.92 (d, *J* = 17.1 Hz, 1H), 3.39 (d, *J* = 17.0 Hz, 1H), 1.12 (t, *J* = 7.1 Hz, 3H), 0.93 (s, 9H), 0.00 (s, 3H), −0.23 (s, 3H). ^13^C{1H} NMR (75 MHz, CDCl_3_): δ 165.3, 163.2, 162.0, 143.1, 137.8, 134.9, 130.5, 130.3, 130.3, 129.1, 127.6, 127.5, 126.9, 116.0, 115.7, 112.3, 112.1, 89.0, 61.6, 60.0, 43.0, 25.7, 18.5, 13.9, −3.3, −3.6. ^19^F{1H} NMR (376 MHz, CDCl_3_): δ −110.3 HRMS *m*/*z*: [M]^+^ calcd for C_28_H_31_FN_2_O_3_Si^+^, 490.2082; found, 490.2082.

#### Ethyl 4-((*tert*-Butyldimethylsilyl)oxy)-2-(4-chlorophenyl)-3,3-dicyano-4-phenylcyclopent-1-ene-1-carboxylate (**3i**)

Following the general procedure, **3i** was obtained in 77% yield (39.1 mg) as an off-white amorphous solid. 55 h reaction. Column eluent hexane/EtOAc (92:8). ^1^H NMR (300 MHz, CDCl_3_): δ 7.74–7.71 (m, 2H), 7.50–7.42 (m, 5H), 7.36–7.33 (m, 2H), 4.15 (qd, *J* = 7.1, 2.3 Hz, 2H), 3.92 (d, *J* = 17.2 Hz, 1H), 3.39 (d, *J* = 17.1 Hz, 1H), 1.13 (t, *J* = 7.1 Hz, 3H), 0.93 (s, 9H), 0.00 (s, 3H), −0.23 (s, 3H). ^13^C{1H} NMR (75 MHz, CDCl_3_): δ 163.1, 142.9, 137.8, 136.2, 135.1, 130.3, 130.0, 129.7, 129.1, 129.0, 126.9, 112.2, 112.0, 89.0, 61.7, 59.8, 43.0, 25.7, 18.5, 13.9, −3.3, −3.6. HRMS *m*/*z*: [M + H]^+^ calcd for C_28_H_32_ClN_2_O_3_Si^+^, 507.1865; found, 507.1825.

#### Ethyl 2-(4-Bromophenyl)-4-((*tert*-butyldimethylsilyl)oxy)-3,3-dicyano-4-phenylcyclopent-1-ene-1-carboxylate (**3j**)

Following the general procedure, **3j** was obtained in 72% yield (40.0 mg) as an off-white amorphous solid. 55 h reaction. Column eluent hexane/DCM (60:40). ^1^H NMR (300 MHz, CDCl_3_): δ 7.62 (s, 4H), 7.46–7.44 (m, 3H), 7.41–7.36 (m, 2H), 4.12 (qd, *J* = 7.1, 1.9 Hz, 2H), 3.87 (d, *J* = 17.1 Hz, 1H), 3.38 (d, *J* = 17.0 Hz, 1H), 1.07 (t, *J* = 7.1 Hz, 3H), 0.94 (s, 9H), 0.05 (s, 3H), −0.19 (s, 3H). ^13^C{1H} NMR (75 MHz, CDCl_3_): δ 163.3, 143.9, 137.1, 134.2, 132.3, 131.4, 130.0, 128.6, 128.6, 128.2, 124.6, 112.0, 112.0, 88.5, 61.6, 60.0, 42.8, 25.7, 18.5, 13.8, −3.2, −3.5. HRMS *m*/*z*: [M]^+^ calcd for C_28_H_31_BrN_2_O_3_Si^+^, 550.1282; found, 550.1284.

#### Ethyl 4-((*tert*-Butyldimethylsilyl)oxy)-3,3-dicyano-2-(4-methoxyphenyl)-4-phenylcyclopent-1-ene-1-carboxylate (**3k**)

Following the general procedure, **3k** was obtained in 66% yield (33.2 mg) as an off-white amorphous solid. 72 h reaction. Column eluent hexane/EtOAc (90:10). ^1^H NMR (300 MHz, CDCl_3_): δ 7.76–7.72 (m, 2H), 7.50–7.45 (m, 3H), 7.40–7.35 (m, 2H), 6.99–6.94 (m, 2H), 4.16 (qd, *J* = 7.1, 2.0 Hz, 2H), 3.91 (d, *J* = 17.0 Hz, 1H), 3.84 (s, 3H), 3.37 (d, *J* = 17.0 Hz, 1H), 0.92 (s, 9H), 0.01 (s, 3H), −0.23 (s, 3H). ^13^C{1H} NMR (75 MHz, CDCl_3_): δ 163.6, 160.9, 144.1, 138.0, 133.1, 130.1, 129.9, 129.0, 127.0, 123.7, 114.0, 112.6, 112.4, 88.9, 61.4, 59.9, 55.4, 43.1, 25.7, 18.5, 14.0, −3.3, −3.6. HRMS *m*/*z*: [M + H]^+^ calcd for C_29_H_35_N_2_O_4_Si^+^, 503.2361; found, 503.2357.

#### Ethyl 3,3-Dicyano-2-phenyl-4-(*o*-tolyl)-4-((trimethylsilyl)oxy)cyclopent-1-ene-1-carboxylate (**3m**)

Following the general procedure, **3m** was obtained in 40% yield (17.7 mg) as an off-white amorphous solid. 72 h reaction. Column eluent hexane/EtOAc (93:7). ^1^H NMR (300 MHz, CDCl_3_): δ 7.50–7.22 (m, 9H), 4.15–4.08 (m, 2H), 3.94 (d, *J* = 17.0 Hz, 1H), 3.50 (d, *J* = 17.0 Hz, 1H), 2.70 (s, 3H), 1.04 (t, *J* = 7.1 Hz, 3H), 0.10 (s, 9H). ^13^C{1H} NMR (75 MHz, CDCl_3_): δ 163.5, 142.9, 138.9, 136.4, 135.2, 133.8, 131.6, 129.8, 129.7, 128.5, 128.3, 128.0, 126.2, 112.7, 112.3, 91.1, 61.6, 60.0, 46.2, 24.3, 13.8, 1.4. HRMS *m*/*z*: [M + H]^+^ calcd for C_26_H_29_N_2_O_3_Si^+^, 445.1942; found, 445.1942.

#### Ethyl 4-((*tert*-Butyldimethylsilyl)oxy)-3,3-dicyano-4-(3,4-dimethoxyphenyl)-2-phenylcyclopent-1-ene-1-carboxylate (**3n**)

Following the general procedure, **3n** was obtained in 80% yield (42.5 mg) as an off-white amorphous solid. 48 h reaction. Column eluent hexane/EtOAc (92:8). ^1^H NMR (300 MHz, CDCl_3_): δ 7.45–7.35 (m, 6H), 7.19 (dd, *J* = 8.4, 2.3 Hz, 1H), 6.90 (d, *J* = 8.4 Hz, 1H), 4.19–4.04 (m, 2H), 3.93 (s, 3H), 3.92 (s, 3H), 3.90 (d, *J* = 17.0 Hz, 1H), 3.34 (d, *J* = 17.0 Hz, 1H), 1.06 (t, *J* = 7.1 Hz, 3H), 0.95 (s, 9H), 0.03 (s, 3H), −0.18 (s, 3H). ^13^C{1H} NMR (75 MHz, CDCl_3_): δ 163.5, 150.3, 149.2, 144.1, 134.3, 131.7, 130.3, 129.9, 128.6, 128.1, 119.3, 112.4, 112.3, 110.5, 110.0, 89.0, 61.4, 60.3, 56.0, 55.9, 42.9, 25.7, 18.5, 13.8, −3.3, −3.5. HRMS *m*/*z*: [M + H]^+^ calcd for C_30_H_37_N_2_O_5_Si^+^, 533.2466; found, 533.2463.

#### Ethyl 2-(4-Bromophenyl)-4-((*tert*-butyldimethylsilyl)oxy)-3,3-dicyano-4-(3,4-dimethoxyphenyl)cyclopent-1-ene-1-carboxylate (**3o**)

Following the general procedure, **3o** was obtained in 90% yield (54.8 mg) as an off-white amorphous solid. 48 h reaction. Column eluent hexane/EtOAc (85:15). ^1^H NMR (400 MHz, CDCl_3_): δ 7.61 (d, *J* = 8.2 Hz, 2H), 7.35 (d, *J* = 2.3 Hz, 1H), 7.28 (d, *J* = 8.3 Hz, 2H), 7.17 (dd, *J* = 8.4, 2.3 Hz, 1H), 6.92 (d, *J* = 8.4 Hz, 1H), 4.20–4.12 (m, 2H), 3.94 (s, 6H), 3.90 (d, *J* = 17.2 Hz, 1H), 3.35 (d, *J* = 17.1 Hz, 1H), 1.14 (t, *J* = 7.1 Hz, 3H), 0.95 (s, 9H), 0.04 (s, 3H), −0.16 (s, 3H). ^13^C{1H} NMR (100 MHz, CDCl_3_): δ 163.1, 150.3, 149.2, 142.9, 135.0, 131.9, 130.5, 130.0, 129.8, 124.4, 119.2, 112.2, 112.1, 110.5, 109.8, 89.1, 61.6, 60.0, 56.0, 55.9, 42.8, 25.6, 18.4, 13.9, −3.4, −3.6. HRMS *m*/*z*: [M + H]^+^ calcd for C_30_H_36_BrN_2_O_5_Si^+^, 611.1571; found, 611.1583.

#### Ethyl 2-(4-Bromophenyl)-4-((tert-butyldimethylsilyl)oxy)-3,3-dicyano-4-(*p*-tolyl)cyclopent-1-ene-1-carboxylate (**3p**)

Following the general procedure, **3p** was obtained in 69% yield (39.0 mg) as a yellow amorphous solid. 48 h reaction. Column eluent hexane/EtOAc (85:15). ^1^H NMR (400 MHz, CDCl_3_): δ 7.57 (d, *J* = 8.0 Hz, 4H), 7.25 (d, *J* = 8.1 Hz, 4H), 4.18–4.07 (m, 2H), 3.87 (d, *J* = 17.1 Hz, 1H), 3.33 (d, *J* = 17.1 Hz, 1H), 2.38 (s, 3H), 1.10 (t, *J* = 7.1 Hz, 3H), 0.90 (s, 9H), −0.03 (s, 3H), −0.24 (s, 3H). ^13^C{1H} NMR (100 MHz, CDCl_3_): δ 163.1, 142.9, 140.3, 135.1, 134.8, 131.9, 130.5, 129.9, 129.7, 126.8, 124.4, 112.2, 112.1, 89.0, 61.6, 59.8, 43.1, 25.7, 21.4, 18.4, 13.9, −3.3, −3.6. HRMS *m*/*z*: [M + H]^+^ calcd for C_29_H_34_BrN_2_O_3_Si^+^, 565.1517; found, 565.1526.

#### Ethyl 2,4-Bis(4-bromophenyl)-4-((*tert*-butyldimethylsilyl)oxy)-3,3-dicyanocyclopent-1-ene-1-carboxylate (**3q**)

Following the general procedure, **3q** was obtained in 87% yield (52.4 mg) as a white amorphous solid. 72 h reaction. Column eluent hexane/DCM (68:32). ^1^H NMR (400 MHz, CDCl_3_): δ 7.63–7.57 (m, 6H), 7.25 (d, *J* = 8.3 Hz, 2H), 4.20–4.08 (m, 2H), 3.85 (d, *J* = 17.1 Hz, 1H), 3.35 (d, *J* = 17.1 Hz, 1H), 1.11 (t, *J* = 7.1 Hz, 3H), 0.91 (s, 9H), 0.02 (s, 3H), −0.21 (s, 3H). ^13^C{1H} NMR (100 MHz, CDCl_3_): δ 162.9, 142.7, 136.9, 134.9, 132.3, 131.9, 130.2, 129.8, 128.5, 124.7, 124.6, 111.8 (2), 88.5, 61.8, 59.7, 42.8, 25.6, 18.4, 13.9, −3.2, −3.5. HRMS *m*/*z*: [M + H]^+^ calcd for C_28_H_31_Br_2_N_2_O_3_Si^+^, 631.0445; found, 631.0461.

#### Ethyl 4-(4-Bromophenyl)-4-((*tert*-butyldimethylsilyl)oxy)-2-(4-chlorophenyl)-3,3-dicyanocyclopent-1-ene-1-carboxylate (**3r**)

Following the general procedure, **3r** was obtained in 69% yield (40.6 mg) as a pale oil. 72 h reaction. Column eluent hexane/DCM (70:30). ^1^H NMR (400 MHz, CDCl_3_): δ 7.64–7.59 (m, 4H), 7.44 (d, *J* = 8.3 Hz, 2H), 7.33 (d, *J* = 8.3 Hz, 2H), 4.18–4.09 (m, 2H), 3.87 (d, *J* = 17.1 Hz, 1H), 3.37 (d, *J* = 17.1 Hz, 1H), 1.13 (t, *J* = 7.1 Hz, 3H), 0.93 (s, 9H), 0.03 (s, 3H), −0.20 (s, 3H). ^13^C{1H} NMR (100 MHz, CDCl_3_): δ 163.0, 142.8, 136.9, 136.3, 134.9, 132.3, 129.7, 129.6, 129.0, 128.5, 124.8, 111.9 (2), 88.5, 61.8, 59.8, 42.8, 25.6, 18.4, 13.9, −3.2, −3.5. HRMS *m*/*z*: [M + H]^+^ calcd for C_28_H_31_BrClN_2_O_3_Si^+^, 585.0970; found, 585.0979.

#### Ethyl 4,4-Dicyano-2-(2-oxo-2-(*p*-tolyl)ethyl)-3-phenylbut-3-enoate (**4**)

Cyclopentene **3b** (10 mg, 20 μmol) was dissolved in THF (1 mL) in a round-bottom flask. The solution was cooled to 0 °C, and TBAF·H_2_O (7.1 mg, 23 μmol, 1.1 equiv) was added. After 10 min, at 0 °C, the solution was warmed to RT. Ketone **4** was purified by silica column flash chromatography with dry loading using hexane/EtOAc (80:20) as an eluent to give **4** as an amorphous off-white solid in 78% yield (5.8 mg, 16 μmol). ^1^H NMR (300 MHz, CDCl_3_): δ 7.72 (d, *J* = 8.2 Hz, 2H), 7.51–7.39 (m, 3H), 7.27–7.20 (m, 4H), 4.83 (dd, *J* = 8.3, 5.5 Hz, 1H), 4.31 (q, *J* = 7.1 Hz, 2H), 3.74 (dd, *J* = 18.4, 5.5 Hz, 1H), 3.17 (dd, *J* = 18.4, 8.4 Hz, 1H), 2.39 (s, 3H), 1.33 (t, *J* = 7.1 Hz, 3H). ^13^C{1H} NMR (75 MHz, CDCl_3_): δ 195.4, 175.2, 168.6, 145.0, 133.6, 133.3, 131.8, 129.6, 129.4, 128.3, 127.6, 112.4, 112.1, 90.0, 63.0, 47.9, 38.7, 21.8, 14.2. HRMS *m*/*z*: [M + H]^+^ calcd for C_23_H_21_N_2_O_3_^+^, 373.1547; found, 373.1544.

#### 5-((*tert*-Butyldimethylsilyl)oxy)-3-(hydroxymethyl)-2-phenyl-5-(*p*-tolyl)cyclopent-2-ene-1,1-dicarbonitrile (**5**)

Cyclopentene **3b** (20 mg, 41 μmol) was dissolved in dry THF (2 mL) in a round-bottom flask, and the resulting solution was cooled to 0 °C. LiBH_4_ (3 mg, 135 μmol, 3.3 equiv) was added, and the reaction was warmed to RT. After 3 h, water (9.7 μL, 540 μmol, 13.2 equiv) was added, and the solution was left to stir for 5 min. Then, the solvent was evaporated, and the crude was purified by silica flash column chromatography using hexane/EtOAc as an eluent (80:20) to give alcohol **5** in 48% yield (8.8 mg, 20 μmol). ^1^H NMR (300 MHz, CDCl_3_): δ 7.61 (d, *J* = 8.3 Hz, 2H), 7.47–7.41 (m, 3H), 7.34–7.32 (m, 2H), 7.25 (d, *J* = 6.7 Hz, 2H), 4.44–4.33 (m, 2H), 3.76 (d, *J* = 17.0 Hz, 1H), 3.21 (d, *J* = 17.0 Hz, 1H), 2.39 (s, 3H), 0.92 (s, 9H), 0.01 (s, 3H), −0.23 (s, 3H). ^13^C{1H} NMR (100 MHz, CDCl_3_): δ 144.1, 139.9, 135.9, 132.0, 131.5, 129.6, 129.4, 129.1, 128.6, 127.1, 113.6, 113.6, 89.2, 59.7, 58.7, 43.6, 25.8, 21.4, 18.5, −2.9, −3.4. HRMS *m*/*z*: [M + H]^+^ calcd for C_27_H_33_N_2_O_2_Si^+^, 445.2306; found, 445.2304.

#### 4-((*tert*-Butyldimethylsilyl)oxy)-3,3-dicyano-2-phenyl-4-(*p*-tolyl)cyclopent-1-ene-1-carboxylic Acid (**6**)

Cyclopentenone **3b** (6 mg, 12 μmol) was dissolved in EtOH (1 mL). LiOH (20 μL of 1 M aqueous solution, 20 μmol, 2 equiv) was added, and the solution was stirred for 5 h at RT. Then, the solvent was evaporated, and the crude was purified by silica flash column chromatography using hexane/EtOAc/AcOH (60:40:0.1) as an eluent to give carboxylic acid **6** in 69% yield (3.8 mg, 8.3 μmol). ^1^H NMR (400 MHz, CDCl_3_): δ 7.59 (d, *J* = 8.3 Hz, 2H), 7.47–7.39 (m, 5H), 7.27 (d, *J* = 6.6 Hz, 2H), 3.90 (d, *J* = 17.1 Hz, 1H), 3.36 (d, *J* = 17.0 Hz, 1H), 2.40 (s, 3H), 0.92 (s, 9H), −0.00 (s, 3H), −0.23 (s, 3H). ^13^C{1H} NMR (100 MHz, CDCl_3_): δ 167.3, 146.3, 140.3, 134.8, 133.4, 131.3, 130.2, 129.8, 128.8, 128.2, 126.9, 112.2, 112.1, 88.9, 60.3, 43.2, 25.7, 21.4, 18.5, −3.2, −3.5. HRMS *m*/*z*: [M + H]^+^ calcd for C_27_H_31_N_2_O_3_Si^+^, 459.2098; found, 459.2091.

#### Cesium 1,1-Dicyano-3-(ethoxycarbonyl)-5-oxo-2-phenyl-5-(*p*-tolyl)pent-2-en-1-ide (**7**)

Cyclopentene **3b** (10 mg, 20 μmol) was dissolved in dry ACN (1 mL) in an oven-dried round-bottom flask under an argon atmosphere. CsF (94 mg, 620 μmol, 30 equiv) was added, and the solution was left to stir for 3 h. The solvent was evaporated, and the crude mixture was redissolved in acetone. The solid CsF excess was filtered off through celite, and the filtrate was evaporated to give salt **7** as a bright yellow oil in 75% yield (7.6 mg, 15 μmol) as a 1:0.2 mixture of *E*/*Z* isomers. **7** can be converted to **4** simply via passing through a silica plug. **7a** (major): ^1^H NMR (400 MHz, (CD_3_)_2_CO): δ 7.94 (d, *J* = 8.2 Hz, 2H), 7.28 (d, *J* = 7.9 Hz, 2H), 7.21 (s, 5H), 4.39 (s, 2H), 3.52 (q, *J* = 7.1 Hz, 2H), 2.39 (s, 3H), 0.59 (t, *J* = 7.1 Hz, 3H). ^13^C{1H} NMR (100 MHz, (CD_3_)_2_CO): δ 199.6, 169.8, 158.2, 144.9, 143.2, 136.8, 130.2, 129.7, 129.0, 127.9, 127.8, 125.2, 102.0, 58.7, 43.1, 40.2, 21.6, 14.1. **7b** (minor): ^1^H NMR (400 MHz, (CD_3_)_2_CO): δ 7.70 (d, *J* = 8.2 Hz, 2H), 7.29–7.20 (m, 7H), 4.06 (q, *J* = 7.1 Hz, 2H), 3.60 (s, 2H), 2.33 (s, 3H), 1.12 (t, *J* = 7.1 Hz, 3H). HRMS *m*/*z*: [M+2H]^+^ calcd for C_23_H_21_N_2_O_3_Si^+^, 373.1547; found, 373.1544.

#### Ethyl 2-(2,2-Dicyano-1-phenylvinyl)-4-oxo-4-(*p*-tolyl)but-2-enoate (**8**)

Cyclopentene **3b** (20 mg, 40 μmol) was dissolved in dry ACN (2 mL) in an oven-dried round-bottom flask under an argon atmosphere. CsF (180 mg, 1.2 mmol, 30 equiv) was added, and the solution was left to stir for 3 h. Excess CsF was filtered through celite, and NBS (7.1 mg, 40 μmol, 1 equiv) was added to the filtrate solution. The bright yellow color of intermediate **7** quickly fades. The solvent was quickly evaporated. Analysis of the ^1^H NMR crude at this stage reveals a *E*/*Z* ratio of 0.45:1. The crude was purified by silica flash column chromatography using hexane/EtOAc as an eluent (80:20) to give diene **8** in 77% yield (11.4 mg, 31 μmol) in a *E*/*Z* ratio of 1:0.2 at thermodynamic equilibrium (Figure S2). **8a** (major): ^1^H NMR (400 MHz, (CD_3_)_2_CO): δ 8.41 (s, 1H), 7.96 (d, *J* = 8.2 Hz, 2H), 7.78–7.76 (m, 2H), 7.69–7.53 (m, 3H), 7.40 (d, *J* = 8.2 Hz, 2H), 4.31 (q, *J* = 7.1 Hz, 2H), 2.43 (s, 3H), 1.24 (t, *J* = 7.1 Hz, 3H). ^13^C{1H} NMR (100 MHz, (CD_3_)_2_CO): δ 189.1, 172.2, 163.4, 146.7, 139.8, 138.7, 134.4, 134.2, 133.6, 130.6, 130.2, 129.9, 129.8, 113.5, 113.4, 85.4, 63.6, 21.7, 14.1. **8b** (minor): ^1^H NMR (400 MHz, (CD_3_)_2_CO): δ 7.99–7.39 (m, 10H), 3.96 (q, *J* = 7.1 Hz, 2H), 2.43 (s, 3H), 0.89 (t, *J* = 7.1 Hz, 3H). HRMS *m*/*z*: [M + H]^+^ calcd for C_23_H_19_N_2_O_3_, 371.1390; found, 371.1384. Note: Prolonged heating of the reaction crude at 40 °C also delivers the same relative *E*/*Z* ratio of isomers, further confirming the thermodynamic equilibrium. Identification of isomers was conducted via analysis of the ethyl ester β-hydrogen which shows characteristic lower field shift from *trans* to *cis* conformation (relative to ester) from 7.79 to 8.41 ppm.

## Supporting Information Available

Full accounts on computational calculations, X-ray crystallographic analysis for **3b**, and ^1^H and ^13^C NMR copies of spectra for all novel compounds (PDF)
