# Supplementary material for: Functionalized Cyclopentenes via the Formal [4+1] Cycloaddition of Photogenerated Siloxycarbenes from Acyl Silanes
Source: J Org Chem. 2022 Jun 23;87(14):8910–20. doi: 10.1021/acs.joc.2c00591 (PMC9776530; doi:10.1021/acs.joc.2c00591)
Supplement: Supplementary file 1 — jo2c00591_si_001.pdf [file jo2c00591_si_001.pdf]

Supporting Information

Functionalized cyclopentenes via formal [4+1] cycloaddition of photogenerated-siloxycarbenes from acyl silanes

João R. Vale[a,b], Rafael F. Gomes[a], Carlos A. M. Afonso[a]* and Nuno R. Candeias[b,c]*

a) iMed.ULisboa, Faculty of Pharmacy, University of Lisbon, Av. Prof. Gama Pinto, 1649-003 Lisbon, Portugal; b) Faculty of Engineering and Natural Sciences, Tampere University, Korkeakoulunkatu 8, 33101 Tampere, Finland; c) LAQV-REQUIMTE, Department of Chemistry, University of Aveiro, 3810-193 Aveiro, Portugal

carlosafonso@ff.ulisboa.pt ; ncandeias@ua.pt ;

Table of contents:

Milimolar scale synthesis of **3b:** S2

Crystal sample preparation of **3b**: S2

S-Figure 1: ORTEP-3 diagram of **3b** S2

S-Table 1: Crystallographic data for **3b** S2

S-Figure 2: ^{1}H NMR spectra of the reaction crude from synthesis of **8** and after purification S3

Computational Details S3

NMR spectra of novel compounds: S5

Atomic coordinates for all the optimized species (M06-2X/6-31+G**) S36

References S43

Milimolar scale synthesis of **3b:**

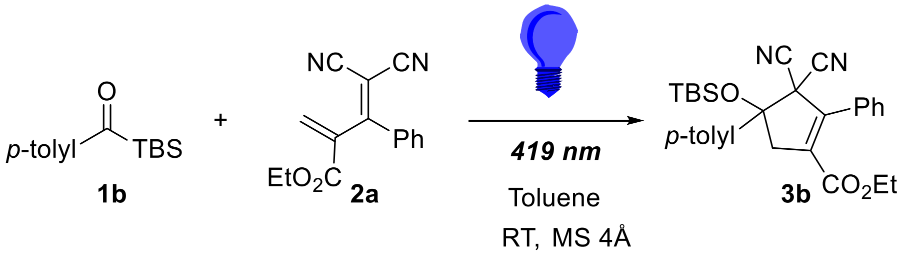

Acylsilane **1b** (258 mg, 1.1 mmol) and diene **2a** (388 mg, 1.54 mmol, 1.4 equiv.) were dissolved in 5.5 mL of dry toluene in an oven-dried vial. The solution was purged with argon for 15 minutes, transferred to three sealed Pasteur pipettes and irradiated at 419 nm for 72 h. Toluene was evaporated under reduced pressure and the crude purified via silica column chromatography, eluent hexane/EtOAc (94:6) to give cyclopentene **3b** in 80% yield (426 mg, 0.875 mmol).

Crystal sample preparation of **3b**:

5 mg of **3b** were dissolved in 0.2 mL of EtOAc in a small vial. The vial was encapsuled in a larger vial filled with 2 mL of hexane and closed with a lid. Suitable crystals formed overnight, which were collected and washed with hexane. X-ray crystallographic analysis of **3b** was conducted using Bruker D8 Venture diffractometer equipped with a Photon 100 CMOS detector and an Oxford Cryostem Cooler, using graphite monochromated Mo-Kα radiation (λ= 0.71073 Å).

S-Figure 1: ORTEP-3 diagram of **3b** (asymmetric unit, CCDC: 2142958), using 50% probability level ellipsoids:

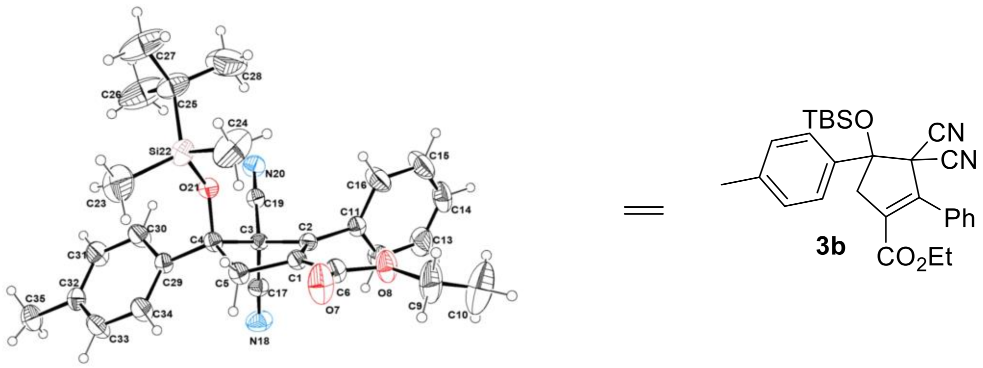

S-Table 1: Crystallographic data for **3b** (CCDC: 2142958):

Empirical formula	C29 H34 N2 O3 Si	
Formula weight	486.67	
Temperature	296(2) K	
Wavelength	0.71073 Å	
Crystal system	Triclinic	
Space group	P -1	
Unit cell dimensions	a = 8.9516(8) Å<br>b = 13.0406(12) Å<br>c = 13.7704(14) Å	α= 112.719(4)°.<br>β= 94.275(4)°.<br>γ = 96.195(4)°.
Volume	1462.2(2) $Å^3$	
Z	2	
Density (calculated)	1.105 Mg/m^3	
Absorption coefficient	0.110 mm^{-1}	
F(000)	520	

Crystal size	0.300 x 0.200 x 0.200 mm^3
Theta range for data collection	2.981 to 27.542°.
Index ranges	-11<=h<=11, -16<=k<=16, -17<=l<=17
Reflections collected	65903
Independent reflections	6714 [R(int) = 0.1193]
Completeness to theta = 25.242°	99.8 %
Absorption correction	Semi-empirical from equivalents
Max. and min. transmission	0.7456 and 0.6704
Refinement method	Full-matrix least-squares on F2
Data / restraints / parameters	6714 / 0 / 323
Goodness-of-fit on F2	.047
Final R indices [I>2sigma(I)]	R1 = 0.0749, wR2 = 0.1634
R indices (all data)	R1 = 0.1548, wR2 = 0.1858
Extinction coefficient	n/a
Extinction coefficient	0.428 and -0.367 e.$Å^{-3}$

S-Figure 2: ^{1}H NMR spectrum (300 MHz, $(CD_3)_2CO$) of the reaction crude from synthesis of **8** (top) and ^{1}H NMR spectrum (300 MHz, $(CD_3)_2CO$) of **8** after purification (bottom), showing thermodynamic interconversion.

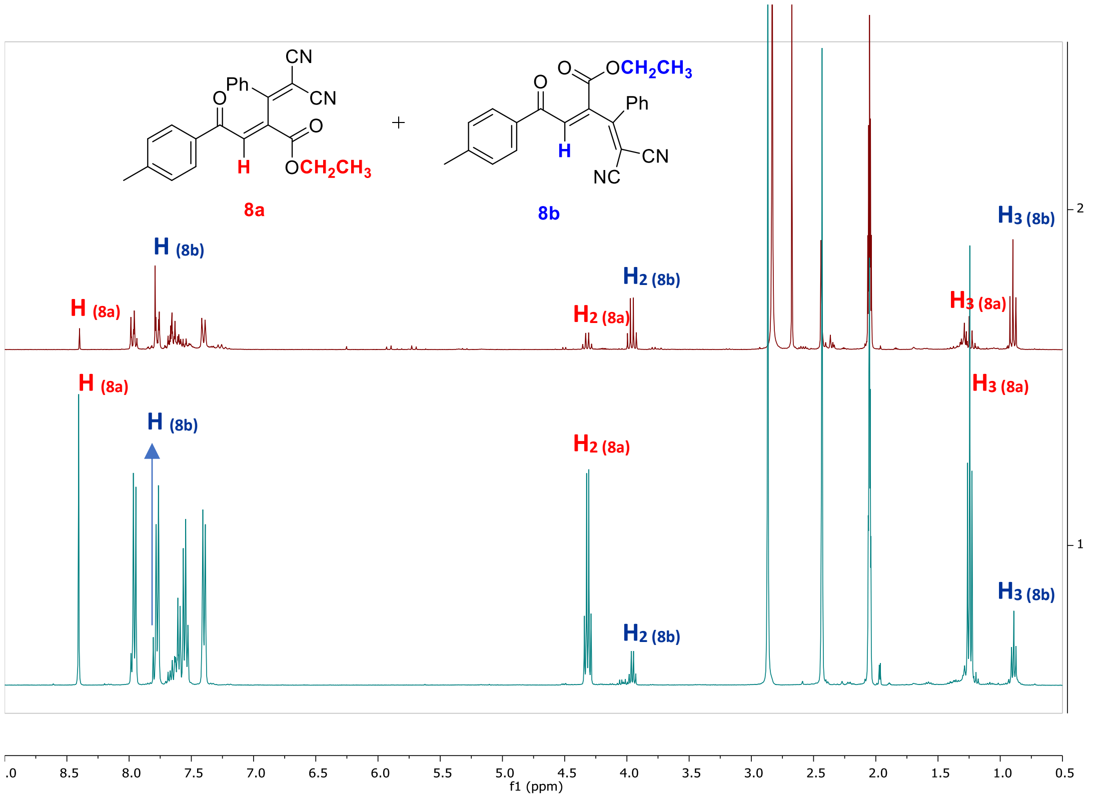

Computational Details

All calculations were performed using the Gaussian 16 software package,[1] without symmetry constraints. The optimized geometries were obtained employing the M06-2X functional[2] with a standard 6-31+G(d,p)[3] basis set as implemented on Gaussian 16. Transition state optimizations were performed with the Synchronous Transit-Guided Quasi-Newton Method (STQN) developed by Schlegel *et al.*[4] Frequency calculations were performed to confirm the nature of the stationary points, yielding one imaginary frequency for the transition states and none for the minima. Each transition state was further confirmed by following its

vibrational mode downhill on both sides and obtaining the minima presented on the energy profile. A Natural Population Analysis (NPA)[5] and the resulting Wiberg indices[6] were used to study the electronic structure and bonding of the optimized species, and calculate as implemented on Gaussian 16.

The electronic energies (E_{b1}) obtained at the M06-2X/6-31+G(d,p) level of theory were converted to free energy at 298.15 K and 1 atm (G_{b1}). Single point energy calculations were performed using the M06-2X functional and a standard 6-311++G(d,p) basis set, taking into account the solvent effects (toluene) using the Polarizable Continuum Model (PCM) initially devised by Tomasi and coworkers[7] as implemented on Gaussian 09, with radii and non-electrostatic terms for Truhlar and coworkers' SMD solvation model.[8]

The free energy values presented along the text (G_{b2}) were derived from the electronic energy values obtained at the M06-2X/6-311++G(d,p)// M06-2X/6-31+G(d,p) level, including solvent effects (E_{b2}), according to the following expression: $G_{b2} = E_{b2} + G_{b1} - E_{b1}$.

Absolute Calculation Energies and Free energies

Geometry	E_{b1} a)	G_{b1} b)	E_{b2} c)	G_{b2} d)	IF e)
**bzs**	-754.016384	-753.845806	-754.155754	-753.985176	-
**sc^3**	-753.949447	-753.783165	-754.087701	-753.921419	-
**sc^1**	-753.978837	-753.808860	-754.117126	-753.947149	-
**dn**	-799.110735	-798.947022	-799.310969	-799.147255	-
**$[sc^1dn]_1$**	-1553.109890	-1552.752107	-1553.443284	-1553.085501	-
**$ts1_{trans}$**	-1553.099070	-1552.741422	-1553.433601	-1553.075953	-188.75
**cp_{trans}**	-1553.191728	-1552.829067	-1553.524518	-1553.161857	-
**$ts2_{trans}$**	-1553.162955	-1552.797431	-1553.504442	-1553.138918	-27.92
**prod**	-1553.213006	-1552.848490	-1553.545853	-1553.181337	-
**$[sc^1dn]_1$**	-1553.104506	-1552.750545	-1553.439369	-1553.085408	-
**$ts1_{cis}$**	-1553.099798	-1552.741387	-1553.435202	-1553.076791	-168.14
**cp_{cis}**	-1553.190290	-1552.825973	-1553.522906	-1553.158588	-
**$ts2_{cis}$**	-1553.160344	-1552.796824	-1553.504050	-1553.140530	-18.03
**int_1**	-1553.163706	-1552.801401	-1553.509513	-1553.147208	-
**$int_{1'}$**	-1553.164902	-1552.801878	-1553.507592	-1553.144568	-
**$ts3_{cis}$**	-1553.156194	-1552.791108	-1553.499517	-1553.134431	-27.24

a) Electronic energy calculated at M06-2X/6-31+G(d,p) (gas phase); b) Free energy values calculated at M06-2X/6-31+G(d,p) in gas phase; c) Electronic energy calculated at M06-2X/6-311++G(d,p) including solvent effects (toluene); d) Free energy values at M06-2X/6-311++G(d,p), determined according to $G_{b2} = E_{b2} + G_{b1} - E_{b1}$; e) Calculated Imaginary Frequencies for optimized geometries at M06-2X/6-31+G(d,p)

NMR spectra of novel compounds:

^{1}H NMR (300 MHz, $CDCl_3$) of **2l**:

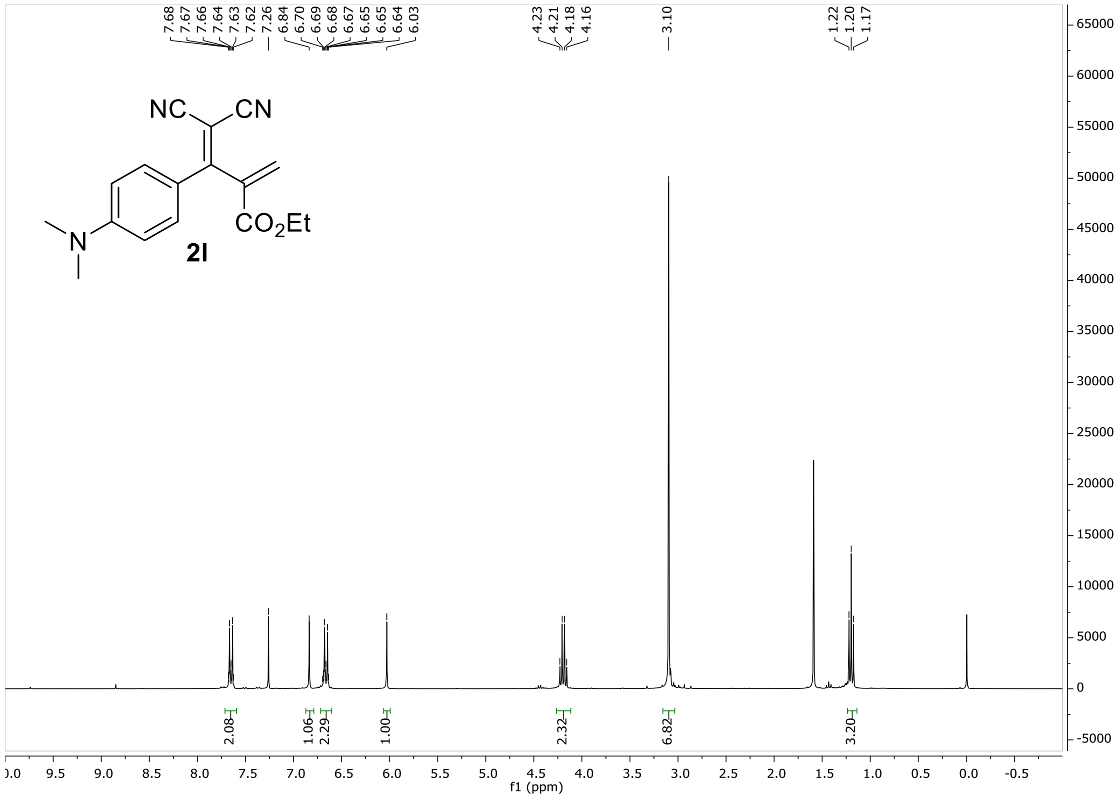

^{13}C{1H} NMR (75 MHz, $CDCl_3$) of **2l**:

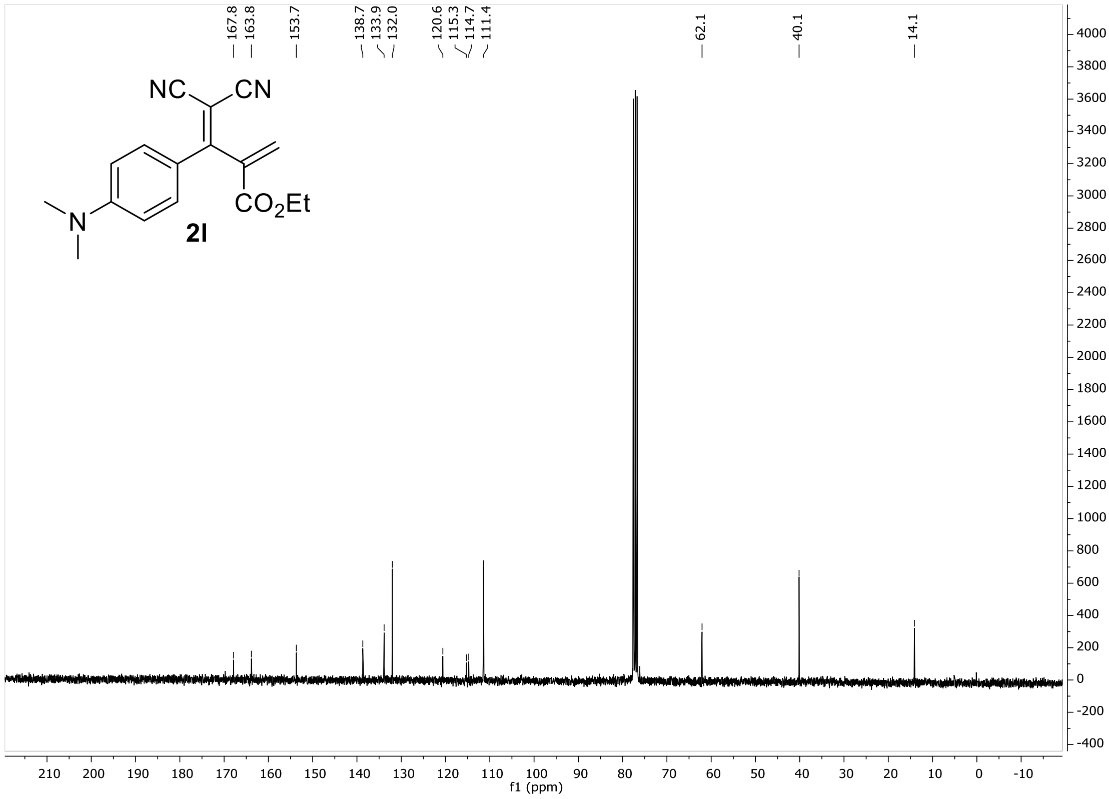

^{1}H NMR (300 MHz, $CDCl_3$) of **9b**:

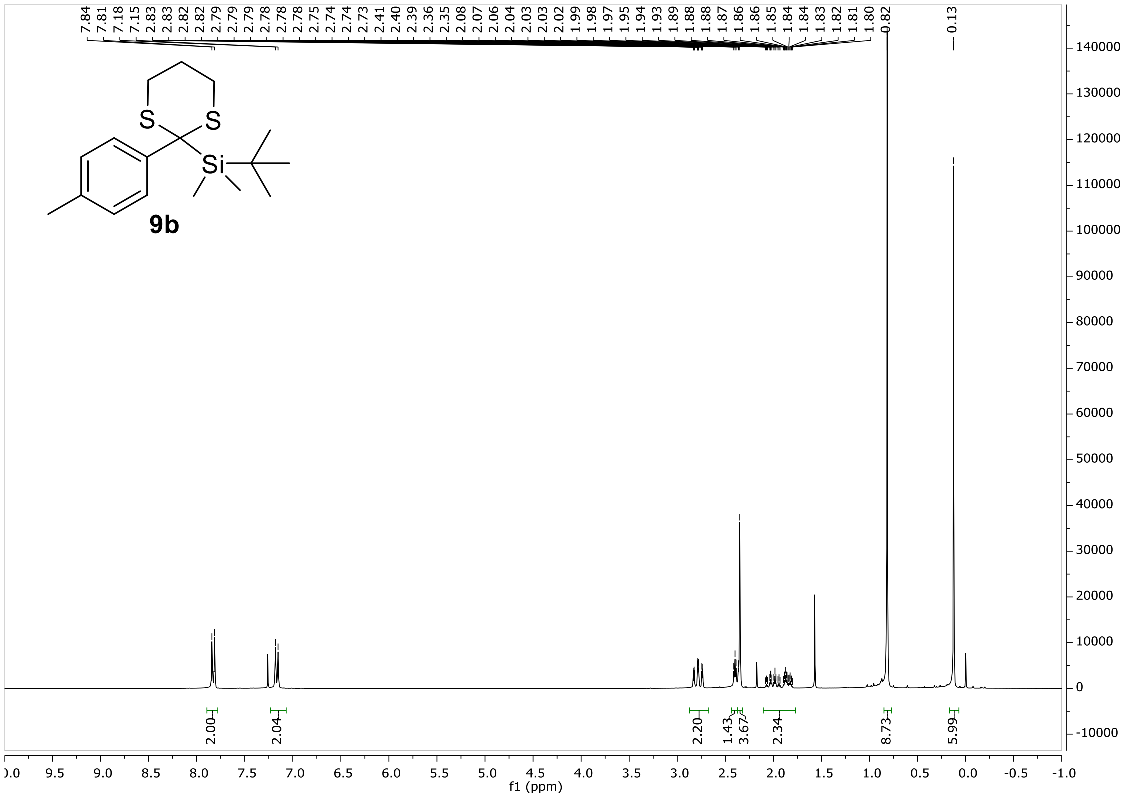

^{13}C{1H} NMR (75 MHz, $CDCl_3$) of **9b**:

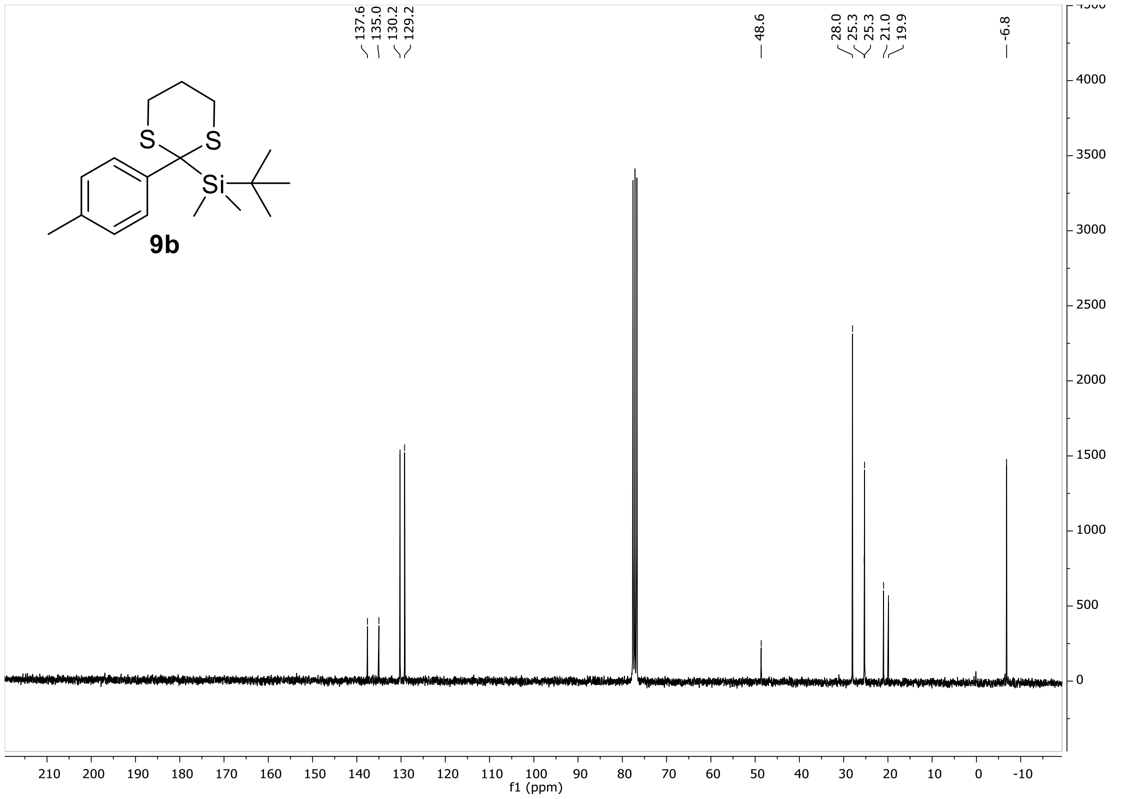

^{1}H NMR (300 MHz, $CDCl_3$) of **9e**:

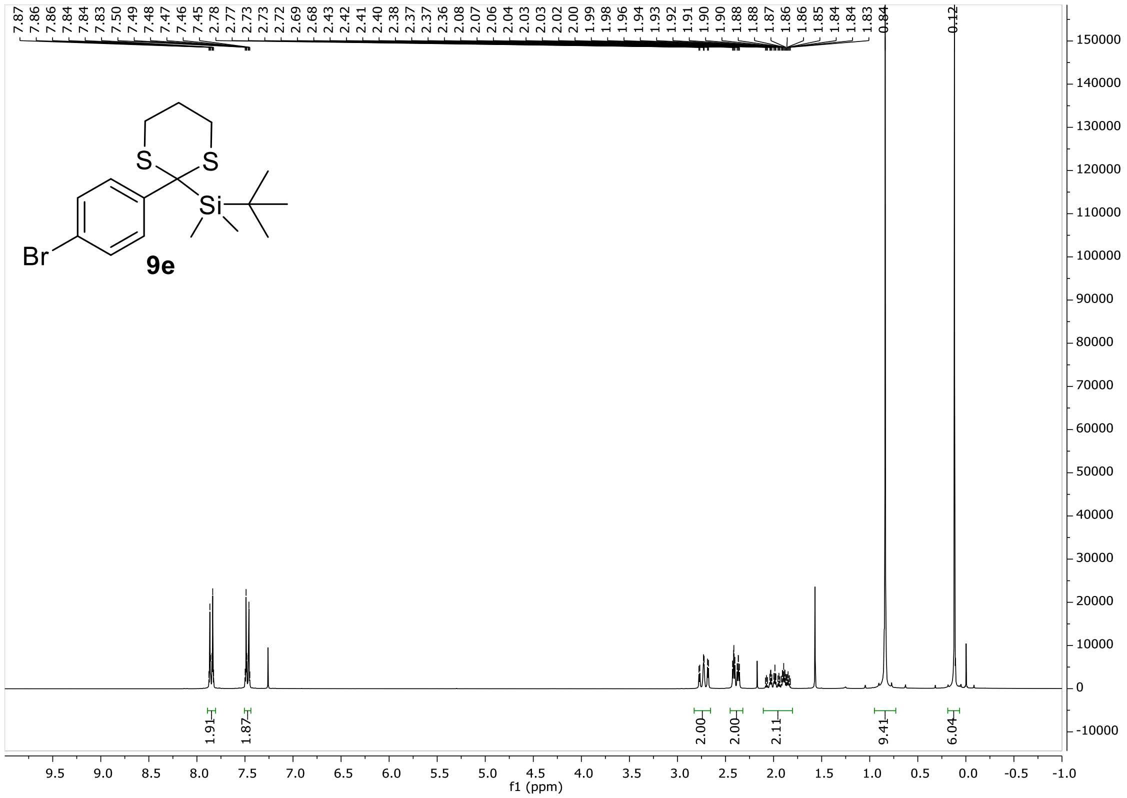

^{13}C{1H} NMR (75 MHz, $CDCl_3$) of **9e**:

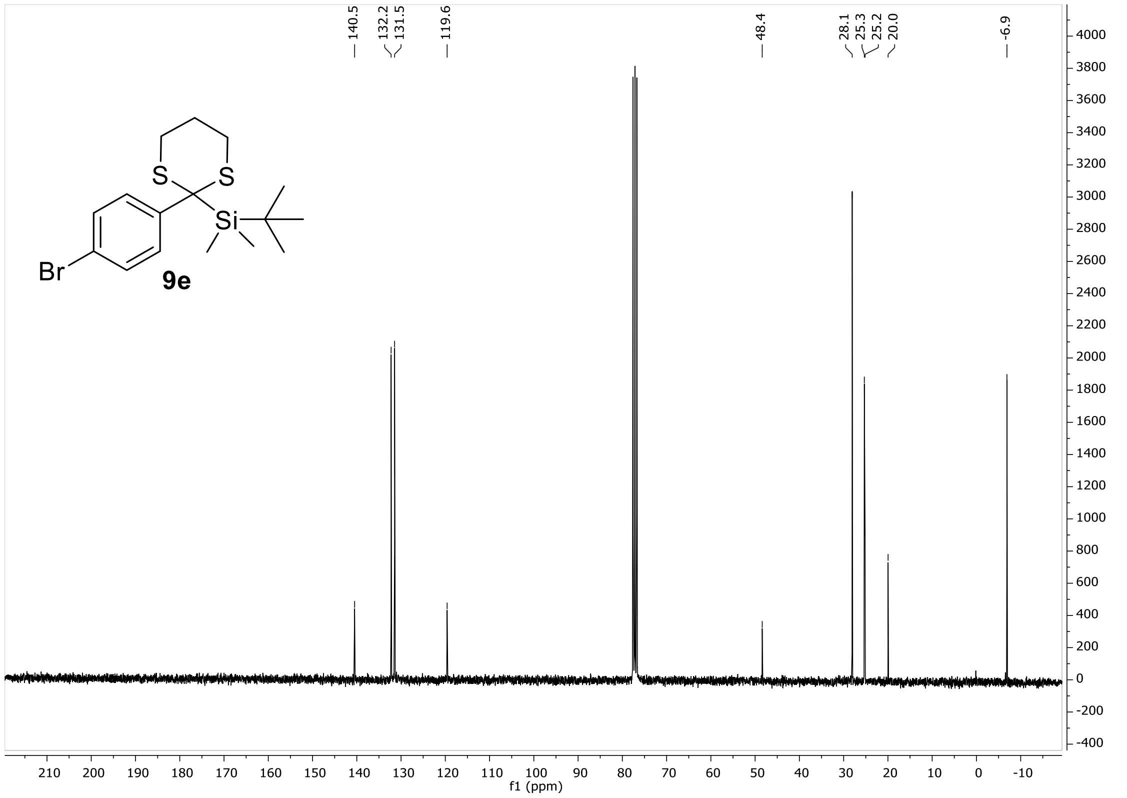

^{1}H NMR (300 MHz, $CDCl_3$) of **9f**:

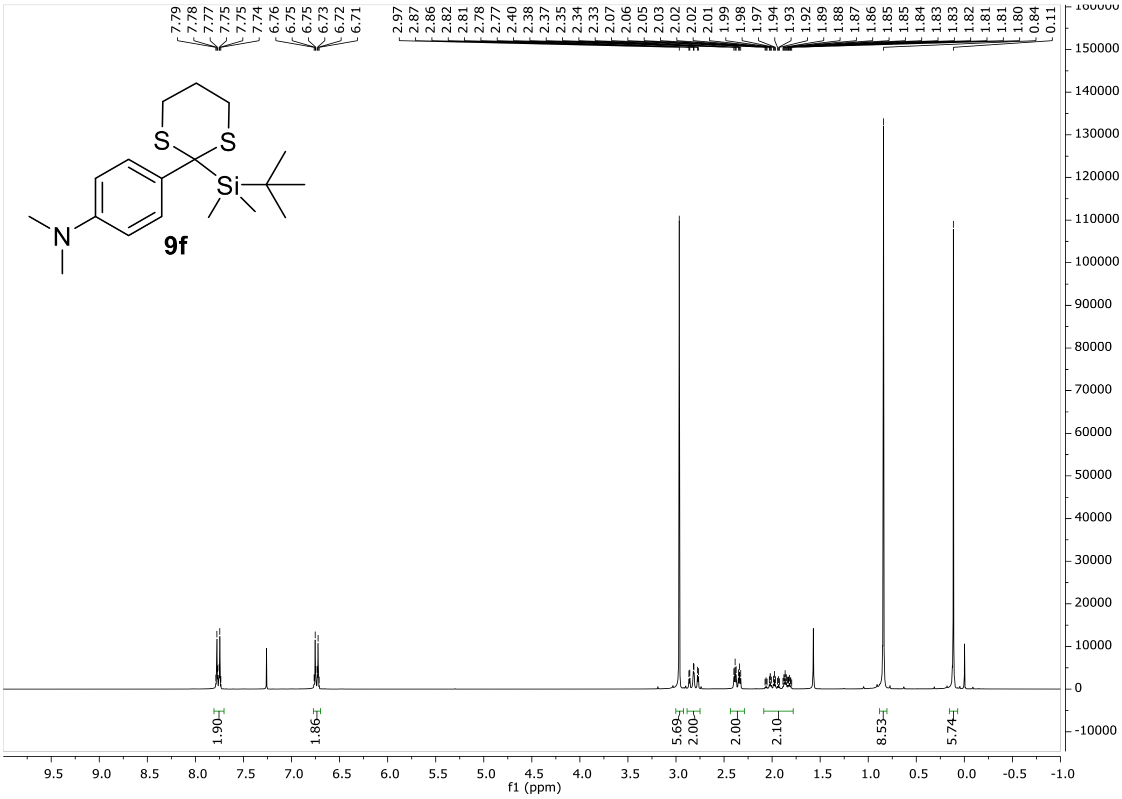

^{13}C{1H} NMR (75 MHz, $CDCl_3$) of **9f**:

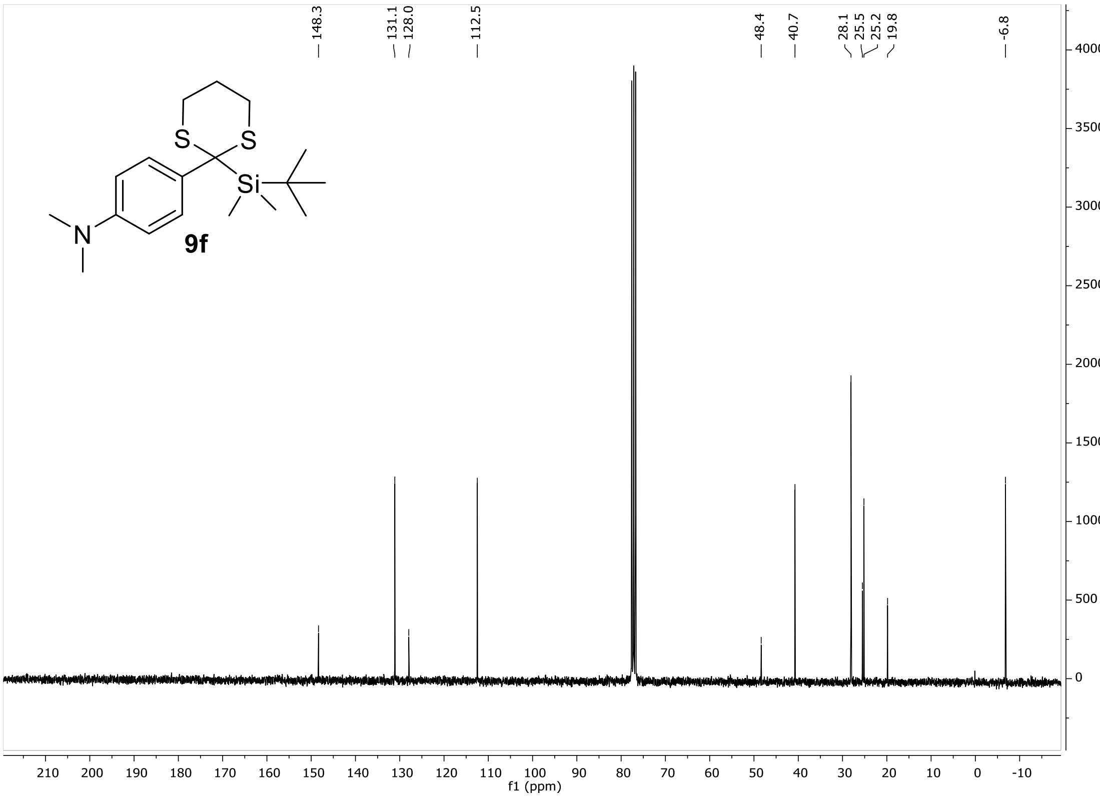

^{1}H NMR (300 MHz, $CDCl_3$) of **9n**:

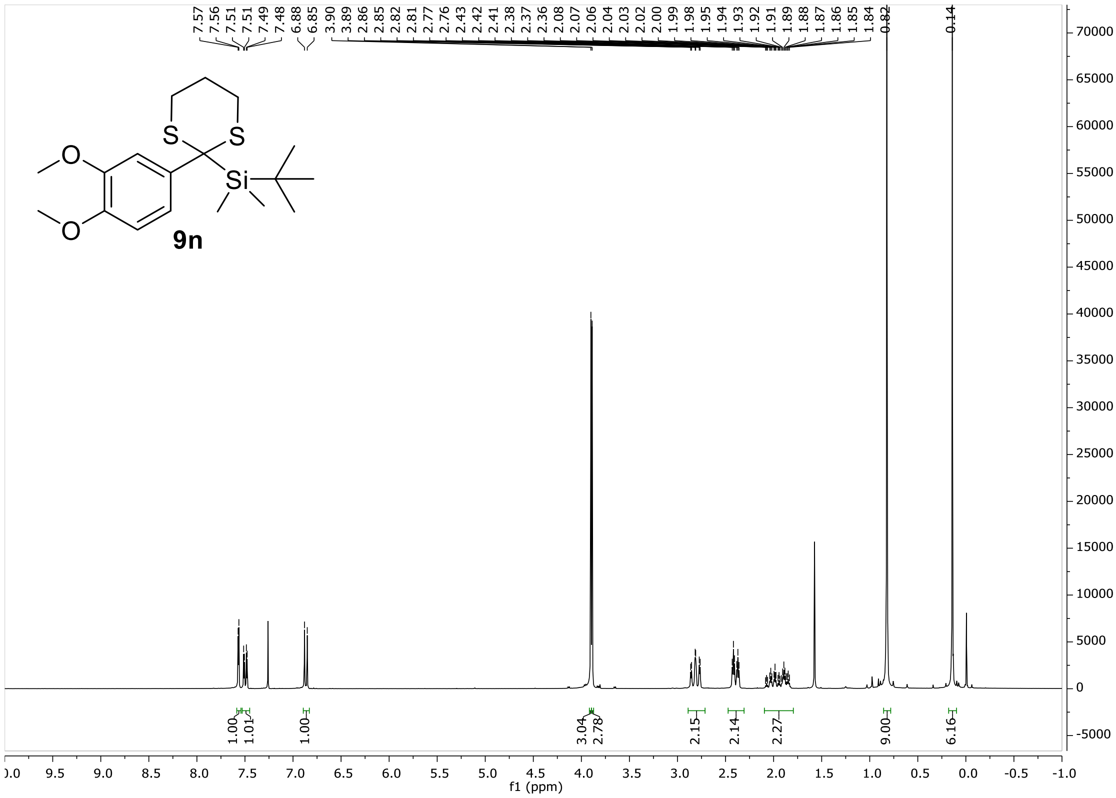

^{13}C{1H} NMR (75 MHz, $CDCl_3$) of **9n**:

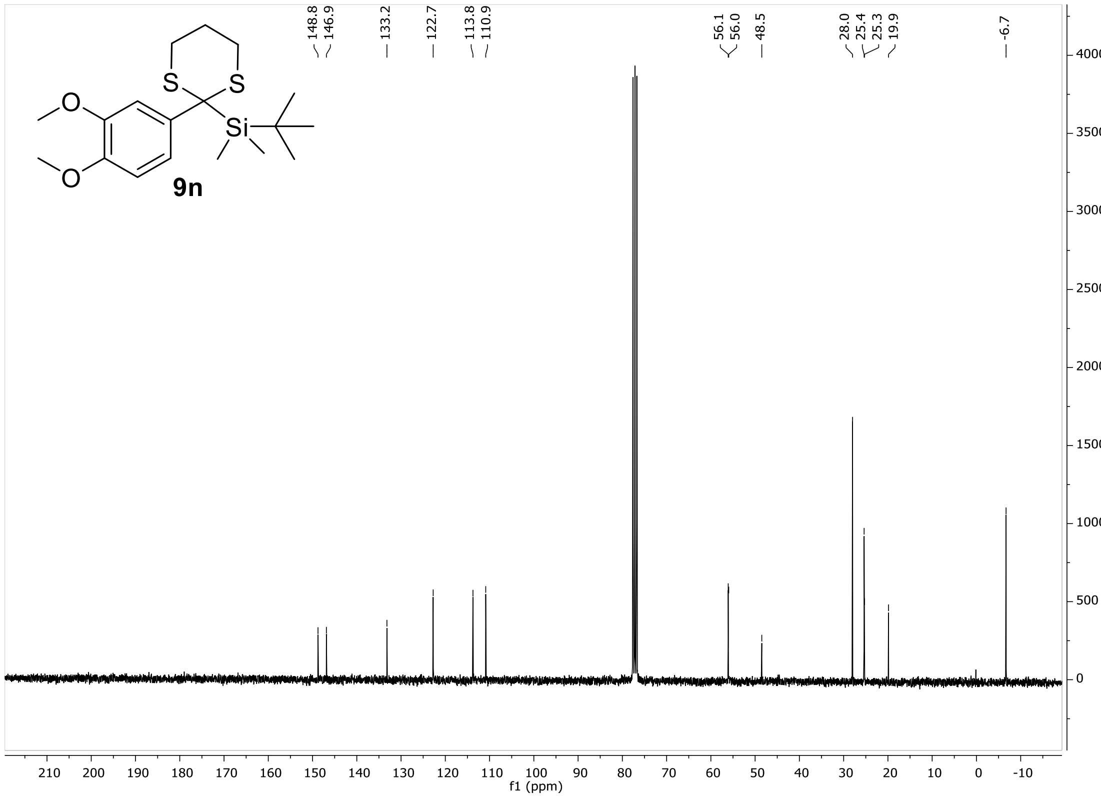

^{1}H NMR (300 MHz, $CDCl_3$) of **1e**:

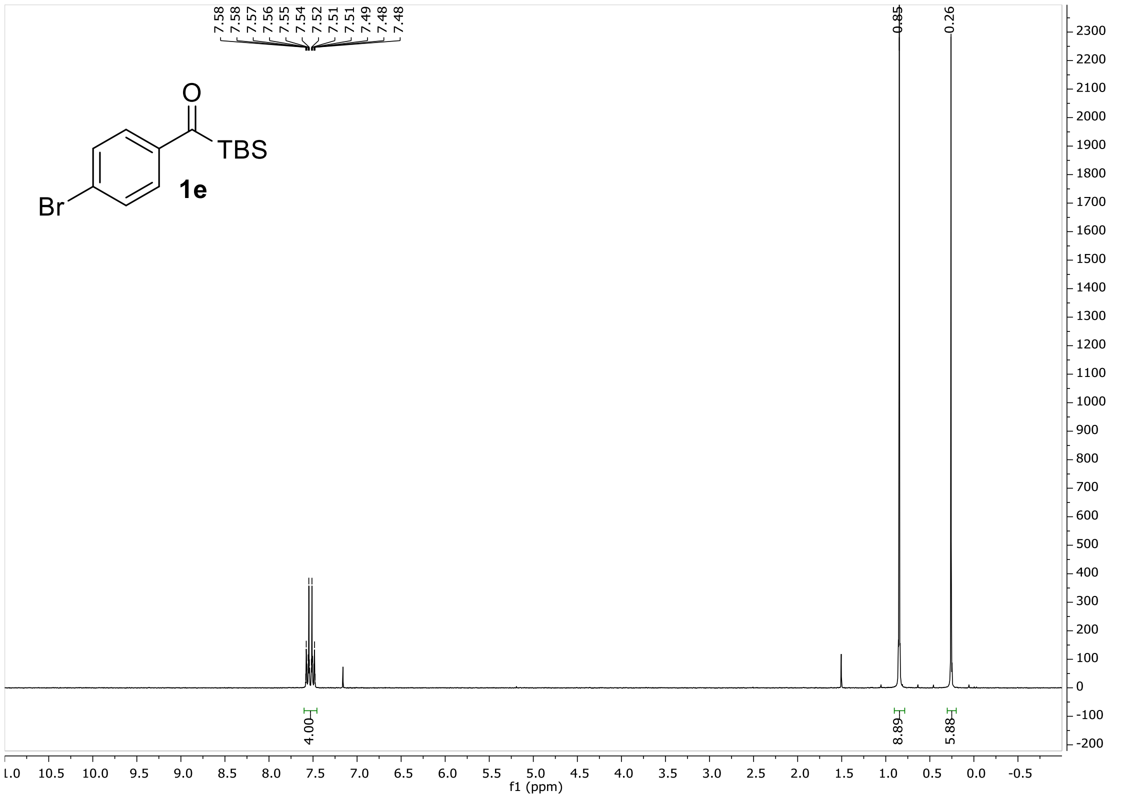

^{13}C{1H} NMR (100 MHz, $CDCl_3$) of **1e**:

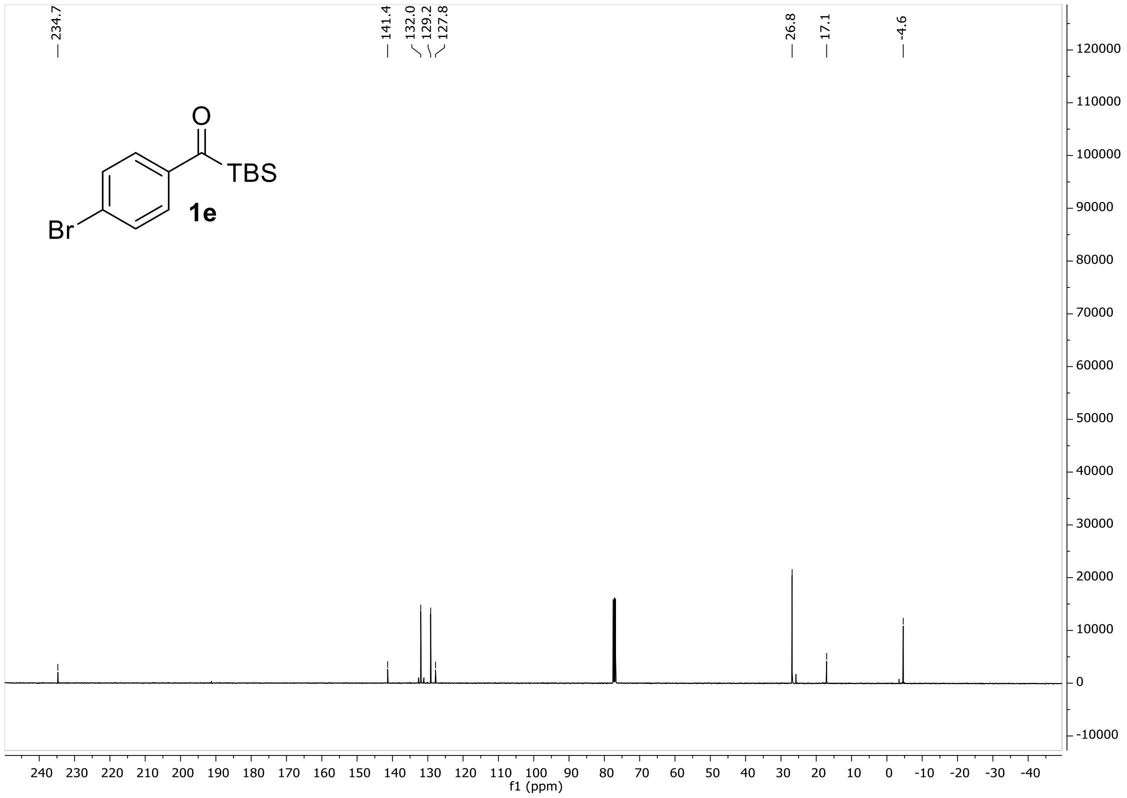

^{1}H NMR (300 MHz, $CDCl_3$) of **1f**:

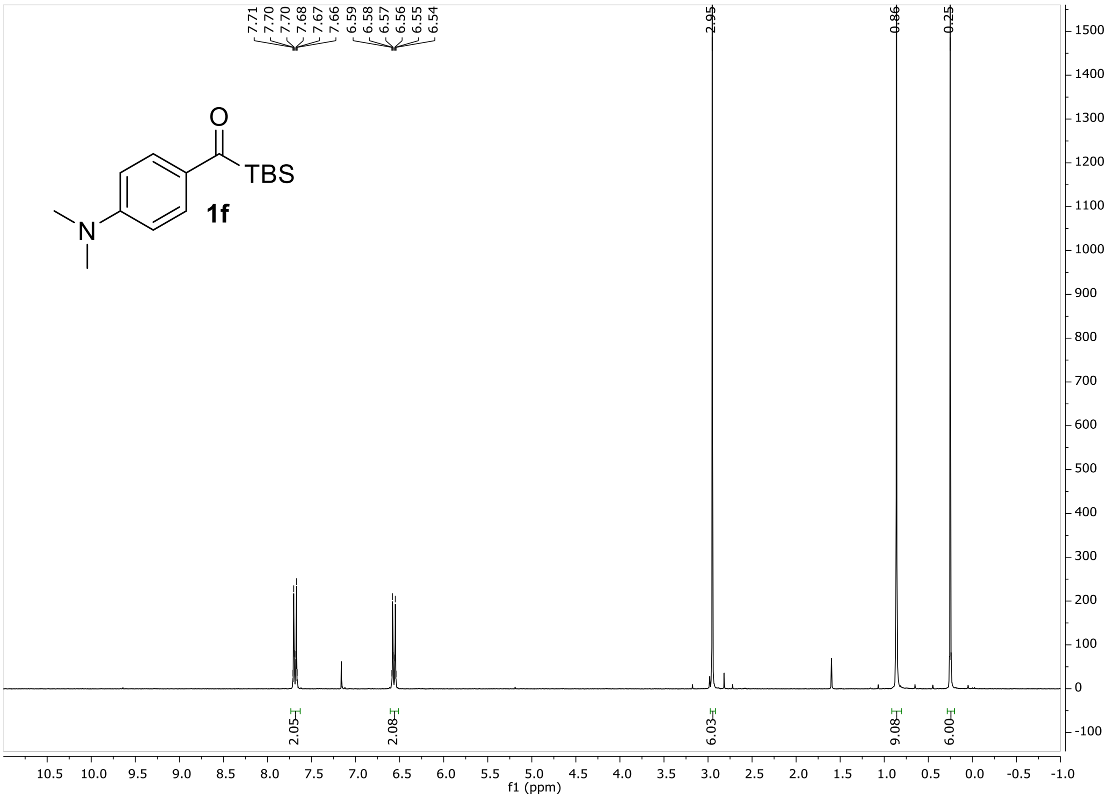

^{13}C{1H} NMR (100 MHz, $CDCl_3$) of **1f**:

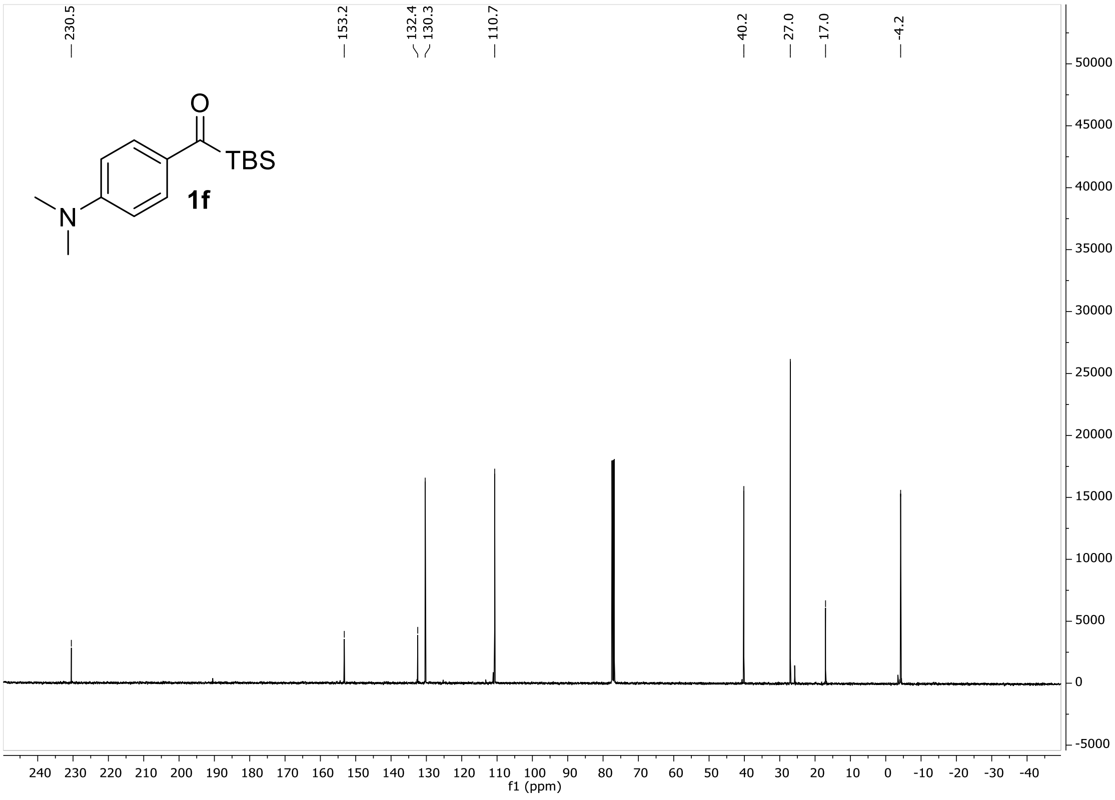

^{1}H NMR (300 MHz, $CDCl_3$) of **1n**:

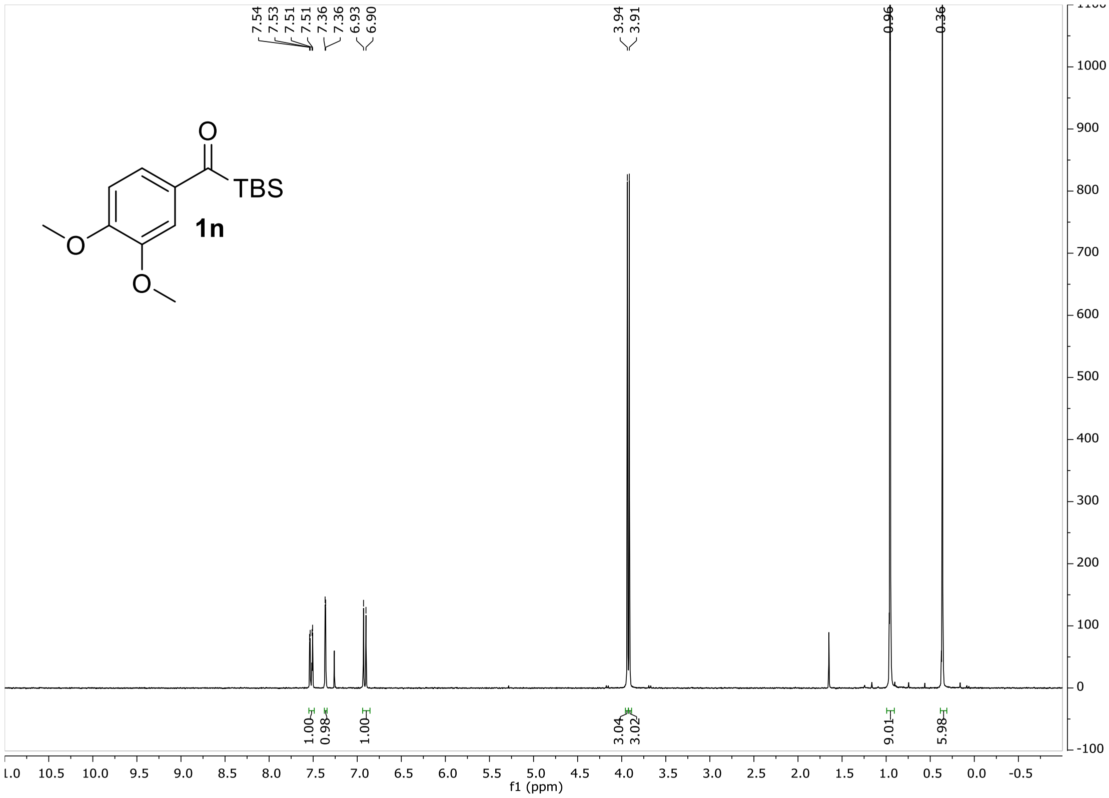

^{13}C{1H} NMR (100 MHz, $CDCl_3$) of **1n**:

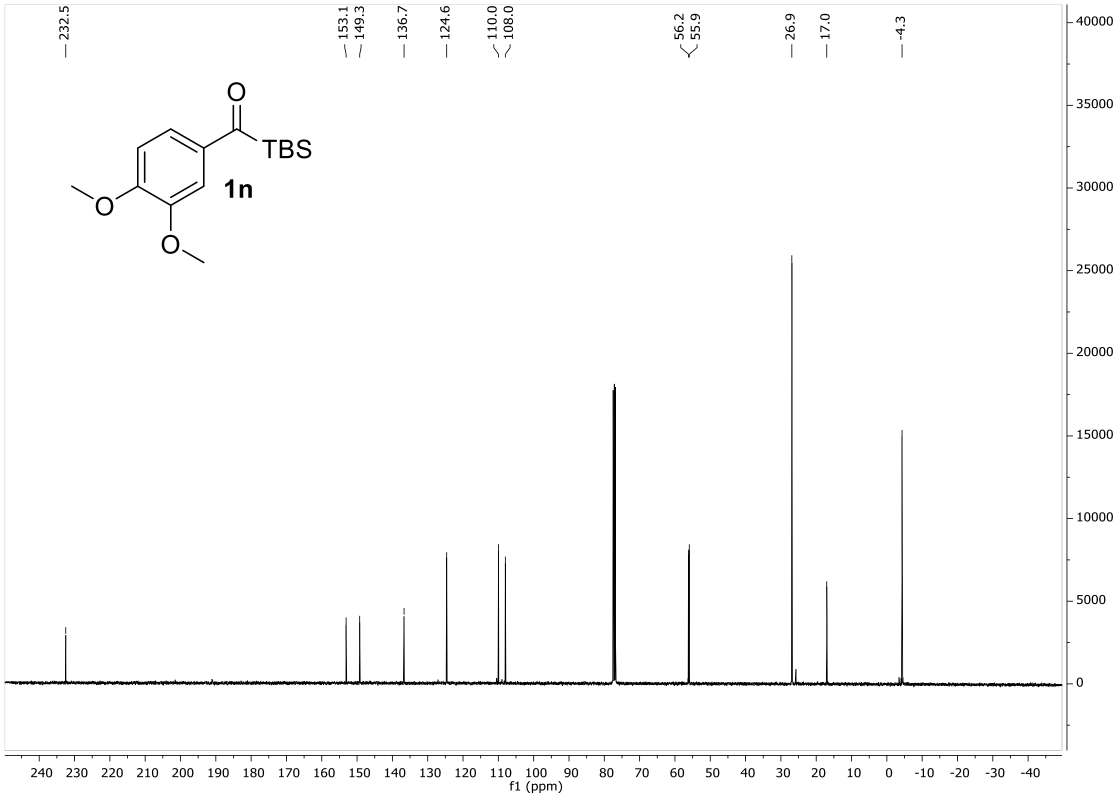

^{1}H NMR (300 MHz, $CDCl_3$) of **3a**:

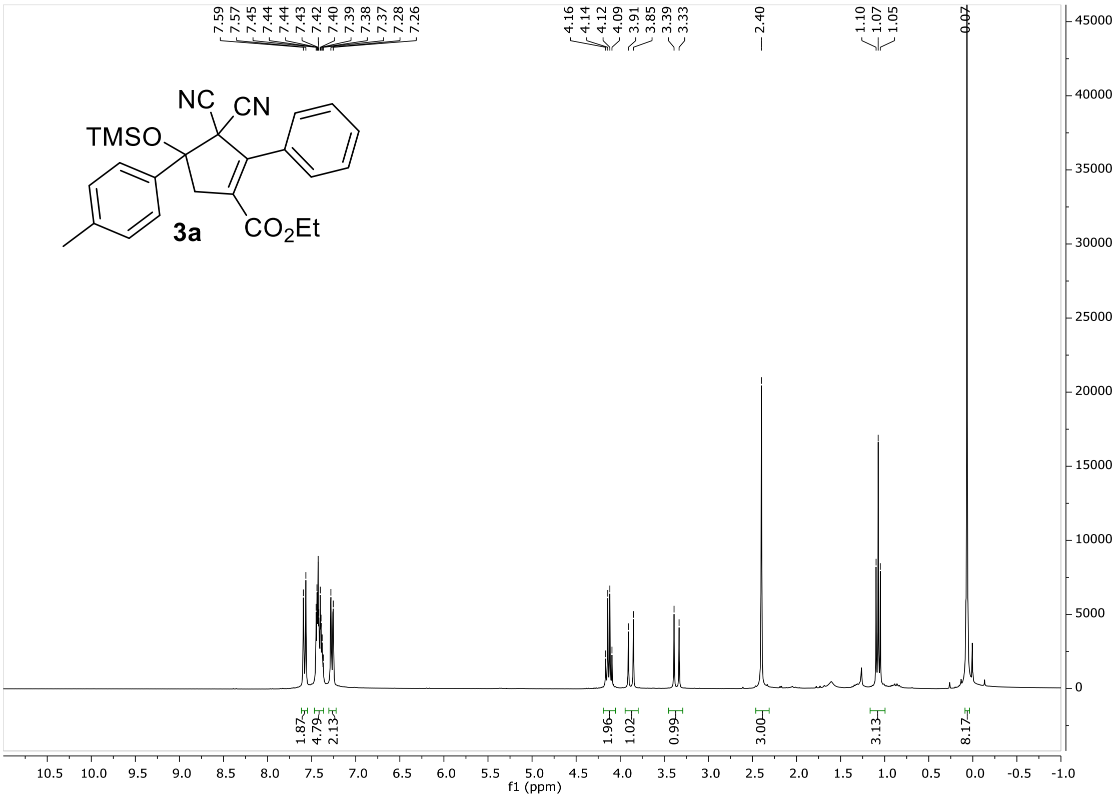

^{13}C{1H} NMR (75 MHz, $CDCl_3$) of **3a**:

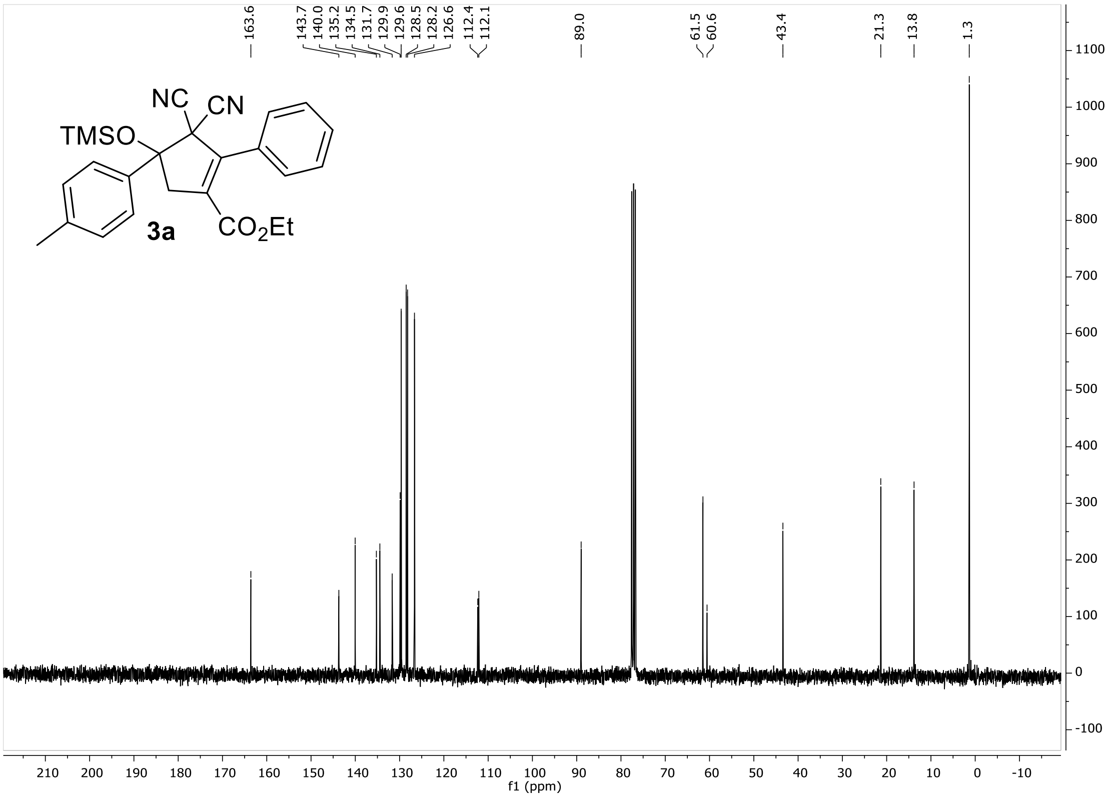

^{1}H NMR (300 MHz, $CDCl_3$) of **3b**:

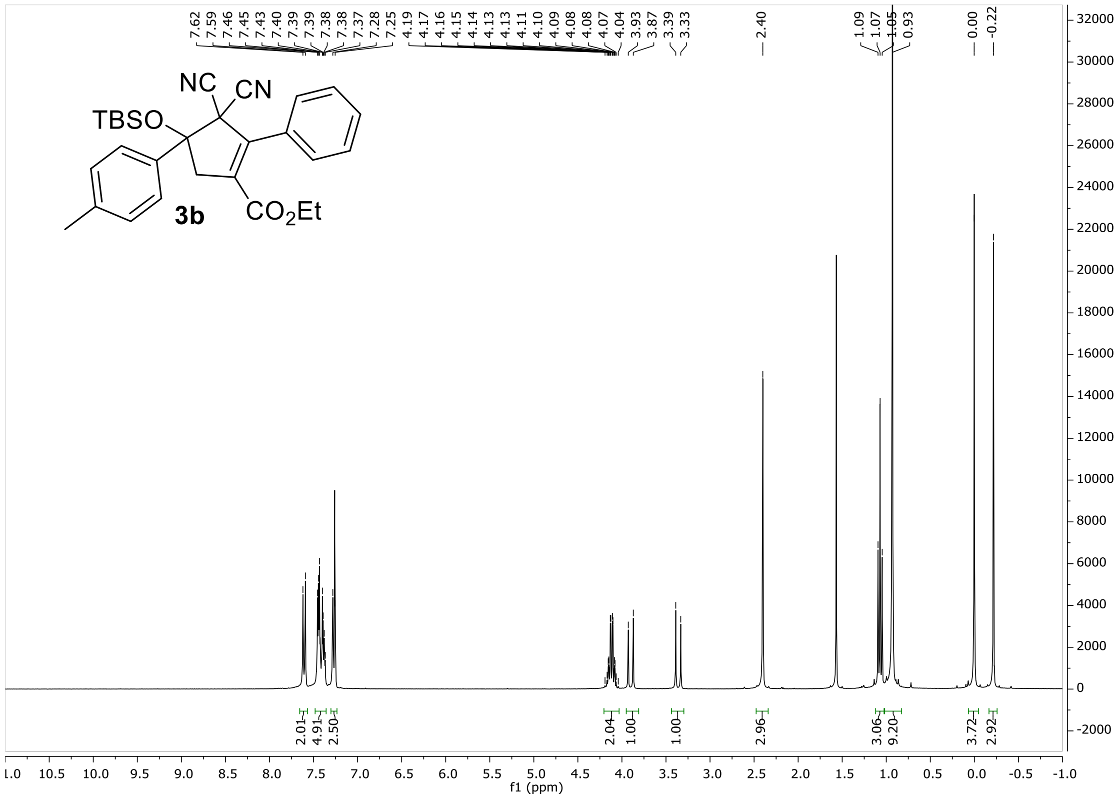

^{13}C{1H} NMR (75 MHz, $CDCl_3$) of **3b**:

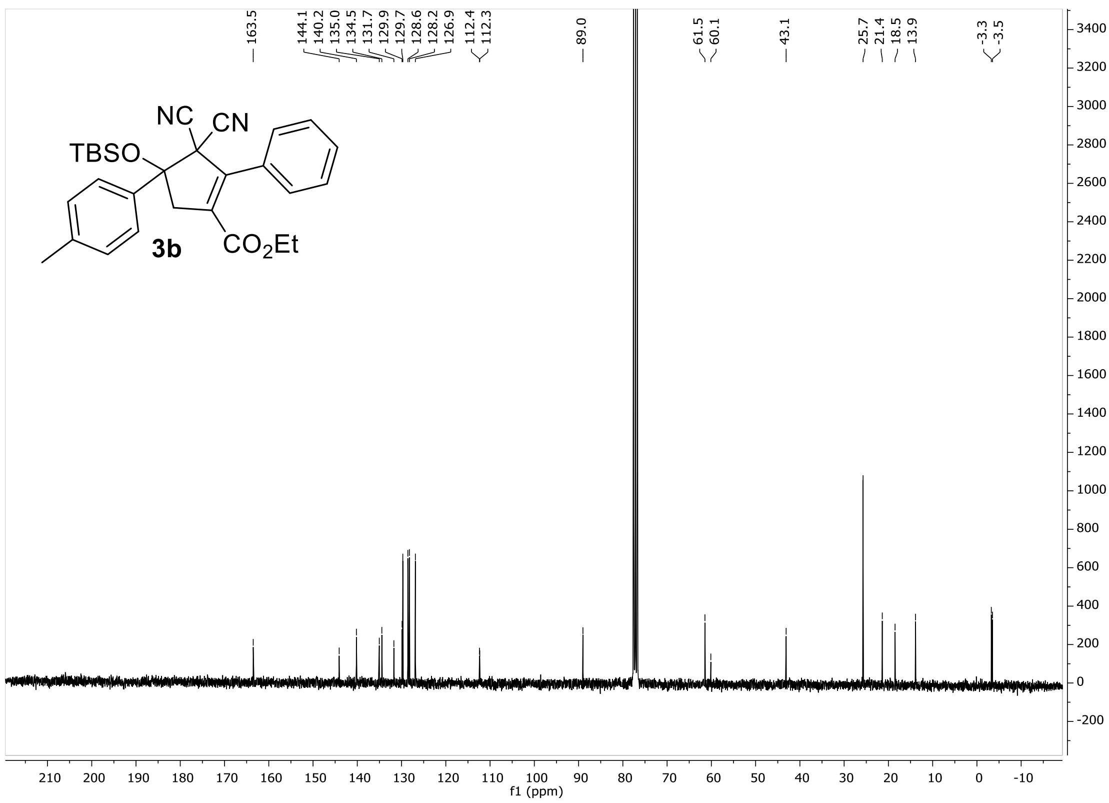

^{1}H NMR (300 MHz, $CDCl_3$) of **3c**:

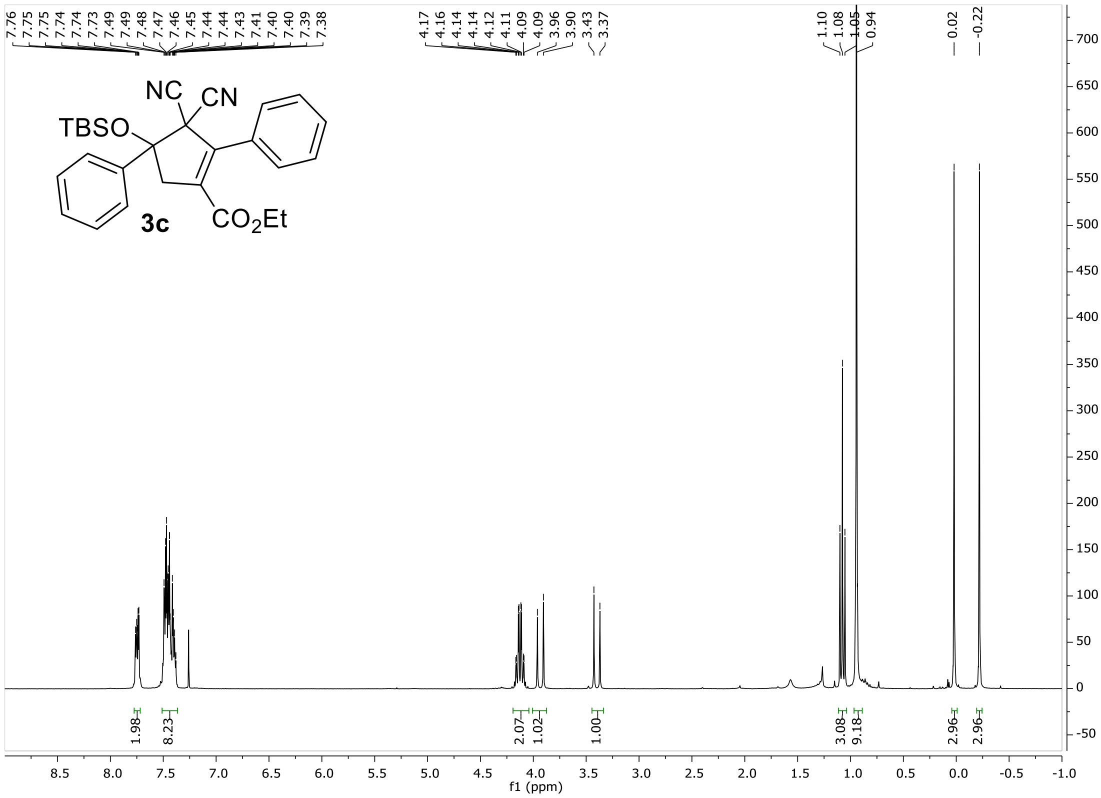

^{13}C{1H} NMR (75 MHz, $CDCl_3$) of **3c**:

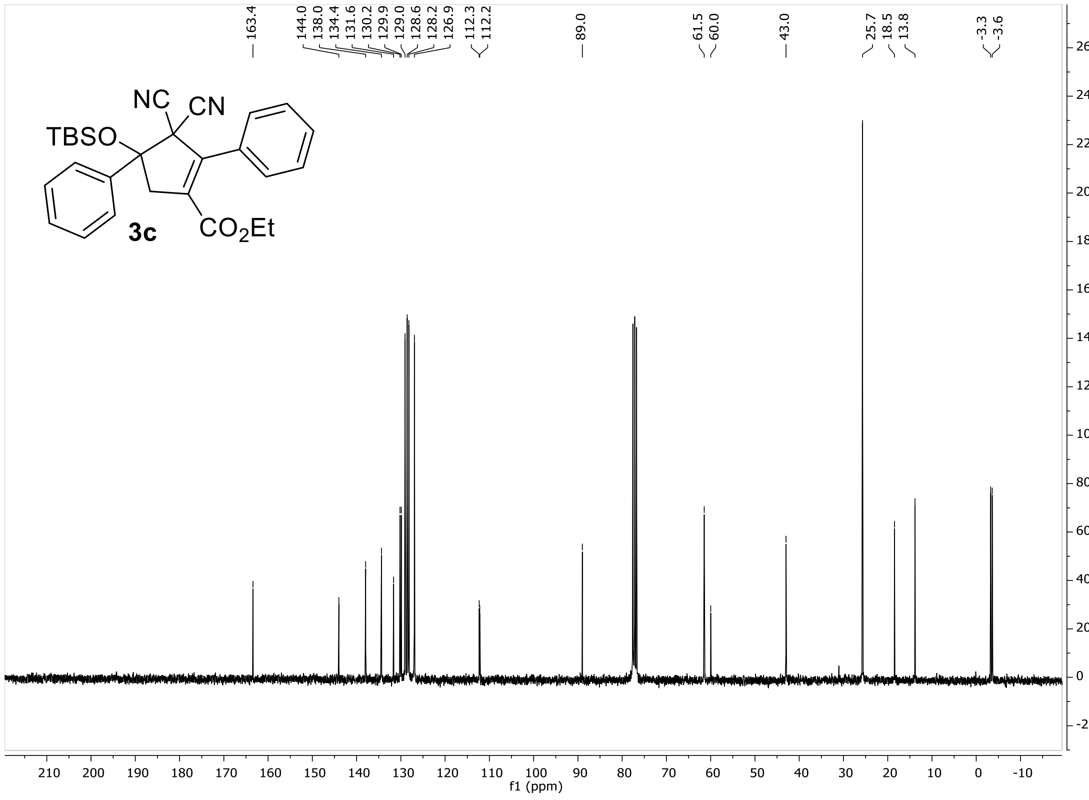

^{1}**H NMR** (400 MHz, $CDCl_3$) of **3d**:

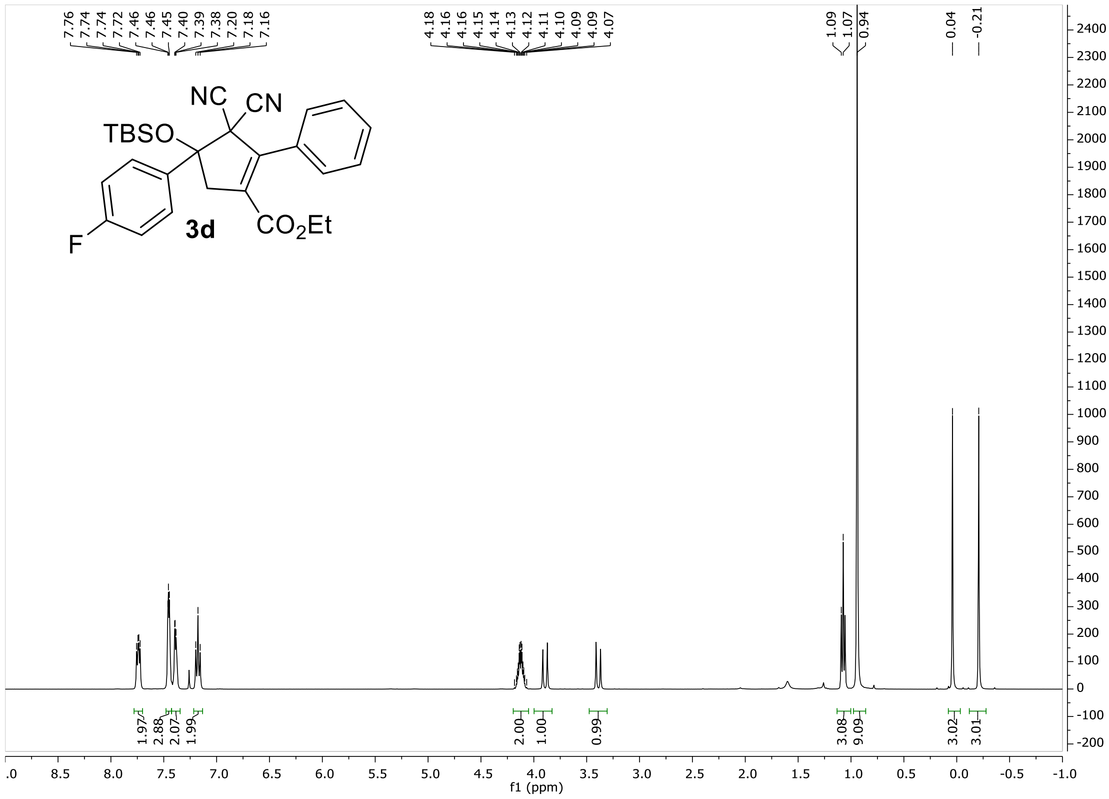

^{13}**C{1H} NMR** (75 MHz, $CDCl_3$) of **3d**:

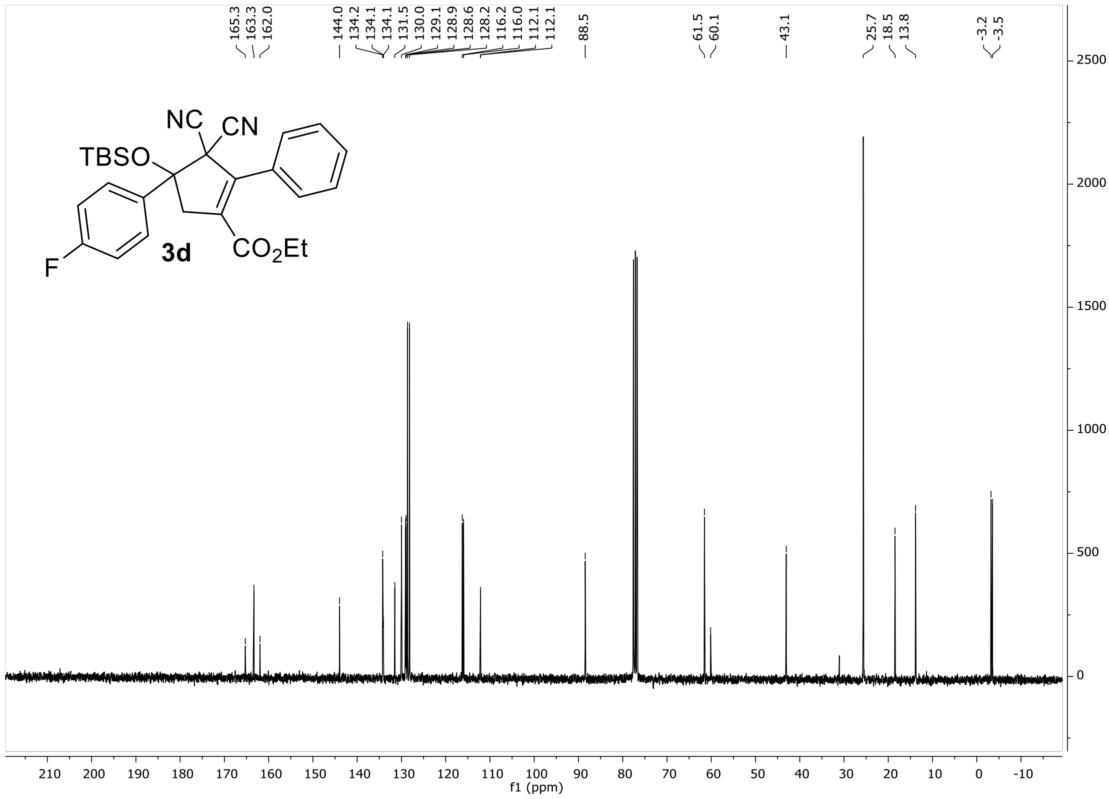

^{19}F{1H} NMR (376 MHz, $CDCl_3$) of **3d**:

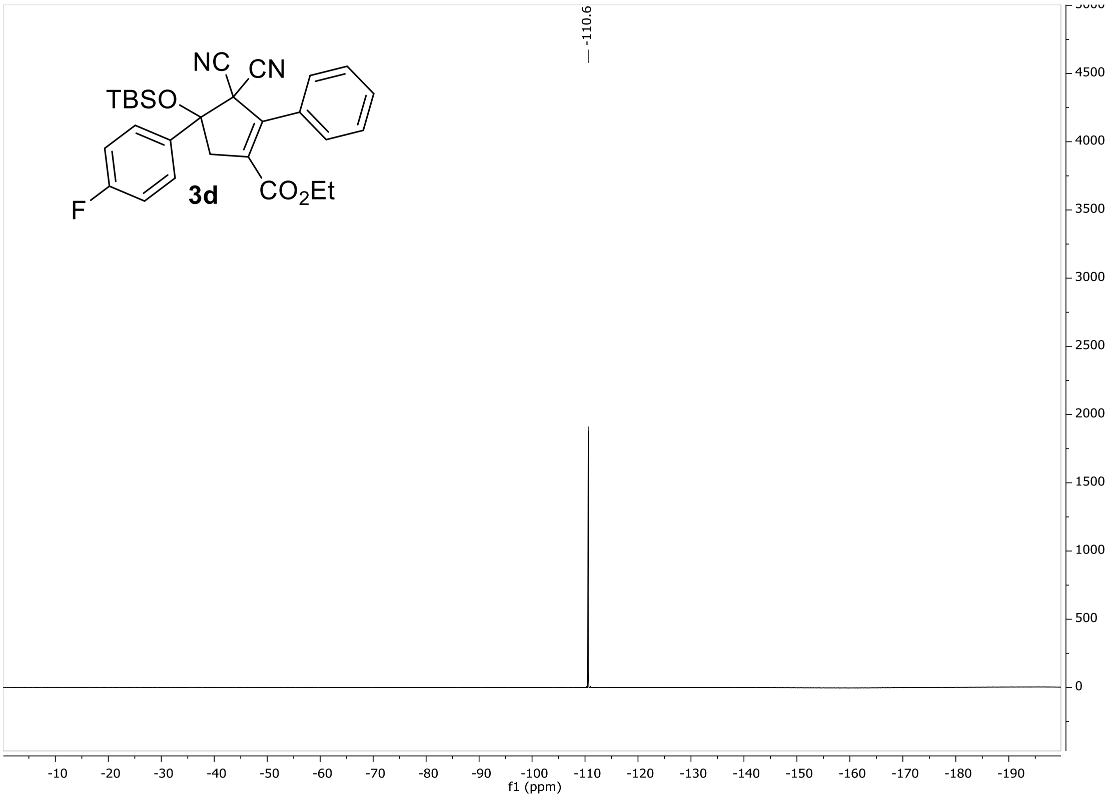

^{1}H NMR (300 MHz, $CDCl_3$) of **3e**:

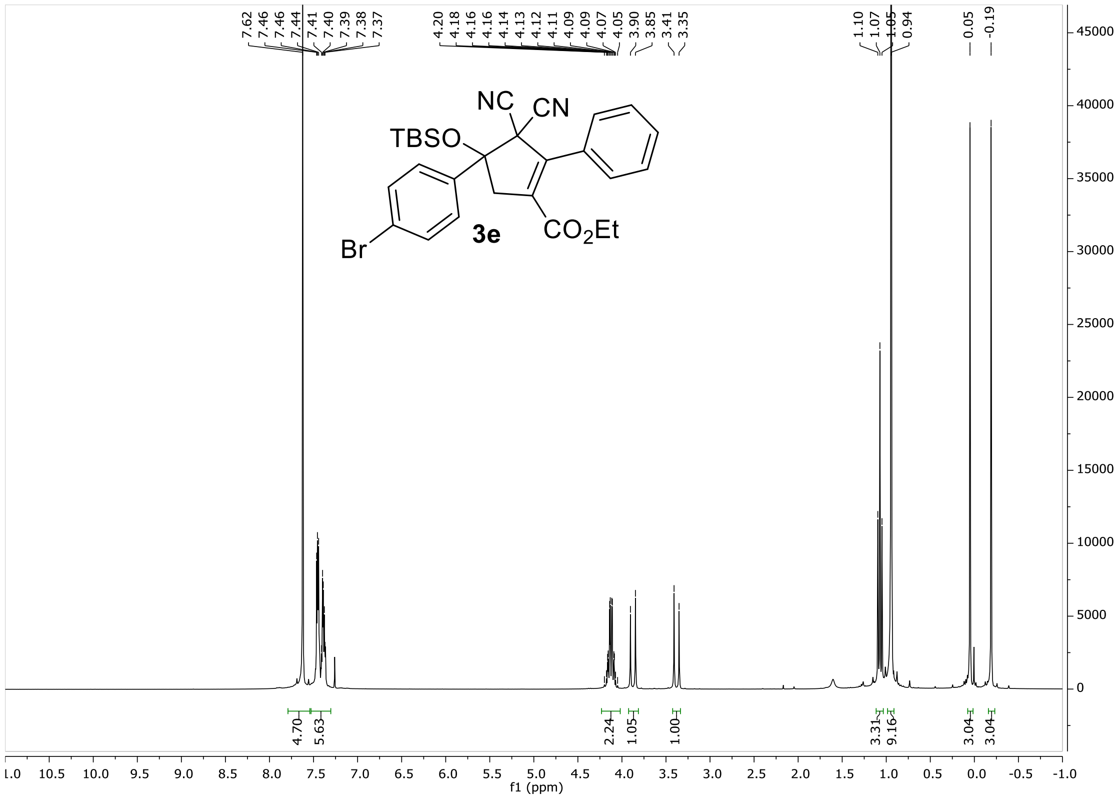

^{13}C{1H} NMR (75 MHz, $CDCl_3$) of **3e**:

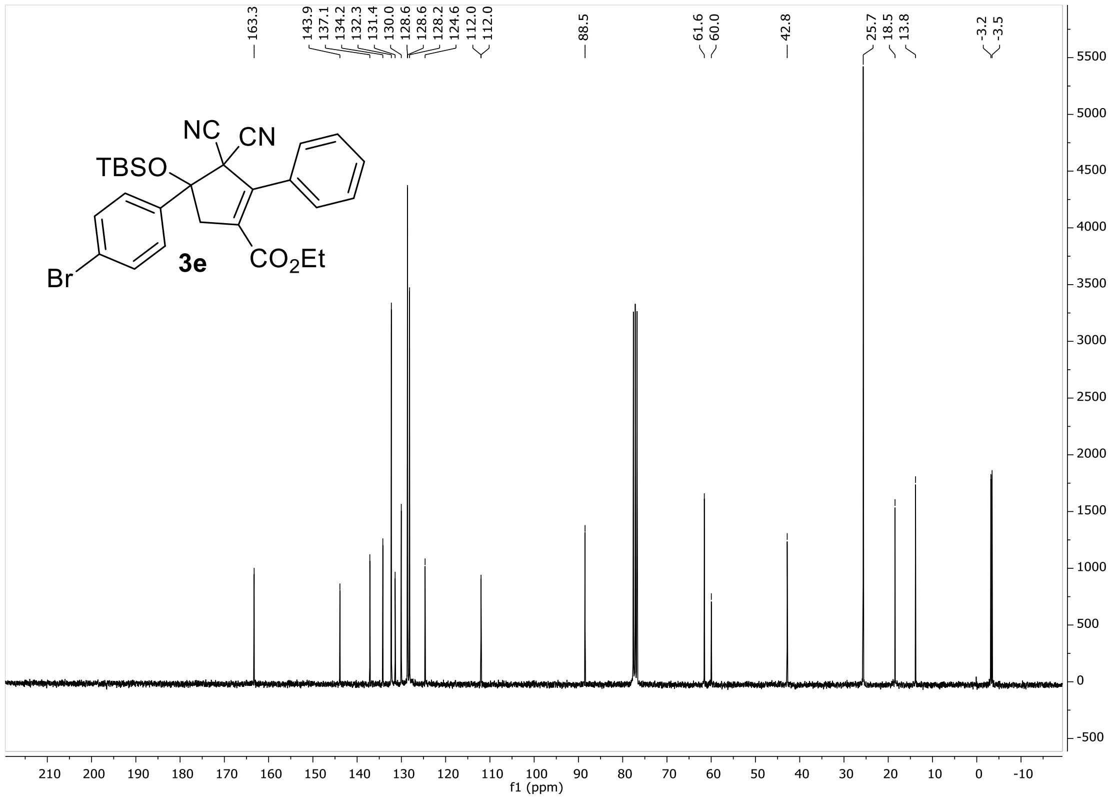

^{1}H NMR (300 MHz, $CDCl_3$) of **3f**:

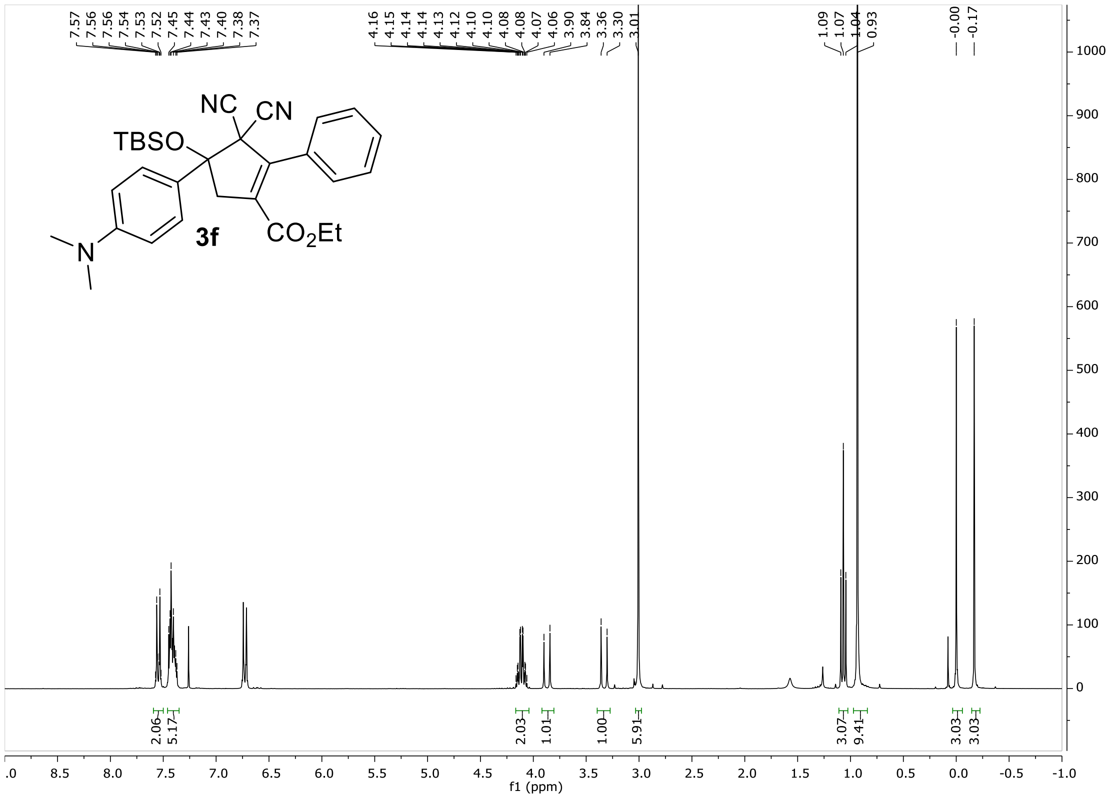

^{13}C{1H} NMR (75 MHz, $CDCl_3$) of **3f**:

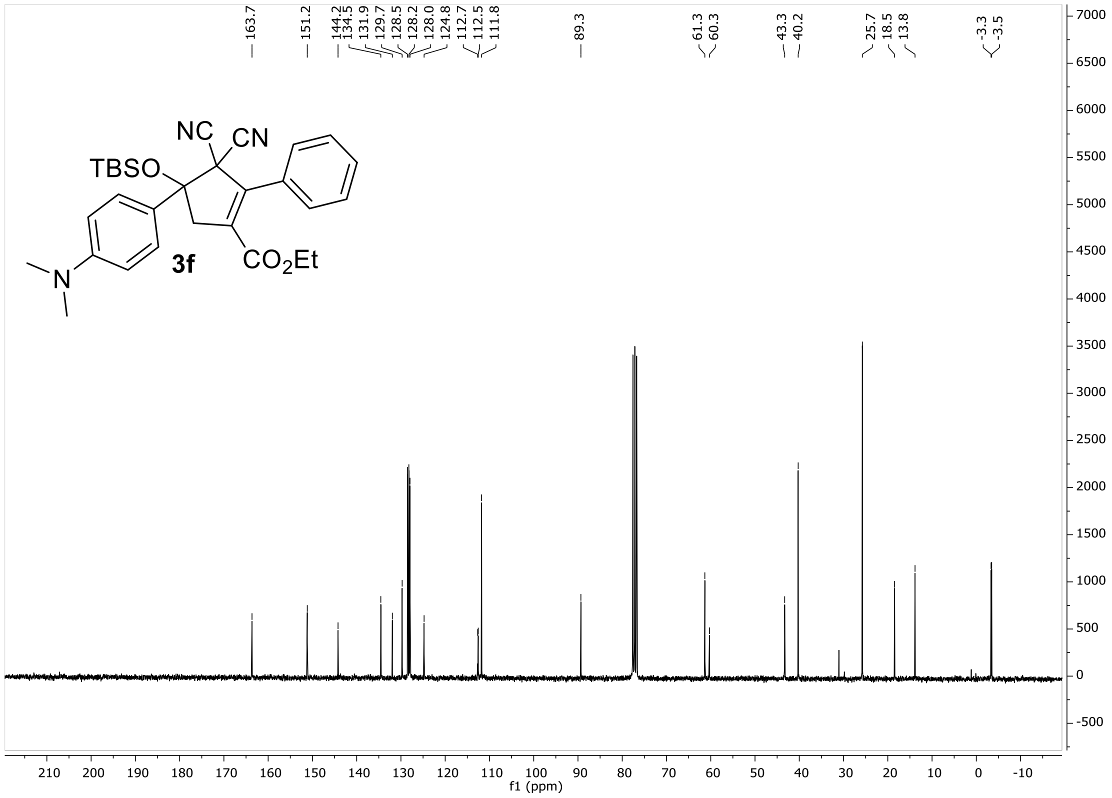

^{1}H NMR (300 MHz, $CDCl_3$) of **3h**:

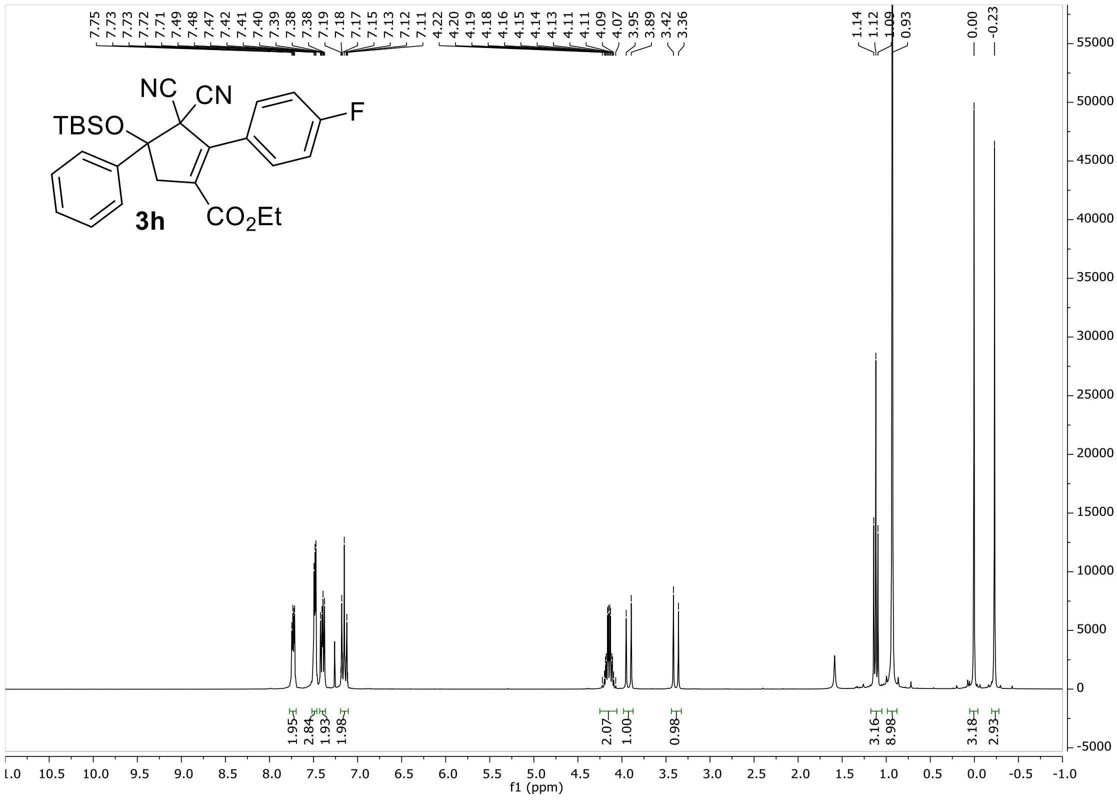

^{13}C{1H} NMR (75 MHz, $CDCl_3$) of **3h**:

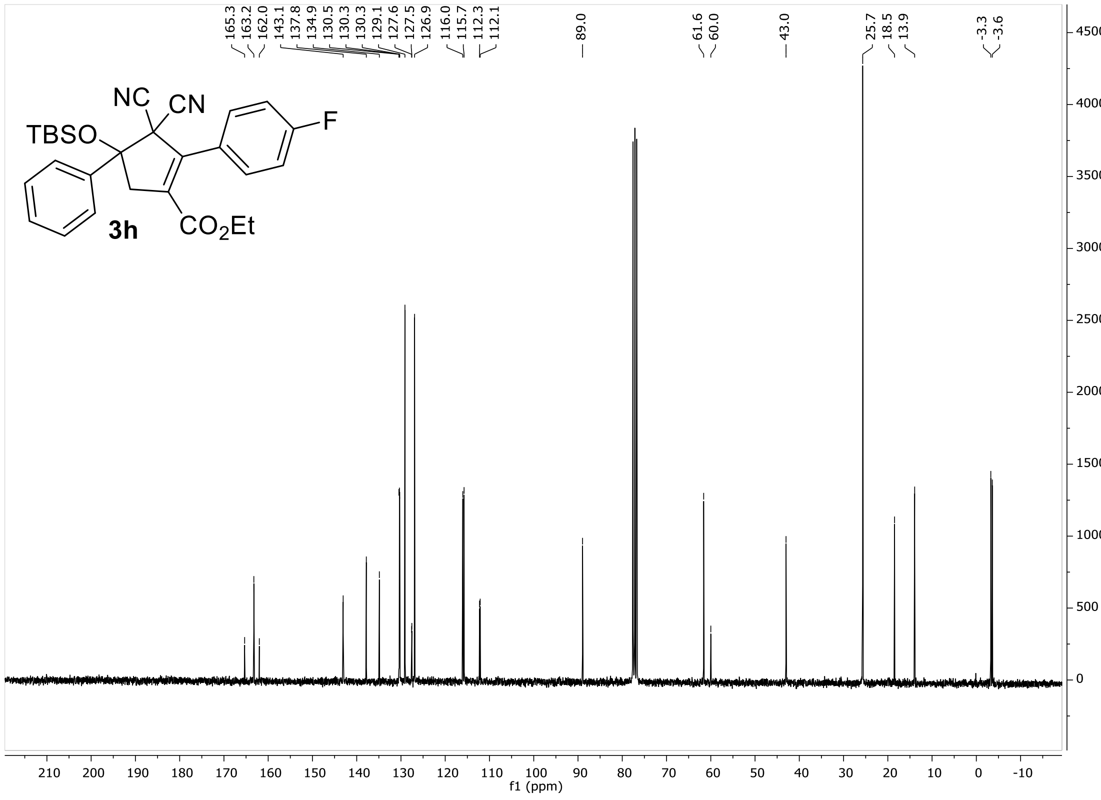

^{19}F{1H} NMR (376 MHz, $CDCl_3$) of **3h**:

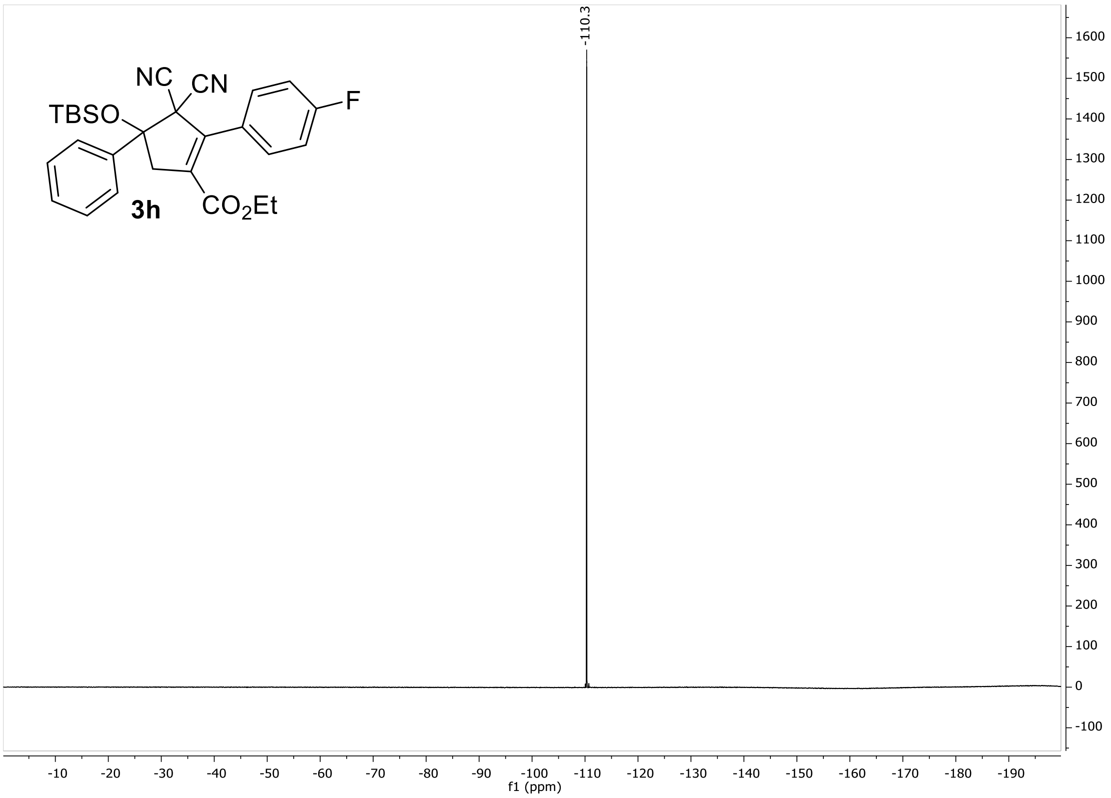

¹H NMR (300 MHz, $CDCl_3$) of **3i**:

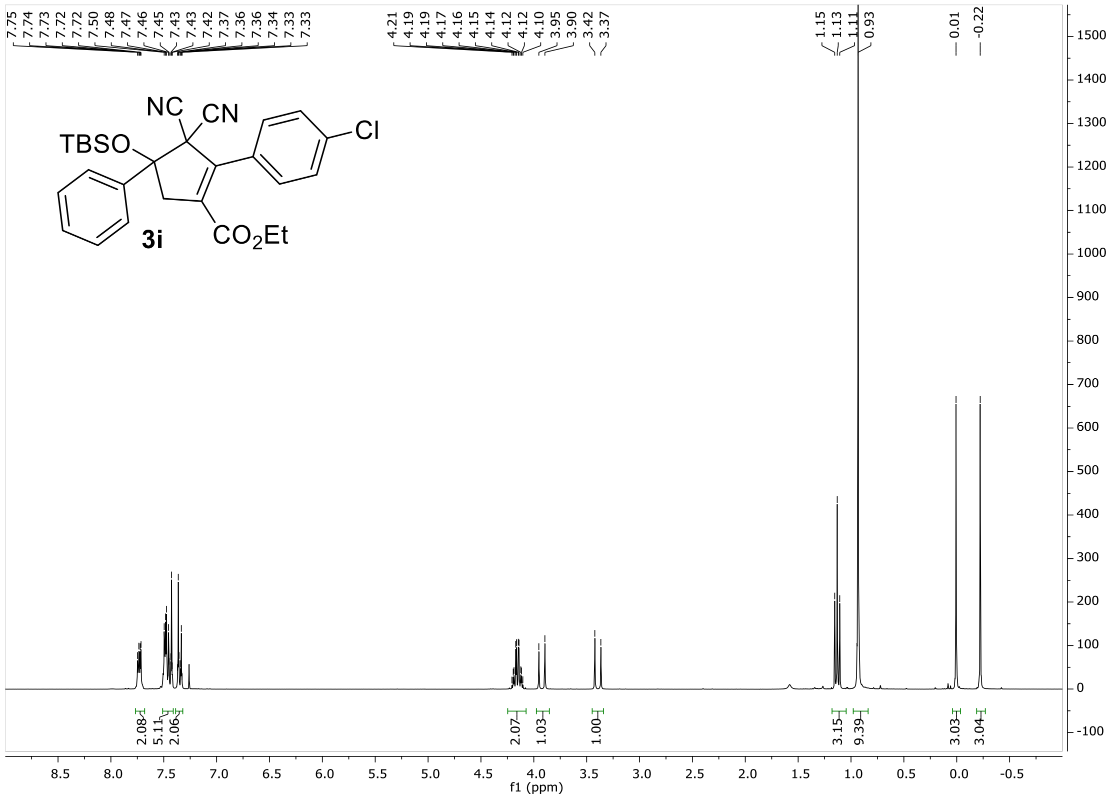

¹³C{1H} NMR (75 MHz, $CDCl_3$) of **3i**:

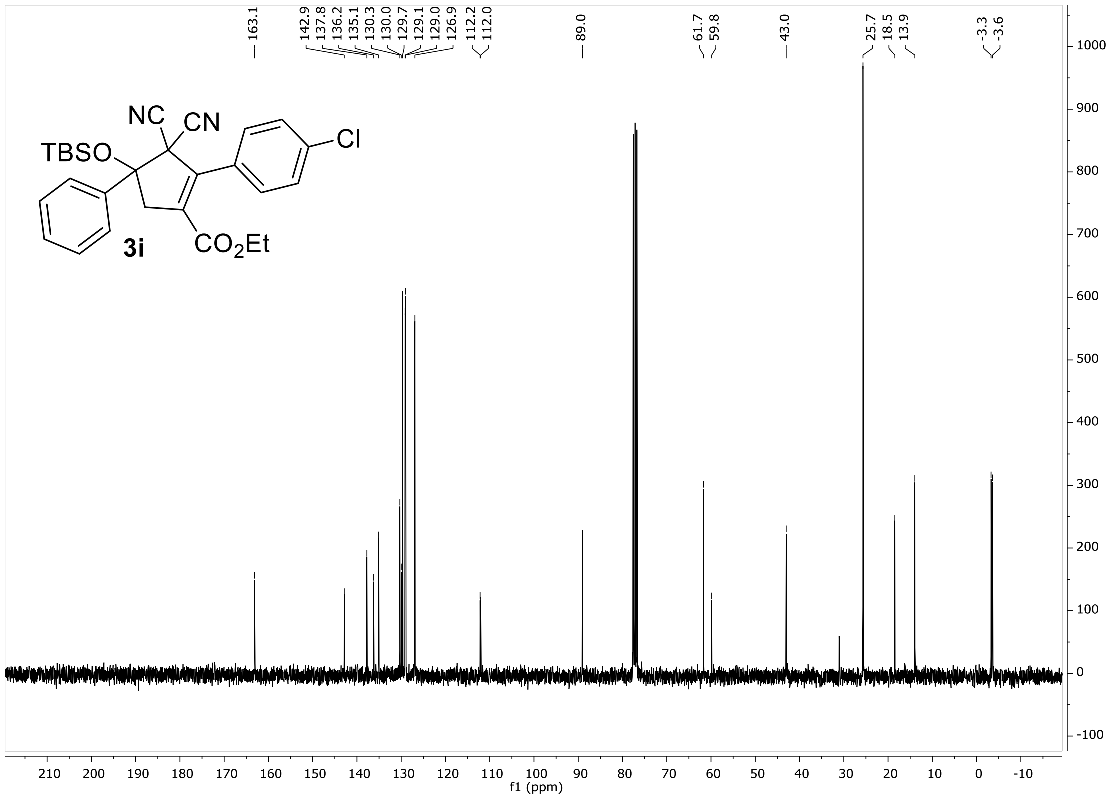

^{1}H NMR (300 MHz, $CDCl_3$) of **3j**:

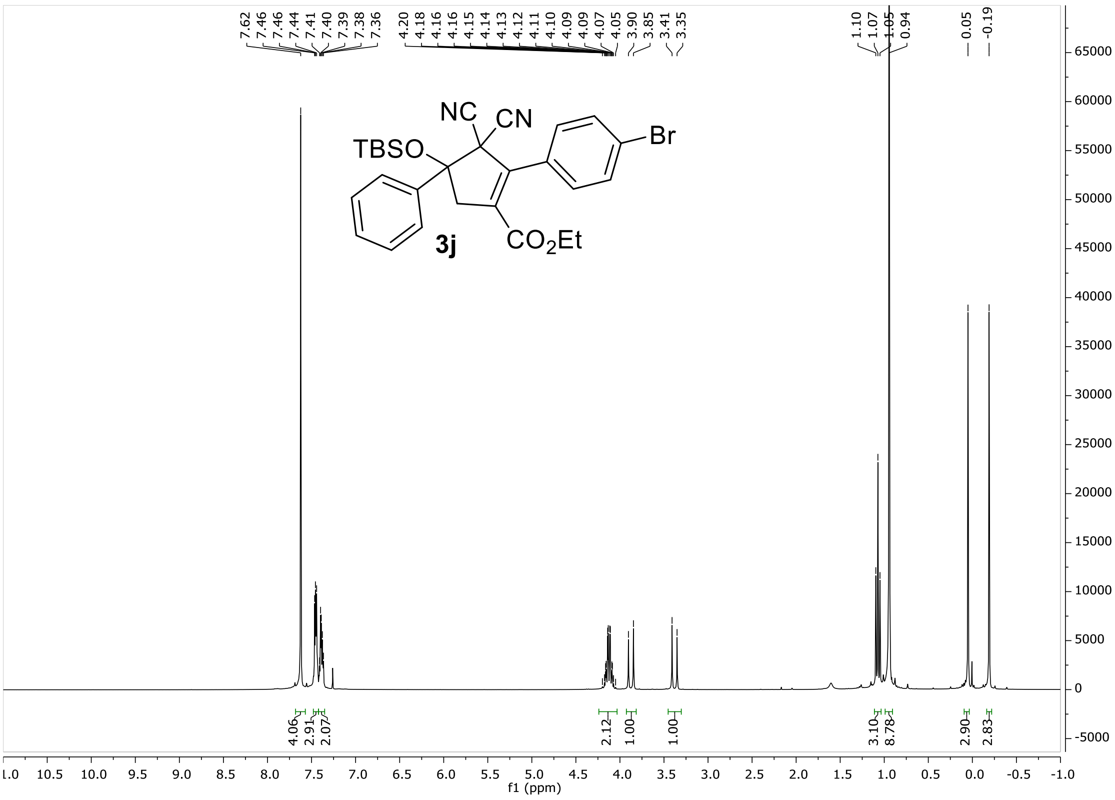

^{13}C{1H} NMR (75 MHz, $CDCl_3$) of **3j**:

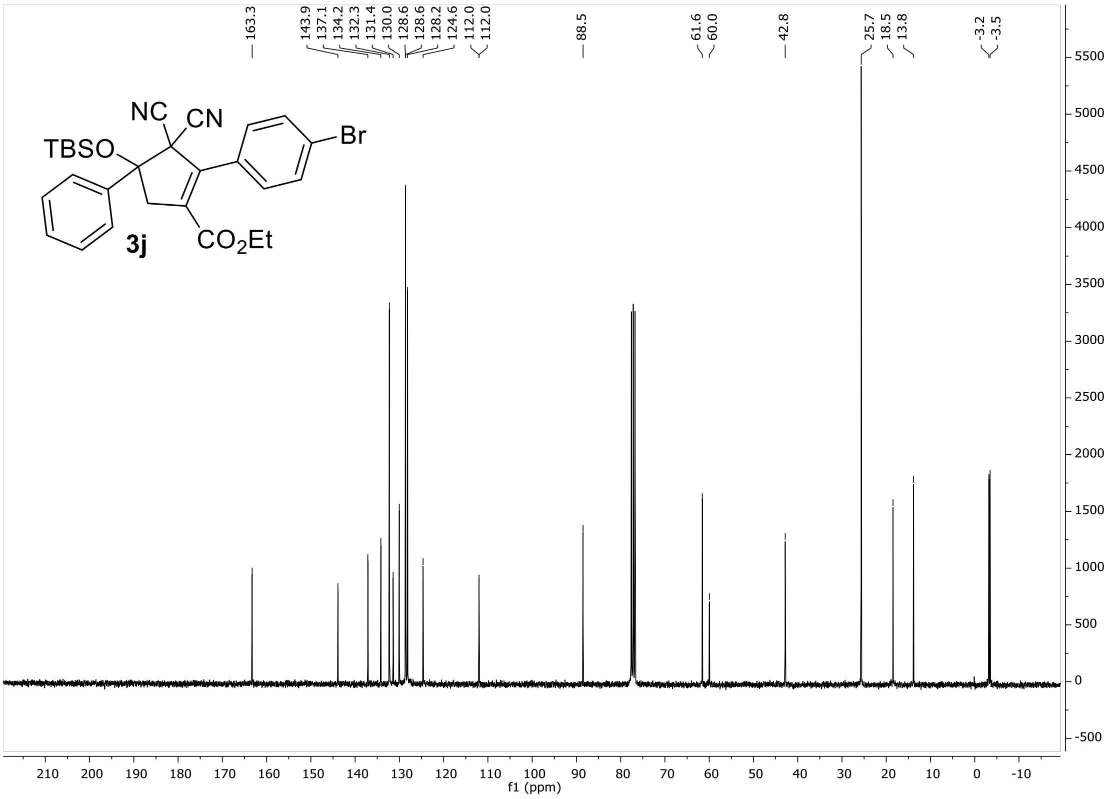

^{1}H NMR (300 MHz, $CDCl_3$) of **3k**:

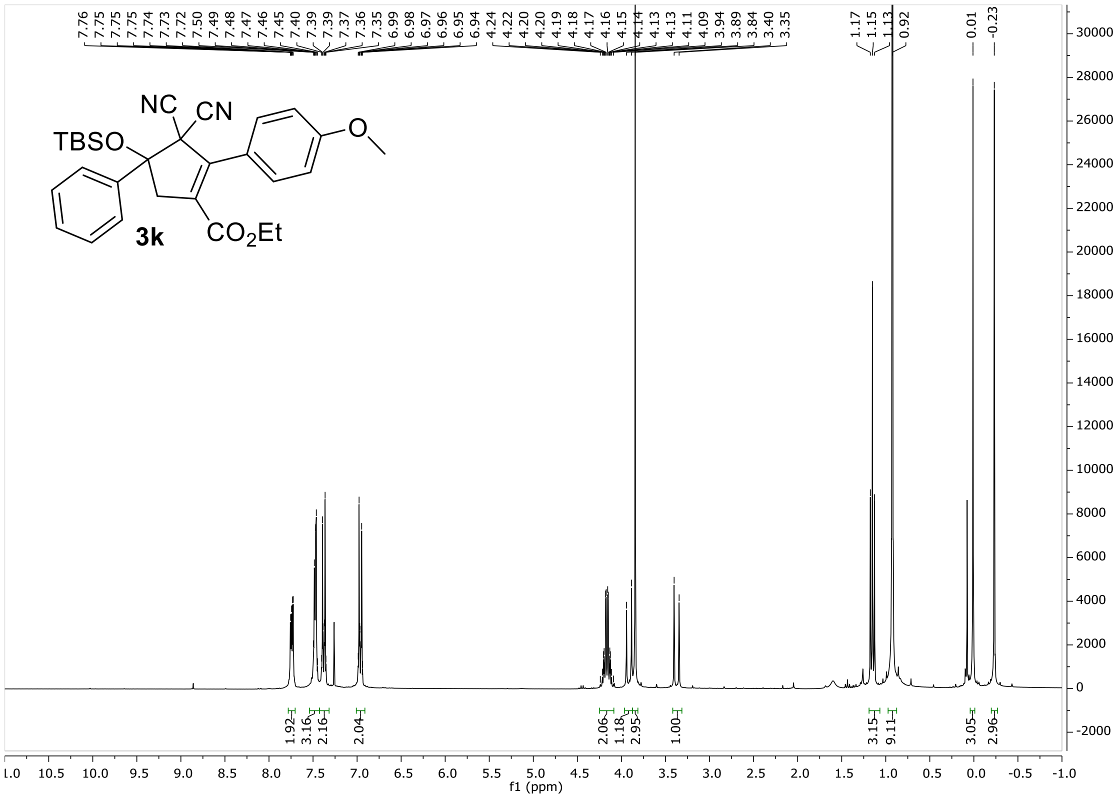

^{13}C{1H} NMR (75 MHz, $CDCl_3$) of **3k**:

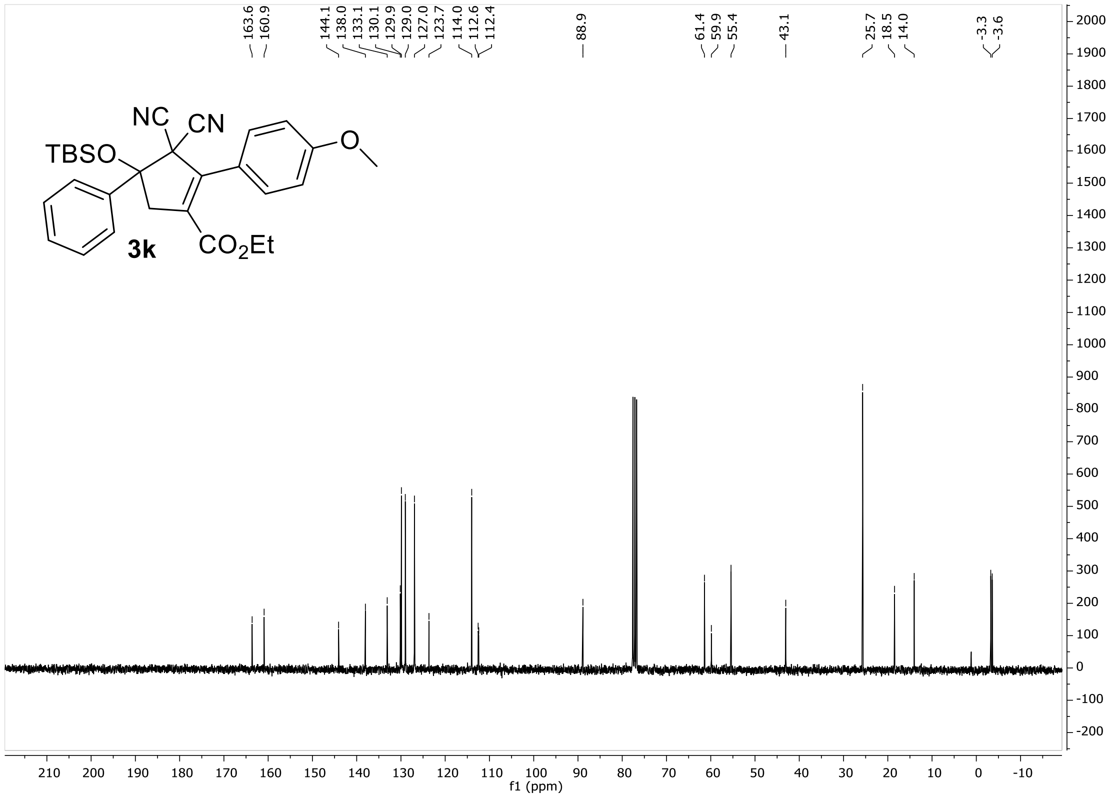

^{1}H NMR (300 MHz, $CDCl_3$) of **3m**:

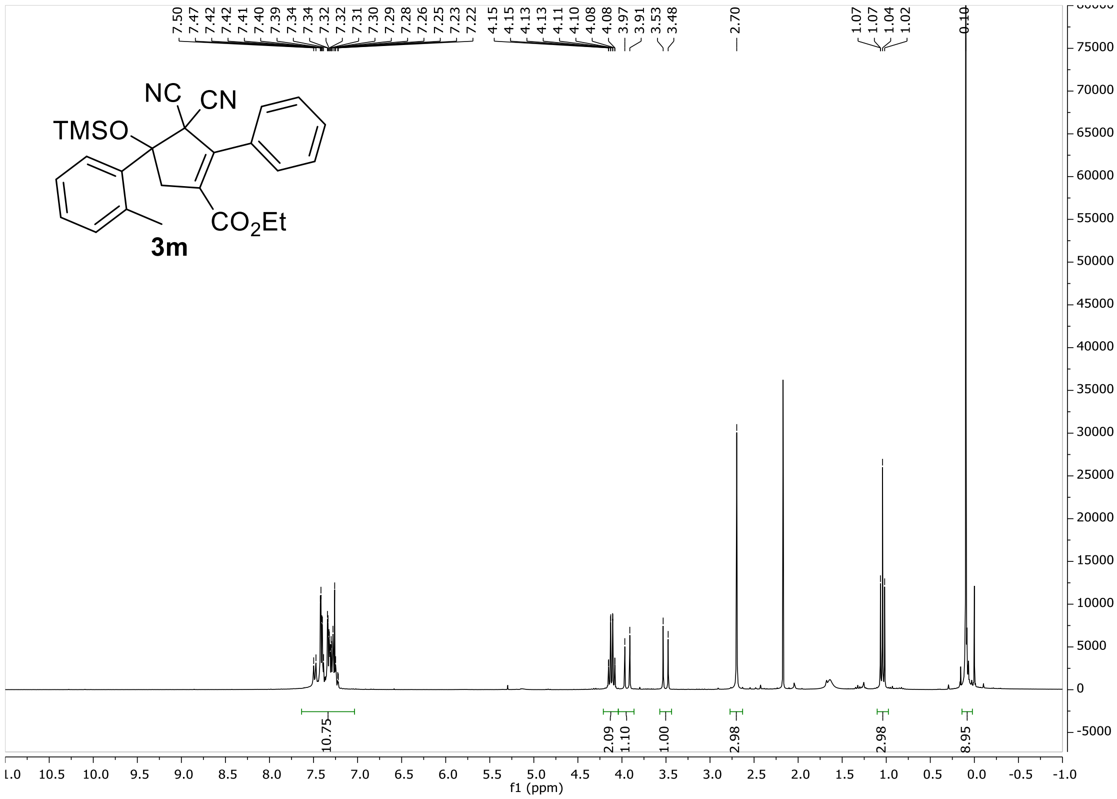

^{13}C{1H} NMR (75 MHz, $CDCl_3$) of **3m**:

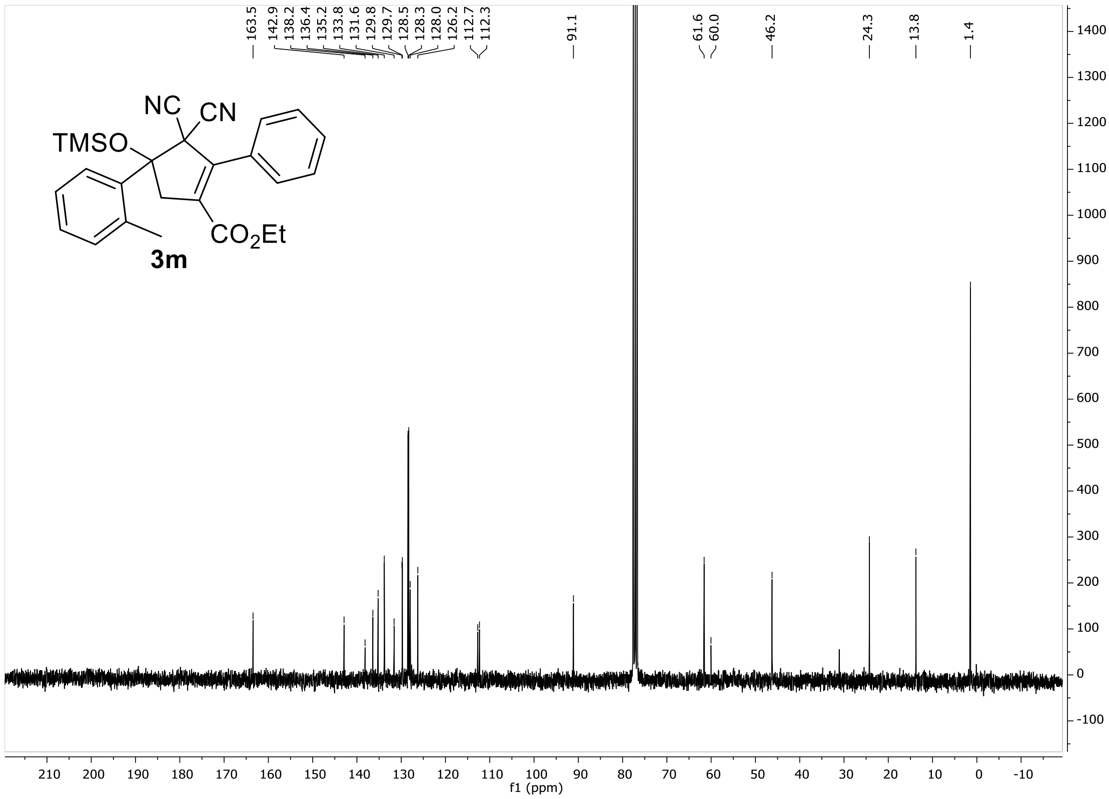

^{1}H NMR (300 MHz, $CDCl_3$) of **3n**:

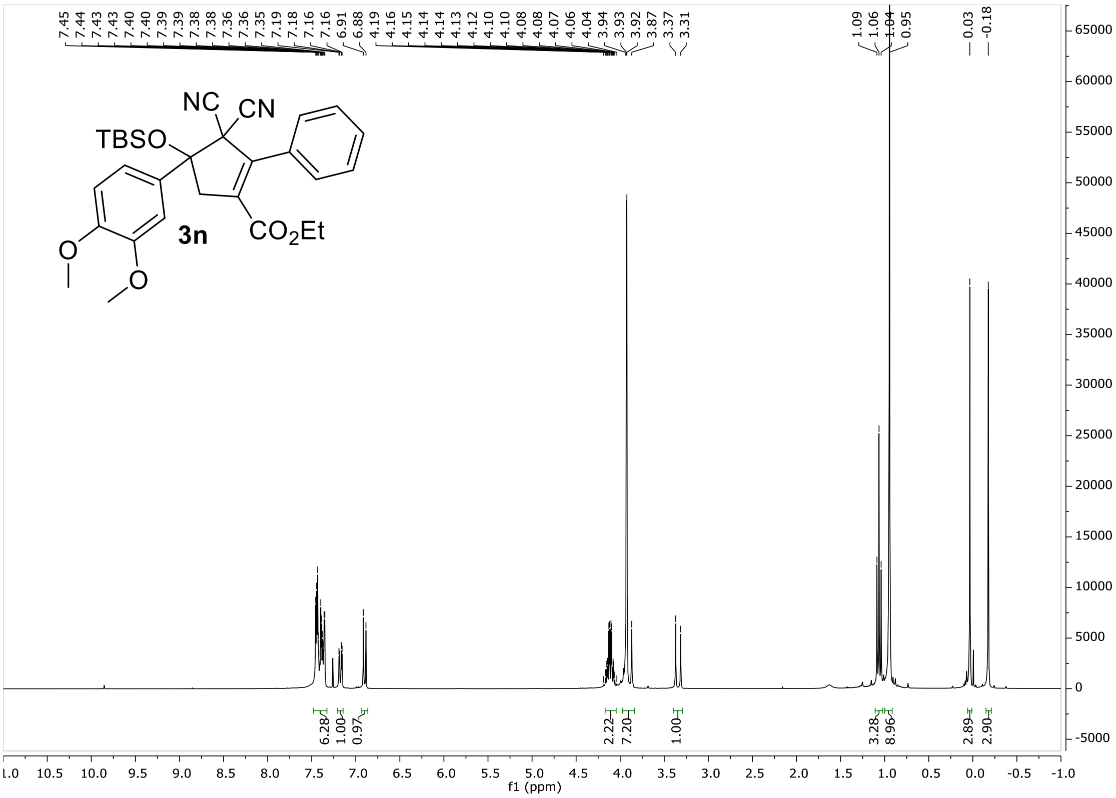

^{13}C{1H} NMR (75 MHz, $CDCl_3$) of **3n**:

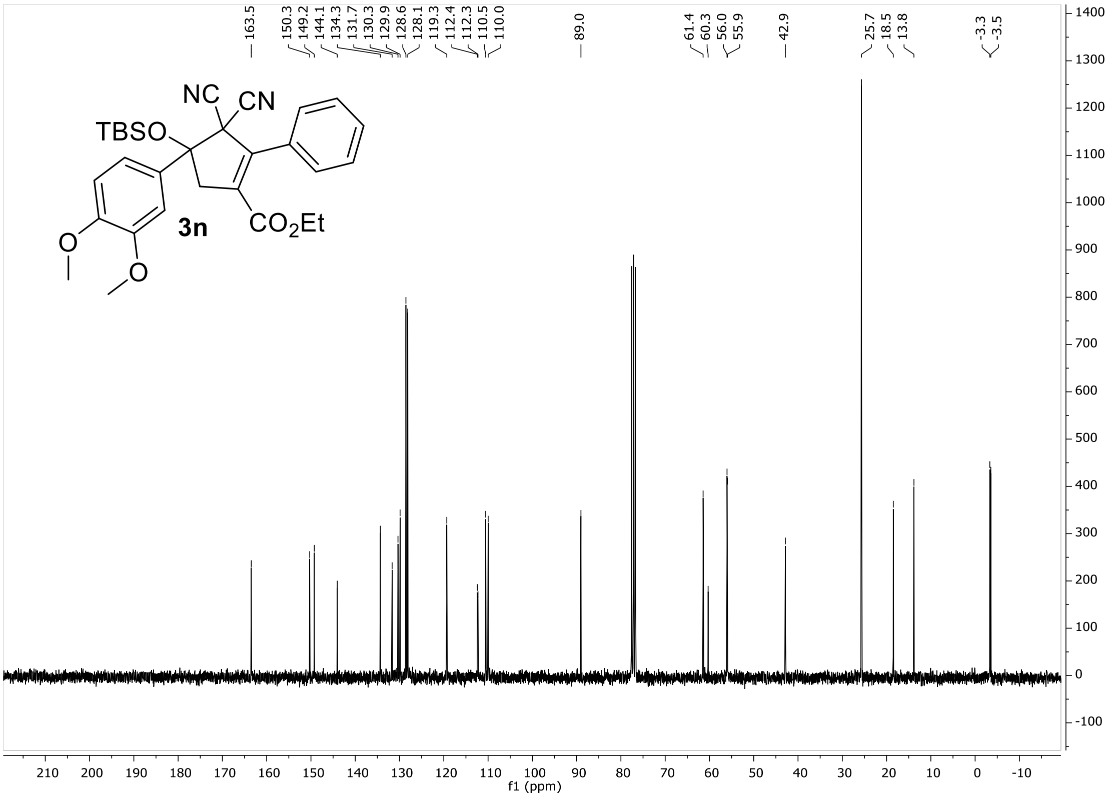

^{1}H NMR (400 MHz, $CDCl_3$) of **3o**:

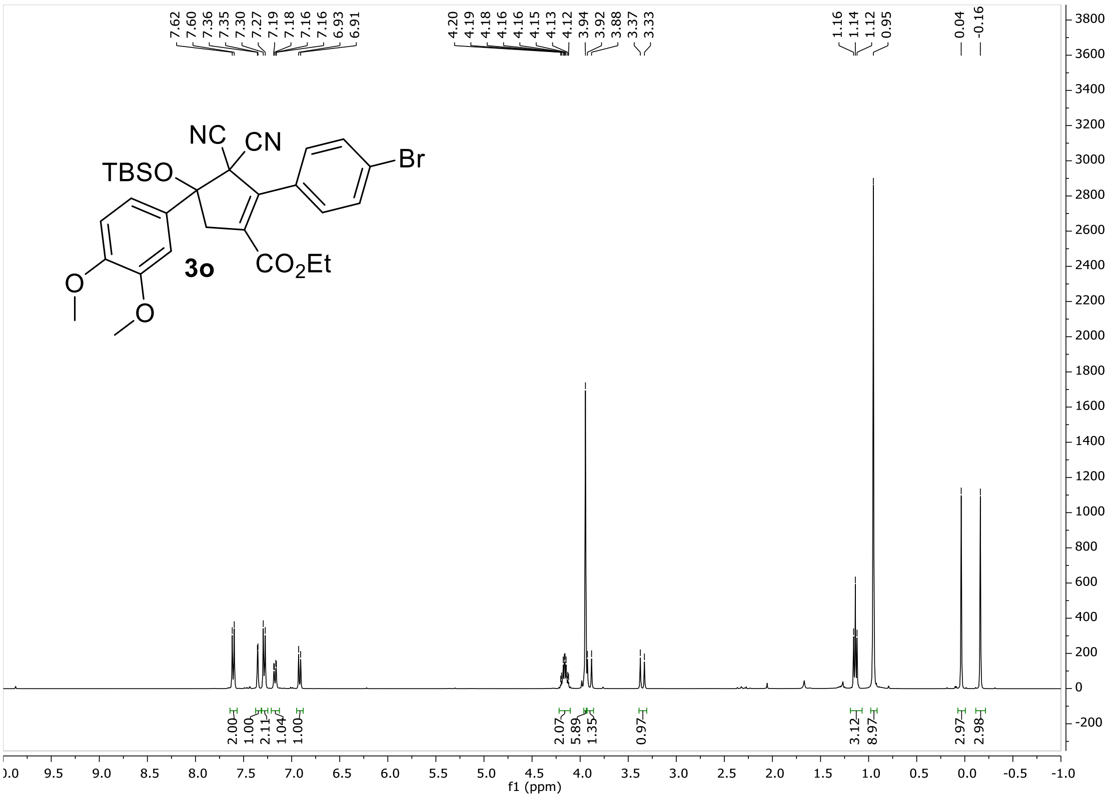

^{13}C{1H} NMR (100 MHz, $CDCl_3$) of **3o**:

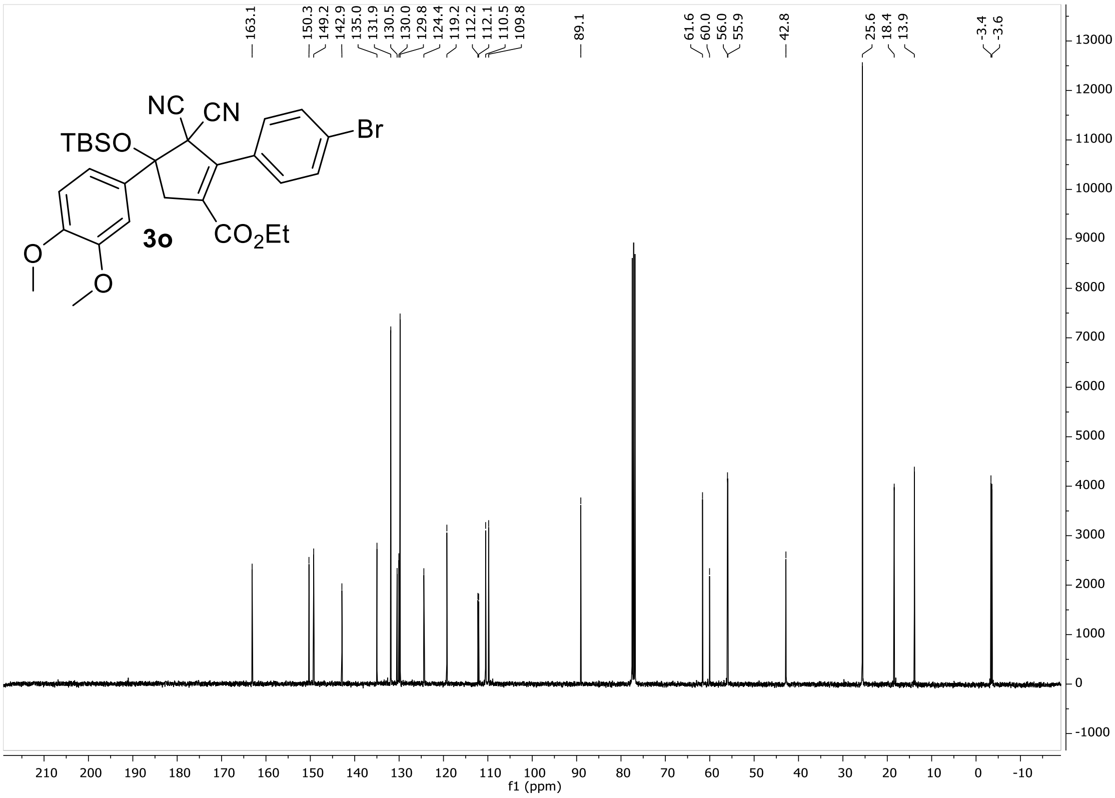

^{1}H NMR (400 MHz, $CDCl_3$) of **3p**:

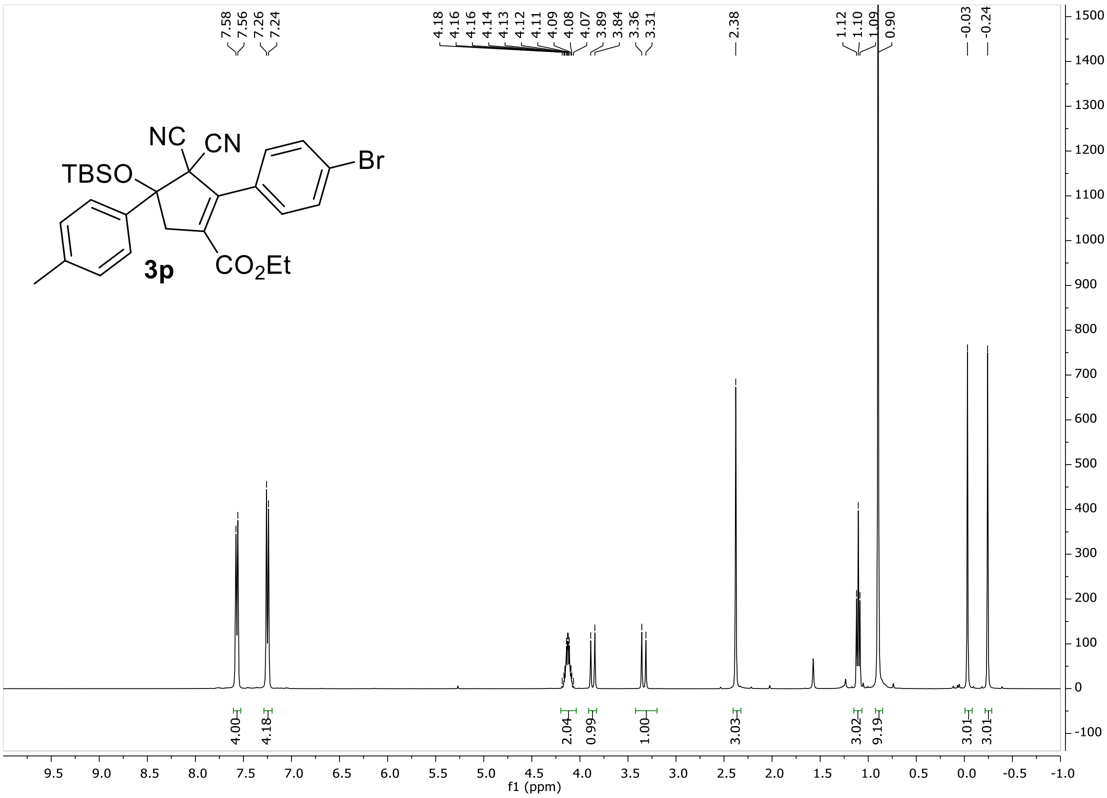

^{13}C{1H} NMR (100 MHz, $CDCl_3$) of **3p**:

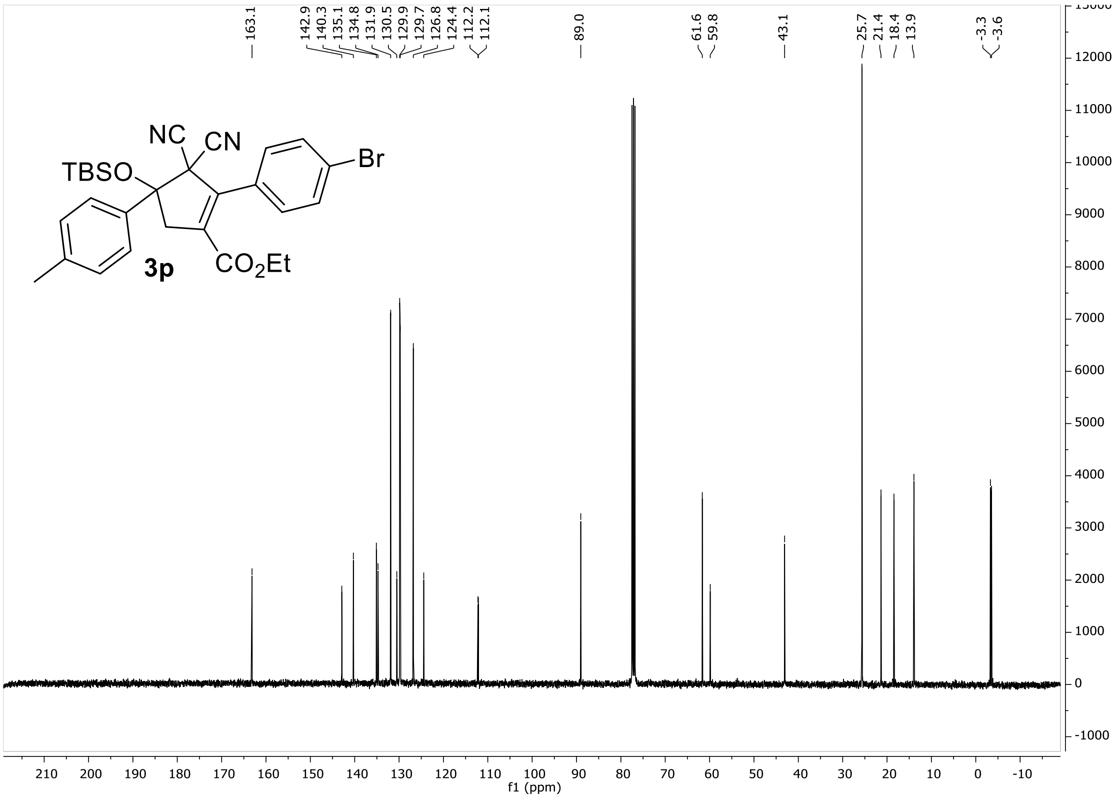

^{1}H NMR (400 MHz, $CDCl_3$) of **3q**:

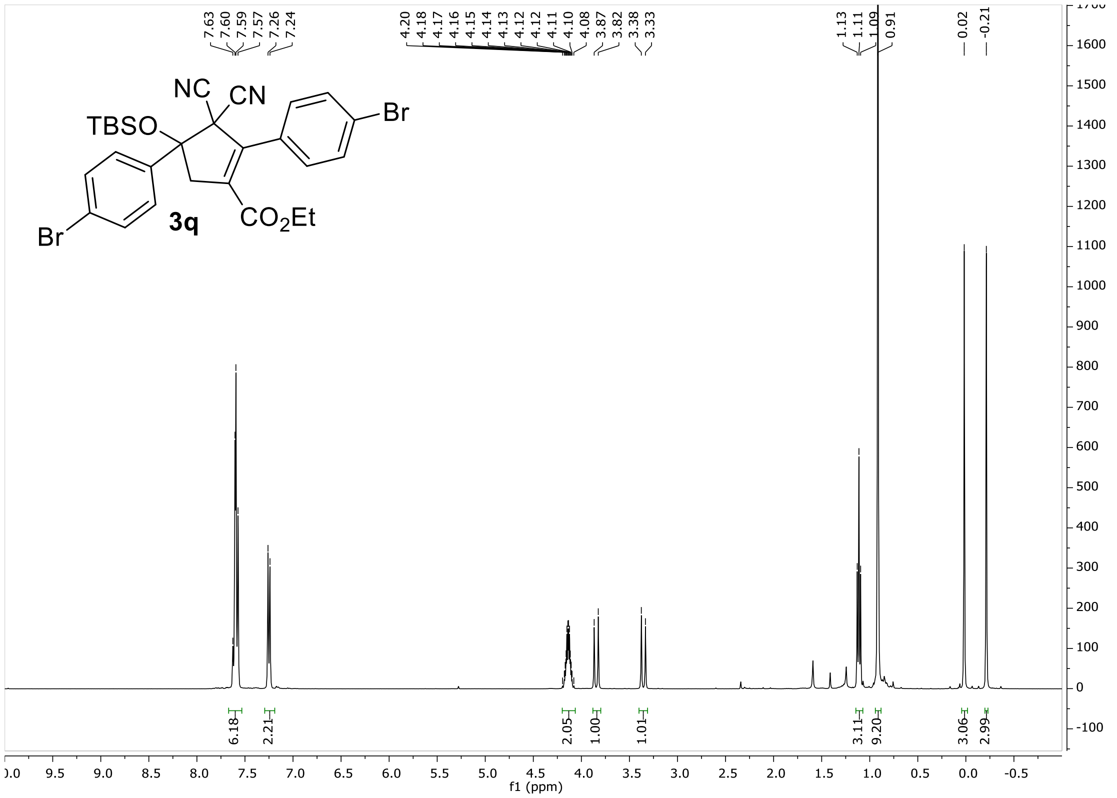

^{13}C{1H} NMR (100 MHz, $CDCl_3$) of **3q**:

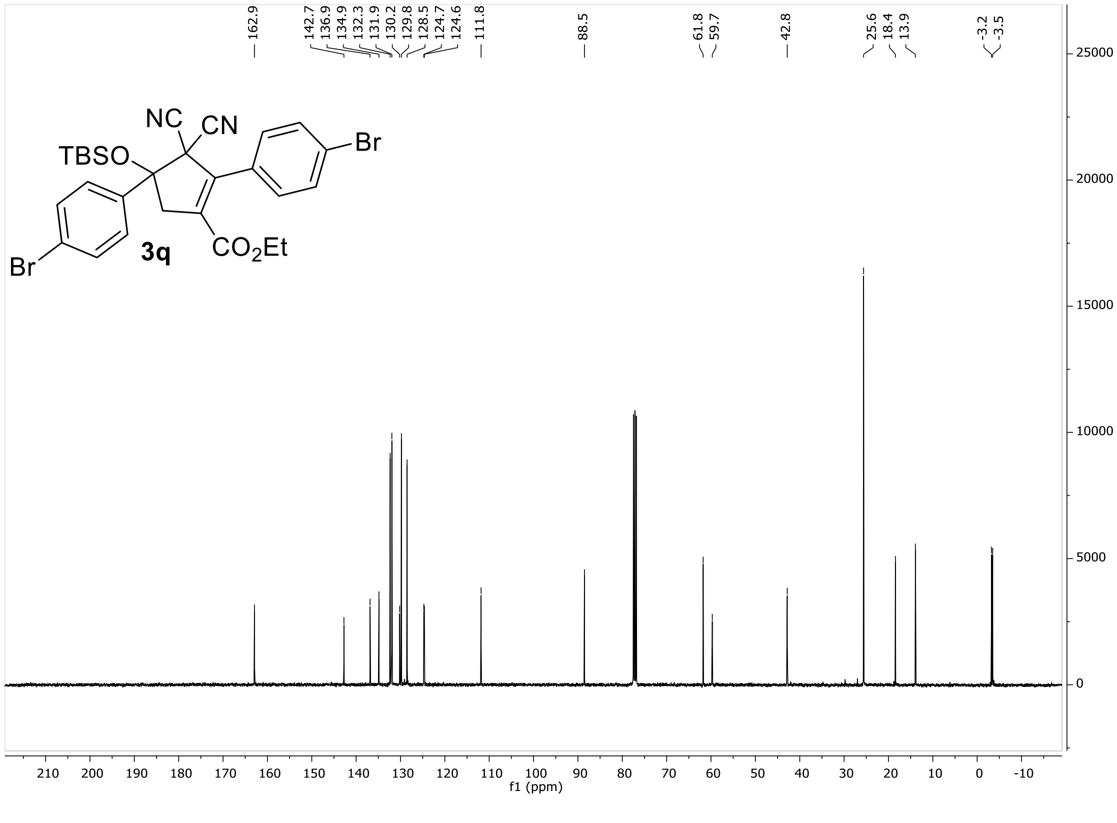

^{1}H NMR (400 MHz, $CDCl_3$) of **3r**:

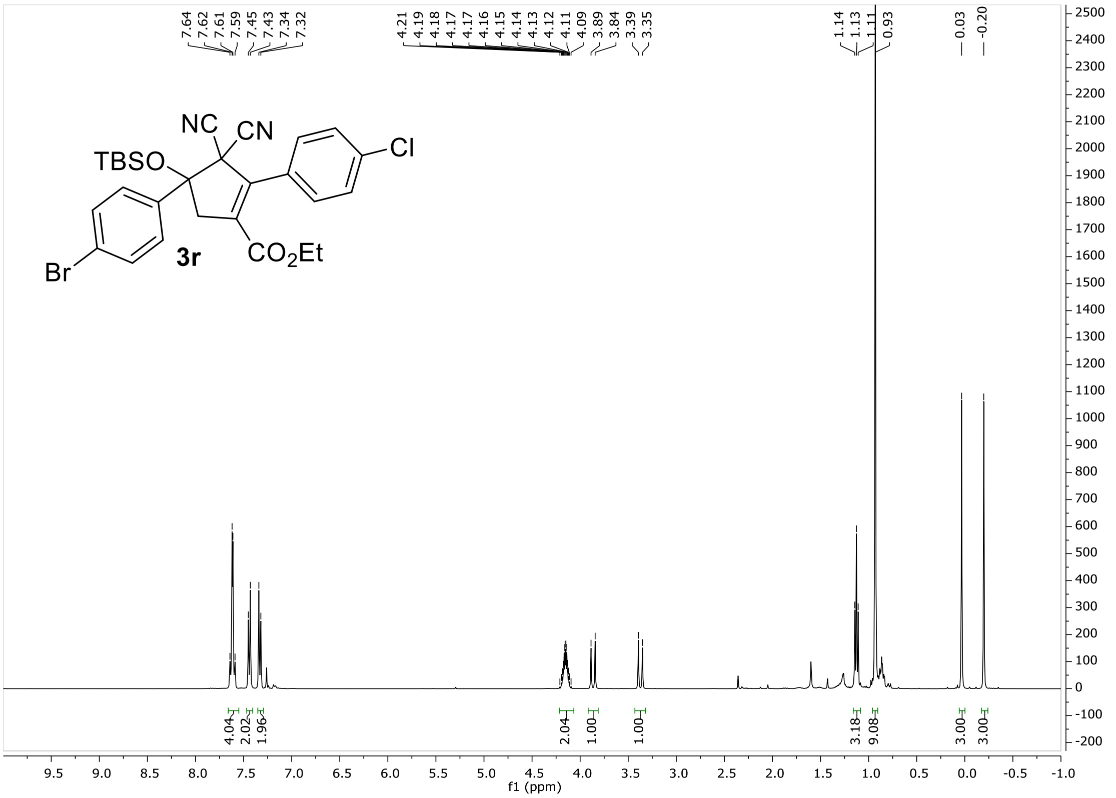

^{13}C{1H} NMR (100 MHz, $CDCl_3$) of **3r**:

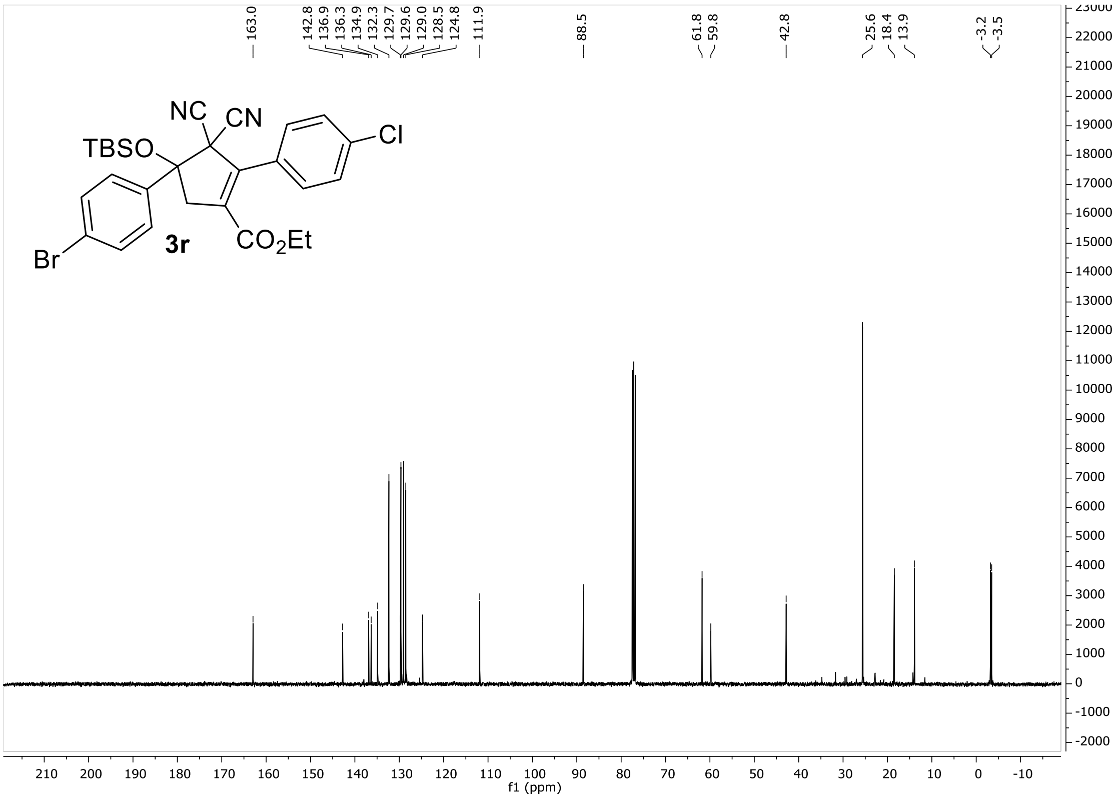

^{1}H NMR (300 MHz, $CDCl_3$) of **4**:

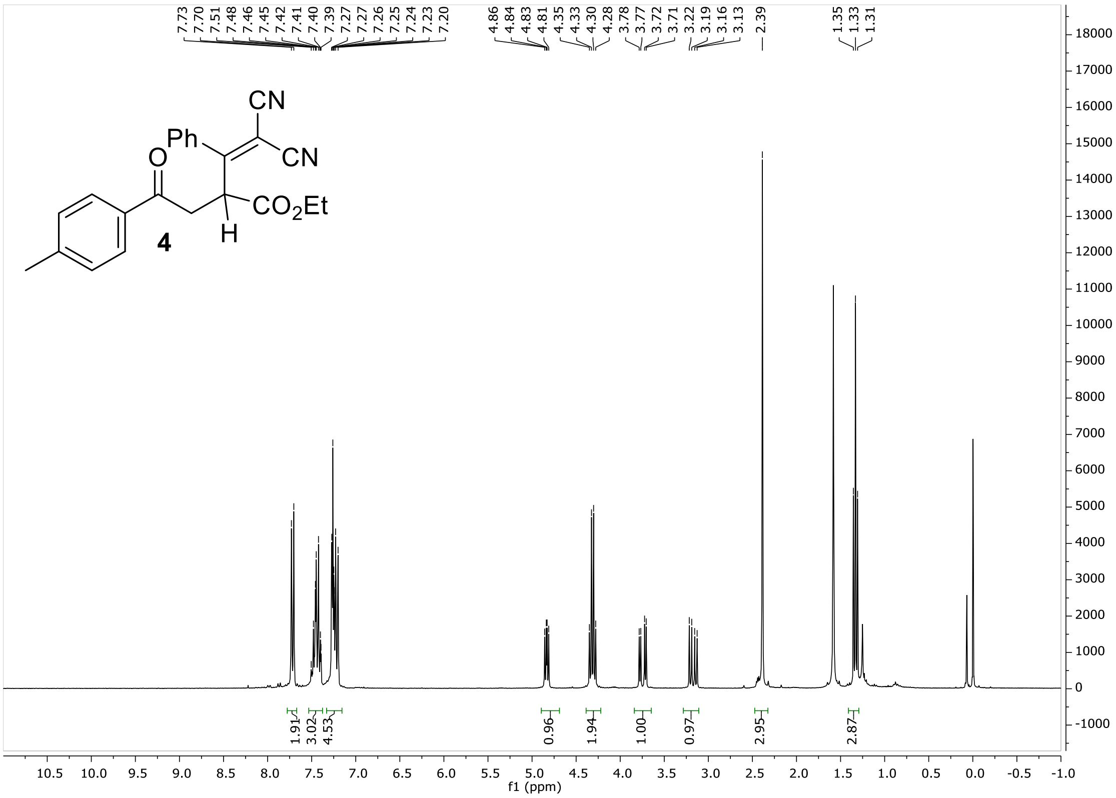

^{13}C{1H} NMR (75 MHz, $CDCl_3$) of **4**:

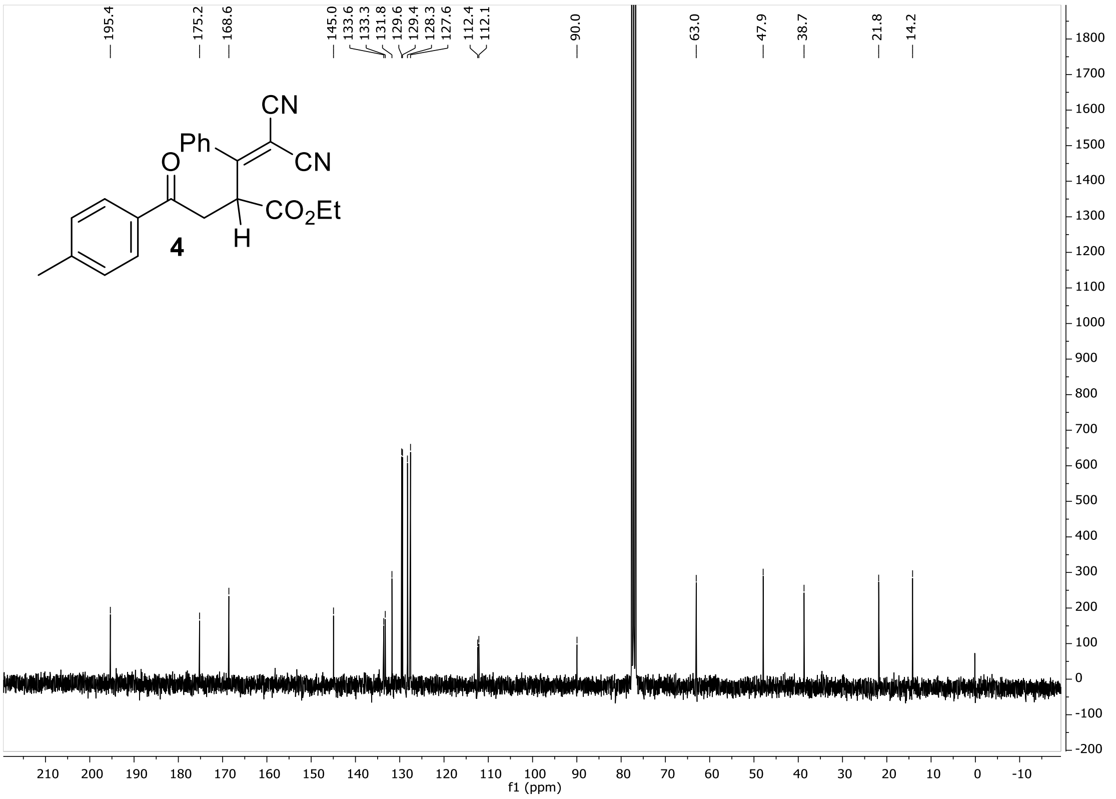

^{1}H NMR (300 MHz, $CDCl_3$) of **5**:

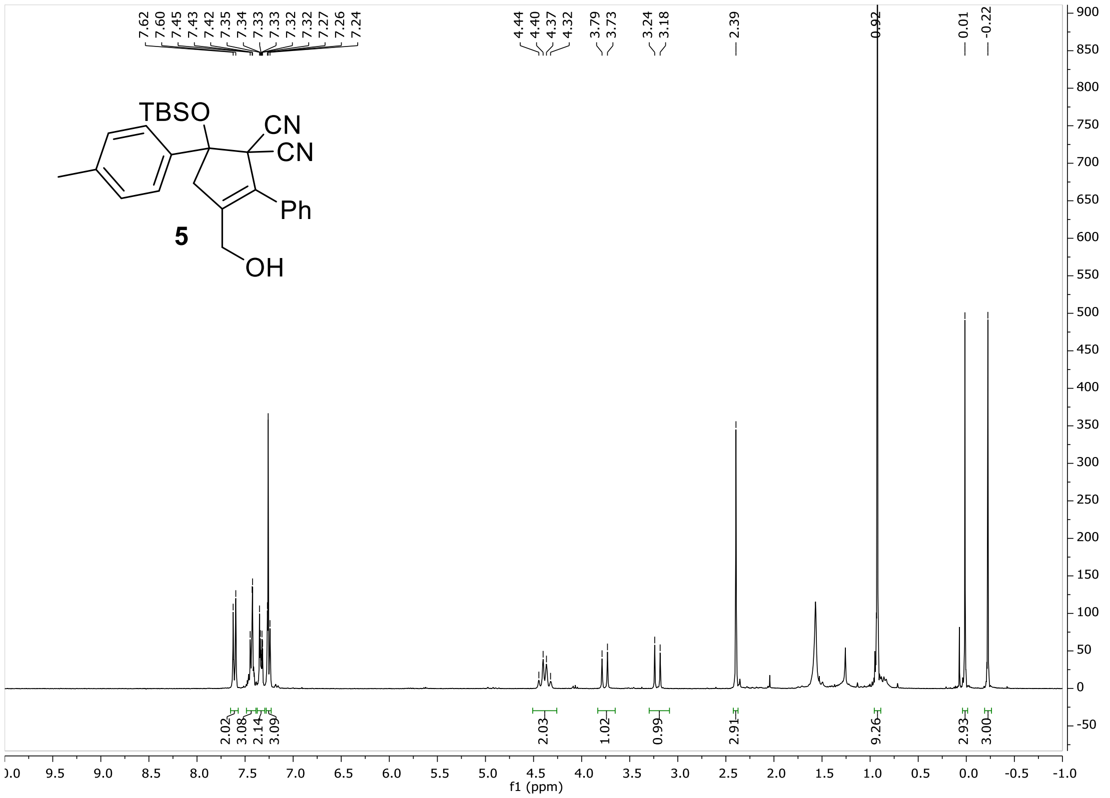

^{13}C{1H} NMR (100 MHz, $CDCl_3$) of **5**:

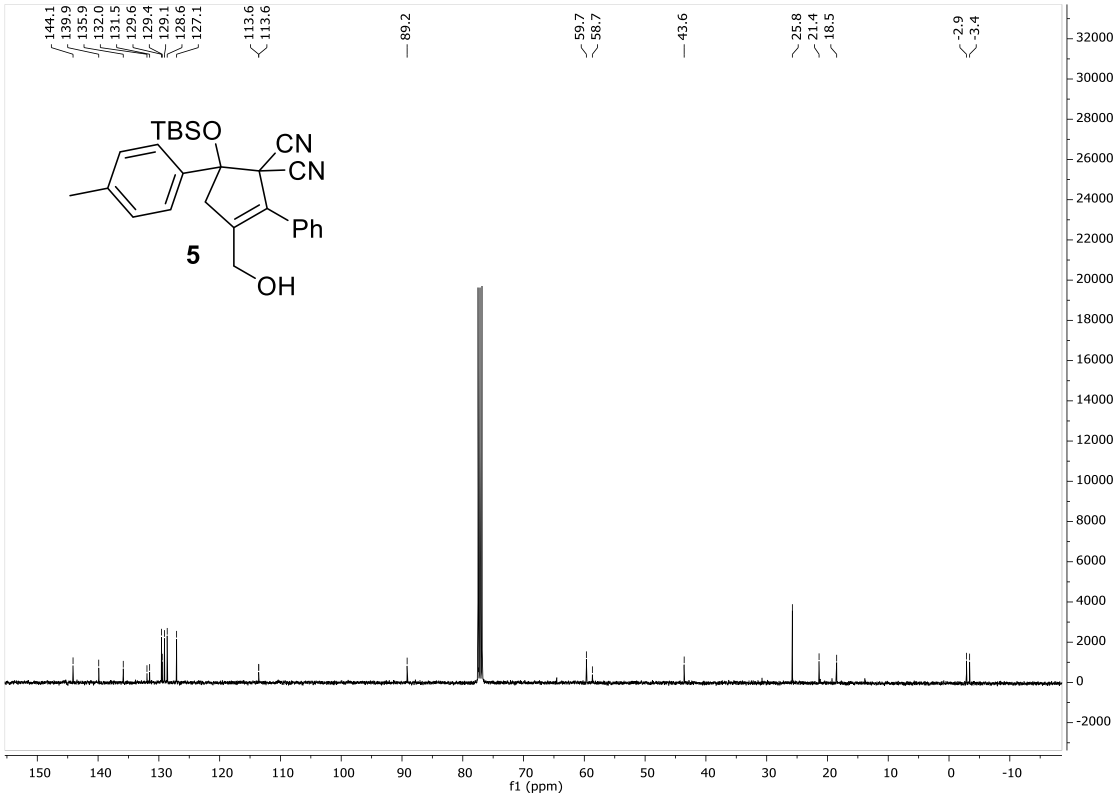

^{1}H NMR (400 MHz, $CDCl_3$) of **6**:

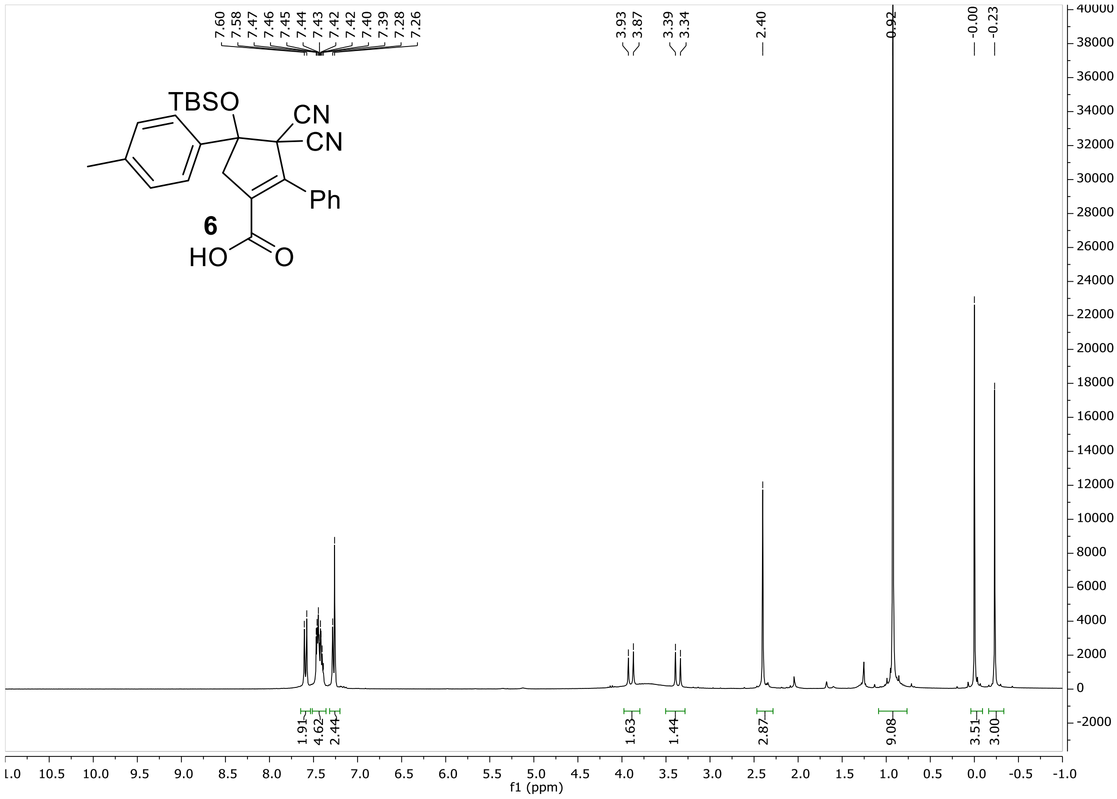

^{13}C{1H} NMR (100 MHz, $CDCl_3$) of **6**:

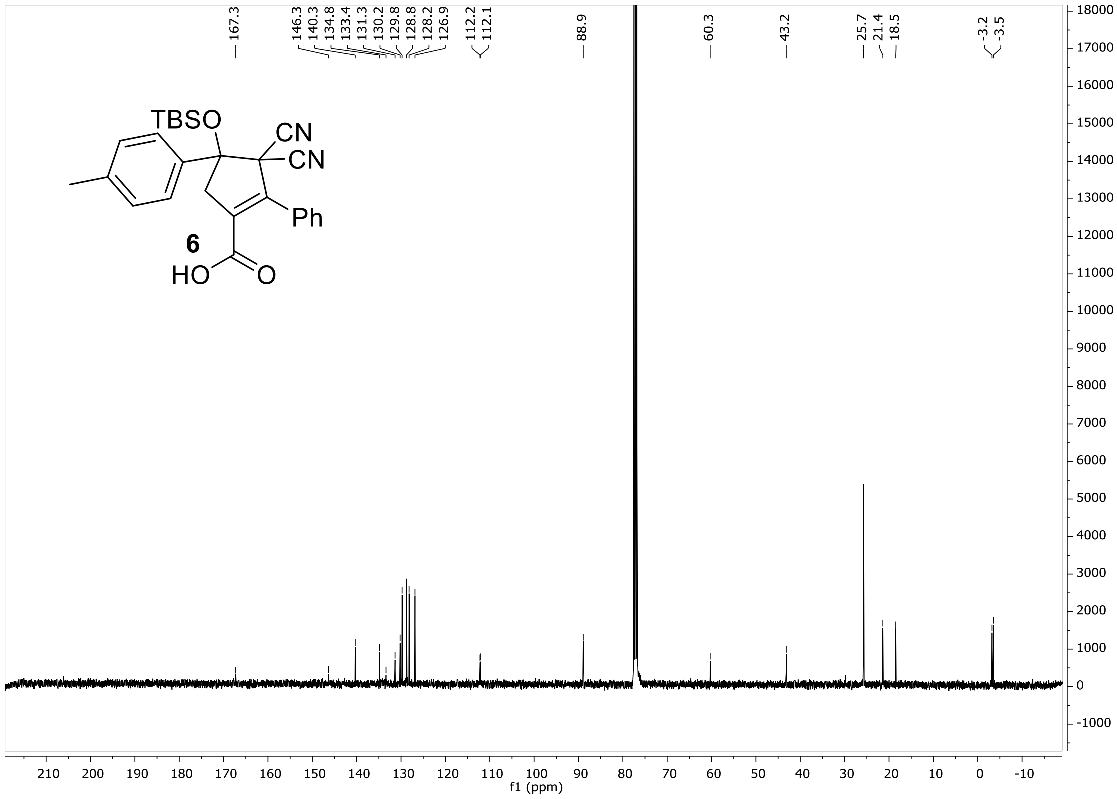

^{1}H NMR (400 MHz, $(CD_3)_2CO$) of **7**:

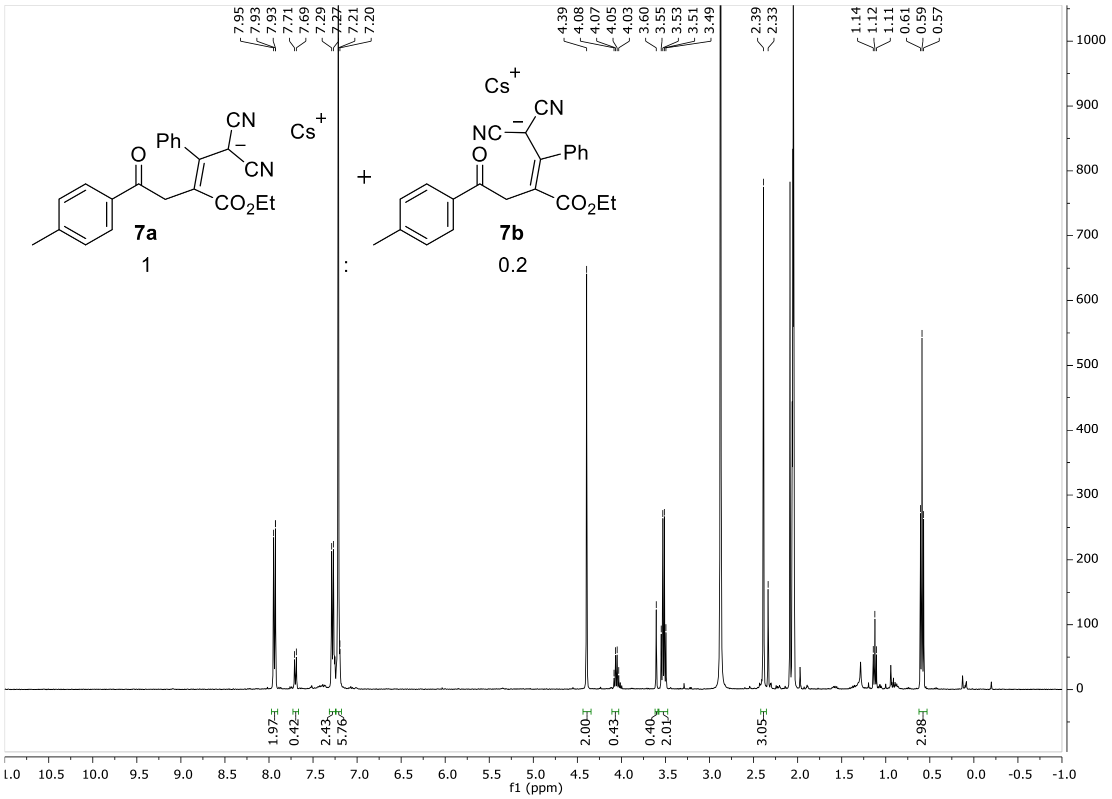

^{13}C{1H} NMR (100 MHz, $(CD_3)_2CO$) of **7**:

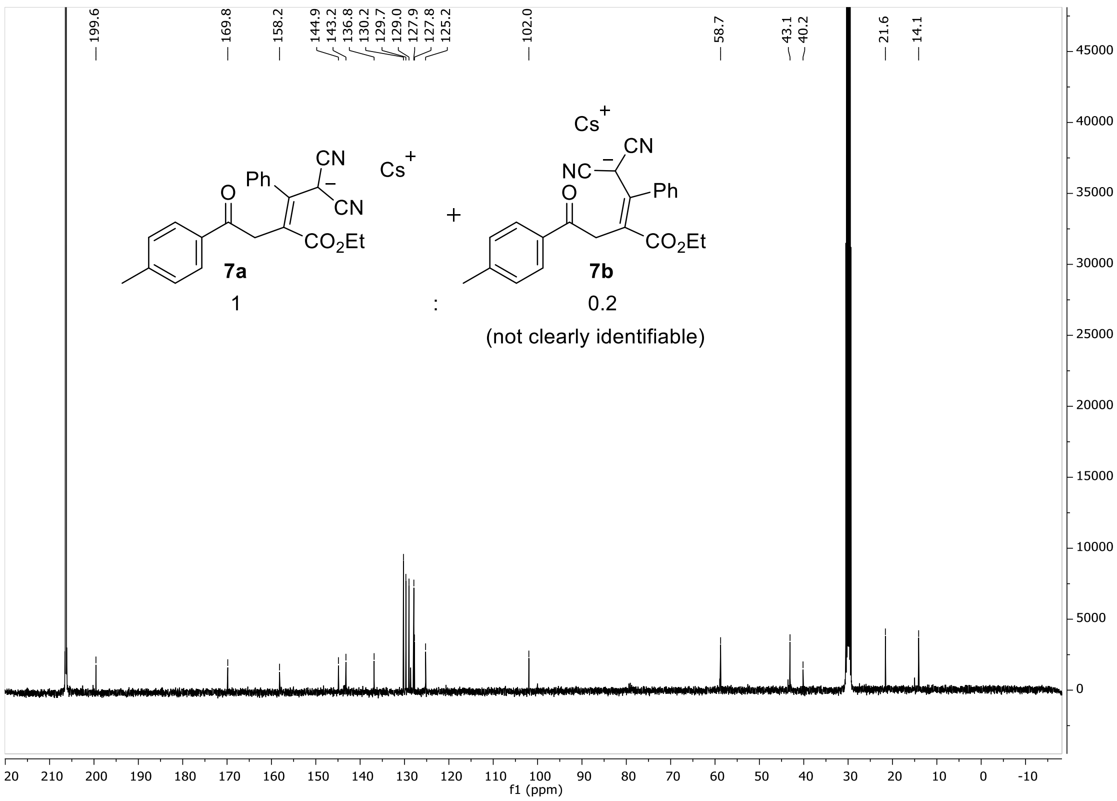

^{1}H NMR (100 MHz, $(CD_3)_2CO$) of **8**:

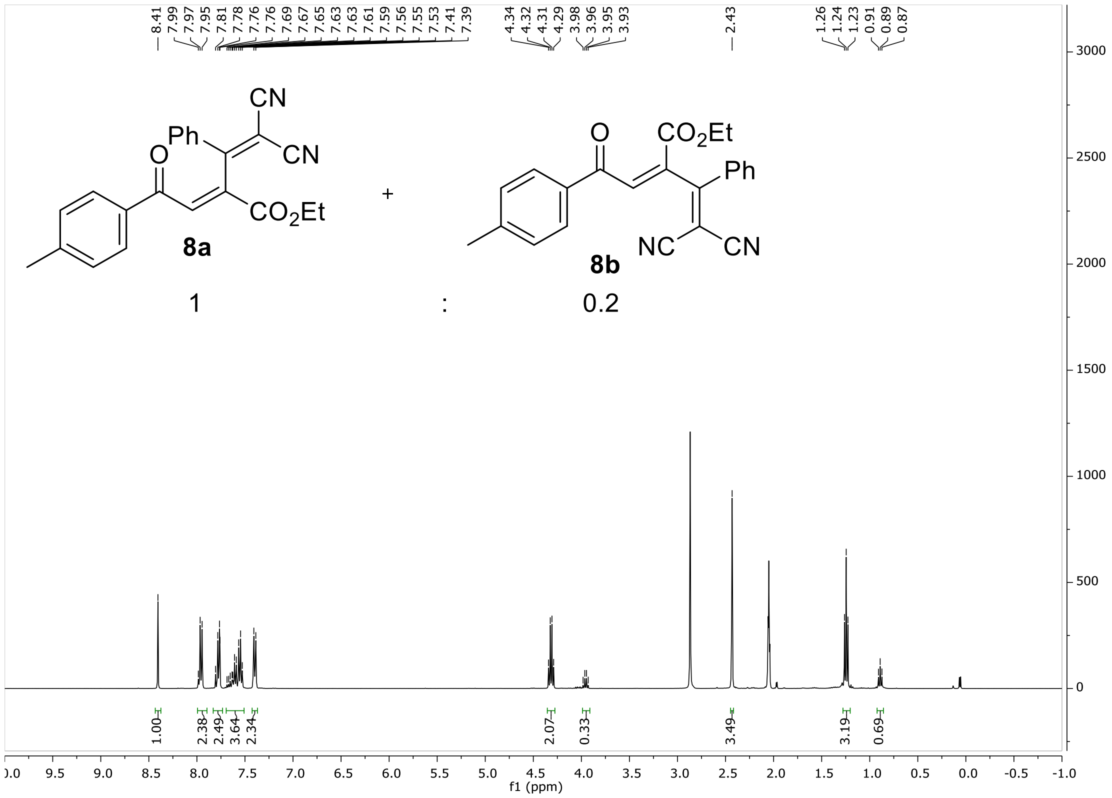

^{13}C{1H} NMR (100 MHz, $(CD_3)_2CO$) of **8**:

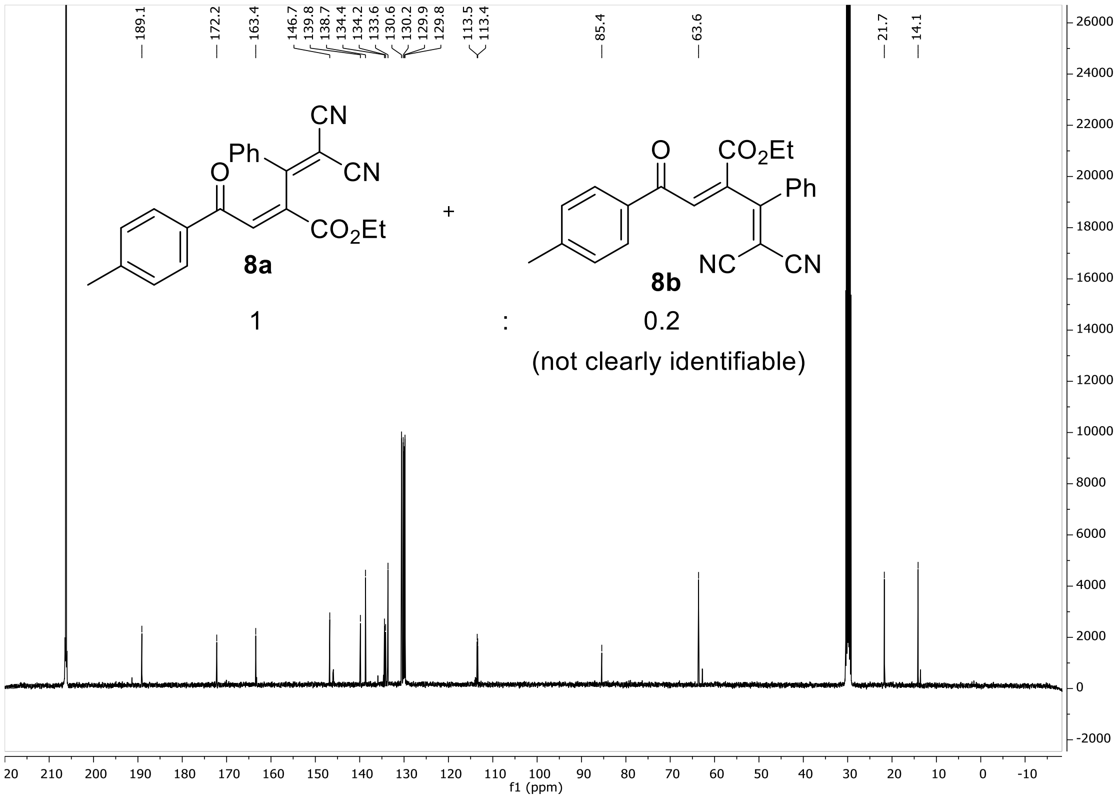

Atomic coordinates for all the optimized species (M06-2X/6-31+G**)

bzs

6	-4.344155	-0.275876	-1.818916
6	-3.653028	-0.539266	-0.633324
6	-4.325962	-0.525582	0.586399
6	-5.691634	-0.248527	0.620396
6	-6.388947	0.015764	-0.561124
6	-5.705362	0.000508	-1.783139
6	-7.860252	0.316367	-0.548161
14	-8.951439	0.361961	1.064338
8	-8.435942	0.549429	-1.602071
6	-10.672850	0.772259	0.454665
6	-8.938315	-1.322548	1.909212
6	-8.333444	1.704175	2.234147
1	-3.816557	-0.287517	-2.767509
1	-2.589401	-0.755111	-0.662430
1	-3.789614	-0.729949	1.507476
1	-6.210808	-0.239279	1.574412
1	-6.265958	0.208677	-2.689219
1	-11.378185	0.828612	1.289964
1	-11.024736	0.012156	-0.248422
1	-10.676911	1.731201	-0.070994
1	-9.671096	-1.331036	2.723433
1	-7.963696	-1.578573	2.333779
1	-9.215739	-2.112066	1.203968
1	-9.040369	1.823198	3.062455
1	-8.262127	2.666093	1.717137
1	-7.352471	1.478931	2.661527

sc[3]

6	-4.587795	2.362288	0.063071
6	-3.247477	2.220903	-0.309557
6	-2.730714	0.940507	-0.543956
6	-3.531713	-0.180663	-0.410543
6	-4.892451	-0.048930	-0.033002
6	-5.408652	1.253602	0.202820
6	-5.714436	-1.181141	0.105000
8	-6.990539	-1.206059	0.445157
14	-7.845711	-2.681638	0.590894
6	-9.569833	-2.156226	1.064331
6	-7.000016	-3.692175	1.915723
6	-7.771984	-3.538834	-1.067759
1	-4.995388	3.352249	0.246457
1	-2.613677	3.094720	-0.416224
1	-1.690946	0.822268	-0.833804
1	-3.129756	-1.172885	-0.592807
1	-6.448761	1.362315	0.491880
1	-10.221168	-3.027070	1.188242
1	-10.005216	-1.513315	0.293919
1	-9.561868	-1.601354	2.006933
1	-7.506597	-4.651338	2.063465
1	-6.992364	-3.159025	2.870999
1	-5.961807	-3.897712	1.634770
1	-8.314950	-4.489152	-1.045136
1	-6.733112	-3.749289	-1.342591
1	-8.209068	-2.914043	-1.852286

sc[1]

6	-4.723728	2.287685	0.068558
6	-3.371444	2.227279	-0.285806
6	-2.755671	0.998632	-0.517250
6	-3.497092	-0.174672	-0.393470
6	-4.850392	-0.128082	-0.039344
6	-5.459979	1.116810	0.191554
6	-5.547016	-1.434709	0.065409
8	-6.804041	-1.240293	0.405370
14	-7.767466	-2.671431	0.572754
6	-9.442973	-1.997980	1.052655
6	-7.016057	-3.718039	1.921494
6	-7.802468	-3.538930	-1.078085
1	-5.192713	3.250655	0.246320
1	-2.798748	3.145247	-0.380913
1	-1.706429	0.958348	-0.791781
1	-3.047276	-1.148154	-0.567356
1	-6.510106	1.144768	0.465969
1	-10.162856	-2.810574	1.193541
1	-9.833402	-1.331668	0.277566
1	-9.384037	-1.433155	1.987906
1	-7.620761	-4.613132	2.100281
1	-6.950539	-3.158970	2.859882
1	-6.007383	-4.028627	1.636155
1	-8.443114	-4.425886	-1.036769
1	-6.792557	-3.849815	-1.358765
1	-8.187751	-2.877305	-1.859877

dn

6	-8.338714	1.122389	0.787838
6	-8.357277	-0.029315	0.064108
6	-7.842460	-0.040770	-1.329083
6	-8.283605	0.795438	-2.273540
6	-8.942951	1.242975	2.089127
7	-9.426854	1.379105	3.131542
6	-7.731262	2.331854	0.295251
7	-7.255983	3.317660	-0.081388
6	-6.780934	-1.062559	-1.604737
8	-6.213134	-1.679415	-0.735308
8	-6.518137	-1.196538	-2.909786
6	-5.465832	-2.115760	-3.224294
6	-8.907629	-1.297364	0.585894
6	-9.679033	-2.102815	-0.263189
6	-10.216845	-3.297920	0.202294
6	-9.968310	-3.711037	1.510372
6	-9.180237	-2.926149	2.351175
6	-8.652351	-1.723571	1.894818
1	-7.891827	0.740723	-3.283048
1	-9.039819	1.542602	-2.053461
1	-5.377698	-2.098875	-4.308485
1	-5.723653	-3.115311	-2.869717
1	-4.534926	-1.795404	-2.752933
1	-9.870478	-1.780940	-1.283061
1	-10.826594	-3.907478	-0.456475
1	-10.381304	-4.647376	1.871697
1	-8.969945	-3.253214	3.363922
1	-8.017208	-1.130937	2.544465

[sc^{1}dn]$_1$

6	-8.229331	0.664828	0.740213
6	-7.881064	-0.295153	-0.161766
6	-6.728975	-0.052564	-1.068357
6	-6.647373	1.033678	-1.845830
6	-9.420212	0.622260	1.545605
7	-10.370423	0.641393	2.207304
6	-7.426511	1.839452	0.954863
7	-6.802989	2.789908	1.176620
6	-5.666079	-1.105931	-1.047929
8	-5.598120	-1.968140	-0.203716
8	-4.787155	-0.976892	-2.050752
6	-3.712176	-1.921383	-2.037330
6	-8.612204	-1.565636	-0.323834
6	-8.692418	-2.139308	-1.599886
6	-9.372305	-3.336449	-1.792100
6	-9.965517	-3.983639	-0.710272
6	-9.869451	-3.431934	0.567440
6	-9.195727	-2.232297	0.763233
1	-5.797311	1.159634	-2.508272
1	-7.428264	1.794940	-1.837883
1	-3.096603	-1.676110	-2.900368
1	-4.103779	-2.937421	-2.114646
1	-3.140063	-1.826717	-1.112412
1	-8.246780	-1.627329	-2.447887
1	-9.442892	-3.759631	-2.789228
1	-10.491672	-4.921544	-0.858443
1	-10.309511	-3.943182	1.417239
1	-9.095420	-1.834476	1.766642
6	-12.003234	-0.744618	-1.734706
6	-11.663935	-1.156848	-3.027265
6	-10.730970	-0.440927	-3.775539
6	-10.128856	0.687930	-3.222612
6	-10.488237	1.132686	-1.944183
6	-11.433454	0.404752	-1.200606
6	-9.844057	2.393775	-1.483528
8	-10.493868	2.859513	-0.448163
14	-10.188108	4.429385	0.232963
6	-8.675658	5.186678	-0.544501
6	-10.052677	4.153188	2.071762
6	-11.737796	5.383163	-0.196467
1	-12.717784	-1.319061	-1.153102
1	-12.123454	-2.048148	-3.445274
1	-10.469656	-0.766477	-4.777914
1	-9.390371	1.260780	-3.778634
1	-11.705014	0.750051	-0.207347
1	-8.505592	6.179124	-0.112148
1	-8.808755	5.293198	-1.624287
1	-7.786351	4.577947	-0.363770
1	-10.872472	3.522715	2.428855
1	-10.105737	5.114364	2.594481
1	-9.107436	3.676245	2.343986
1	-11.693740	6.397808	0.212662
1	-12.625687	4.890551	0.210734
1	-11.856771	5.459872	-1.281648

ts1$_{trans}$

6	-8.005704	0.528281	1.780209
6	-7.921970	-0.212606	0.626473
6	-7.206497	0.280104	-0.560662
6	-7.154001	1.596556	-0.904266
6	-8.904294	0.209843	2.856748
7	-9.628003	0.019297	3.741232
6	-7.235295	1.716050	2.020975
7	-6.645213	2.688008	2.246733
6	-6.509293	-0.732440	-1.399583
8	-6.222301	-1.850058	-1.036907
8	-6.173792	-0.256382	-2.616795
6	-5.437387	-1.167547	-3.434165
6	-8.615975	-1.519727	0.545292
6	-9.303755	-1.871464	-0.623325
6	-9.977543	-3.083952	-0.704990
6	-9.948655	-3.973087	0.368880
6	-9.240841	-3.643801	1.523624
6	-8.580559	-2.423139	1.614284
1	-6.593200	1.914671	-1.773577
1	-7.488129	2.375433	-0.236975
1	-5.241764	-0.636396	-4.364377
1	-6.025082	-2.069890	-3.617102
1	-4.502154	-1.446396	-2.944548
1	-9.314288	-1.190599	-1.468423
1	-10.518208	-3.334180	-1.612619
1	-10.466875	-4.924596	0.302274
1	-9.197450	-4.339713	2.354822
1	-8.011119	-2.184723	2.506591
6	-11.702531	-0.577825	-3.044043
6	-10.892281	-1.178302	-4.012727
6	-9.617784	-0.676377	-4.279996
6	-9.150543	0.426793	-3.570173
6	-9.948726	1.026414	-2.588291
6	-11.233735	0.520732	-2.333756
6	-9.351388	2.168437	-1.854234
8	-10.200309	2.662432	-0.999936
14	-10.019849	4.126275	-0.072354
6	-8.301815	4.827935	-0.277120
6	-10.432551	3.621201	1.670930
6	-11.315598	5.245935	-0.815502
1	-12.691680	-0.976811	-2.842037
1	-11.258412	-2.041340	-4.561277
1	-8.994429	-1.146432	-5.034044
1	-8.154138	0.823195	-3.742133
1	-11.842065	0.987328	-1.565359
1	-8.344516	5.914953	-0.150294
1	-7.916596	4.618679	-1.280143
1	-7.599066	4.436975	0.464985
1	-11.408081	3.128794	1.719343
1	-10.461037	4.500406	2.322941
1	-9.684557	2.936328	2.080252
1	-11.374859	6.187149	-0.259453
1	-12.302269	4.774398	-0.790831
1	-11.074838	5.482182	-1.856574

cp_{trans}

6	-7.012926	0.149232	1.967166
6	-7.815686	-0.259129	0.949208
6	-7.563218	0.193273	-0.458410
6	-7.101815	1.604003	-0.797207
6	-7.270342	-0.193230	3.343502
7	-7.448812	-0.430407	4.462088
6	-5.827893	0.943479	1.770665
7	-4.862780	1.569001	1.639123
6	-7.103630	-0.931780	-1.344911
8	-7.198164	-2.102393	-1.061627
8	-6.580004	-0.490262	-2.495053
6	-6.257626	-1.505945	-3.449766
6	-8.887455	-1.254526	1.203254
6	-10.203350	-1.049867	0.771933
6	-11.175764	-2.016251	1.016806
6	-10.844473	-3.198598	1.675085
6	-9.532764	-3.411808	2.096300
6	-8.561150	-2.445601	1.865396
1	-6.375733	1.682625	-1.597789
1	-6.969326	2.287988	0.032293
1	-5.859338	-0.977648	-4.313700
1	-7.159070	-2.061584	-3.717483
1	-5.517375	-2.192202	-3.035189
1	-10.465043	-0.116863	0.287746
1	-12.197817	-1.840265	0.695723
1	-11.604278	-3.951721	1.858495
1	-9.262941	-4.333809	2.600518
1	-7.534510	-2.627036	2.167775
6	-10.522888	-0.049626	-4.039896
6	-10.081492	0.780763	-5.070896
6	-9.095997	1.734245	-4.820394
6	-8.559752	1.862399	-3.539866
6	-9.009050	1.044714	-2.503284
6	-9.989596	0.083023	-2.760493
6	-8.507880	1.237553	-1.100585
8	-9.462891	1.721317	-0.211120
14	-10.261955	3.209697	-0.385827
6	-9.002896	4.538346	-0.788421
6	-11.047594	3.467260	1.287212
6	-11.564762	3.092875	-1.727405
1	-11.281942	-0.801061	-4.233090
1	-10.500320	0.680243	-6.067164
1	-8.743998	2.377426	-5.620778
1	-7.784667	2.598729	-3.345145
1	-10.327191	-0.565812	-1.956138
1	-9.501259	5.507767	-0.894970
1	-8.484713	4.329652	-1.729927
1	-8.252034	4.632643	0.002249
1	-11.726073	2.642215	1.525741
1	-11.624623	4.397134	1.311325
1	-10.287803	3.515237	2.072704
1	-12.199935	3.985523	-1.711041
1	-12.206554	2.220519	-1.564954
1	-11.124851	3.004545	-2.725780

$ts2_{trans}$

6	-8.781974	0.542797	1.412939
6	-8.293138	-0.341931	0.417748
6	-7.451699	0.100239	-0.612663
6	-7.513737	1.538217	-1.112137
6	-10.007164	0.337342	2.110485
7	-11.024620	0.234516	2.664251
6	-8.041057	1.694253	1.770623
7	-7.399183	2.629740	2.035583
6	-6.602042	-0.849386	-1.327547
8	-6.320191	-1.976289	-0.976613
8	-6.071717	-0.325596	-2.480355
6	-5.152154	-1.184213	-3.151577
6	-8.802703	-1.738814	0.464236
6	-9.323810	-2.337242	-0.688820
6	-9.859666	-3.618670	-0.635619
6	-9.871786	-4.321062	0.569765
6	-9.354307	-3.731735	1.721607
6	-8.832215	-2.442416	1.671453
1	-6.955884	1.597533	-2.050678
1	-7.125453	2.299149	-0.430760
1	-4.801382	-0.624986	-4.018410
1	-5.646666	-2.108056	-3.460243
1	-4.318046	-1.437052	-2.493772
1	-9.307908	-1.790825	-1.628405
1	-10.267184	-4.071021	-1.534602
1	-10.285723	-5.323875	0.610779
1	-9.361556	-4.273709	2.661755
1	-8.431756	-1.977742	2.567535
6	-11.841142	-0.123063	-2.856829
6	-11.235498	-0.728545	-3.956727
6	-9.879267	-0.518017	-4.221926
6	-9.120607	0.283322	-3.379685
6	-9.734773	0.909193	-2.283117
6	-11.098070	0.709535	-2.026722
6	-8.963380	1.775513	-1.382228
8	-9.638768	2.648584	-0.760819
14	-9.546431	4.266481	-0.027274
6	-7.823720	4.943004	-0.221570
6	-10.244698	4.082021	1.684427
6	-10.727726	5.152700	-1.170944
1	-12.888322	-0.304551	-2.640712
1	-11.817942	-1.373999	-4.606616
1	-9.411672	-0.993607	-5.077472
1	-8.057903	0.410881	-3.560415
1	-11.551129	1.173122	-1.156275
1	-7.866094	6.025192	-0.051446
1	-7.449398	4.790411	-1.239514
1	-7.127819	4.506820	0.497210
1	-10.704695	3.095389	1.811956
1	-11.019436	4.837355	1.848723
1	-9.468493	4.186032	2.444532
1	-10.833182	6.197825	-0.860597
1	-11.719465	4.691729	-1.143024
1	-10.371649	5.143513	-2.205494

prod

6	-9.310252	0.158204	0.606836
6	-8.405233	-0.886241	-0.079486
6	-7.594549	-0.231179	-0.933366
6	-7.877865	1.250733	-0.976751
6	-10.648957	-0.338038	0.958125
7	-11.700949	-0.725431	1.236000
6	-8.640575	0.633020	1.837108
7	-8.063708	0.989546	2.772799
6	-6.460194	-0.824908	-1.686837
8	-6.167638	-1.995125	-1.749169
8	-5.759256	0.141886	-2.309386
6	-4.616367	-0.313732	-3.039017
6	-8.515640	-2.311139	0.274530
6	-8.576478	-3.291747	-0.722152
6	-8.735810	-4.629463	-0.377875
6	-8.831002	-5.002163	0.962215
6	-8.779675	-4.030153	1.959751
6	-8.635882	-2.689191	1.619052
1	-7.757712	1.688466	-1.971322
1	-7.206216	1.784313	-0.291946
1	-4.169231	0.578965	-3.471596
1	-4.921872	-1.013677	-3.819278
1	-3.916039	-0.812499	-2.366310
1	-8.502235	-2.999924	-1.763656
1	-8.786636	-5.381819	-1.158078
1	-8.952774	-6.047375	1.228343
1	-8.859525	-4.312893	3.004141
1	-8.600317	-1.938814	2.404444
6	-12.693176	1.327488	-2.215361
6	-12.394756	0.734314	-3.441361
6	-11.091670	0.323026	-3.706302
6	-10.090519	0.503016	-2.752264
6	-10.379770	1.103190	-1.524180
6	-11.693123	1.510975	-1.266041
6	-9.321264	1.353601	-0.447048
8	-9.605783	2.524996	0.249708
14	-9.339023	4.118056	-0.256769
6	-7.537817	4.564653	0.018389
6	-10.456162	5.120793	0.850713
6	-9.780915	4.322188	-2.067974
1	-13.706739	1.647123	-1.995680
1	-13.173140	0.593528	-4.184460
1	-10.847268	-0.138420	-4.657770
1	-9.082407	0.174439	-2.985003
1	-11.925346	1.979225	-0.314414
1	-7.414497	5.652894	-0.008688
1	-6.882343	4.140326	-0.748666
1	-7.197467	4.211797	0.997472
1	-11.503213	4.840794	0.699951
1	-10.356327	6.191310	0.645447
1	-10.207829	4.949286	1.902291
1	-9.558714	5.347763	-2.383603
1	-10.841876	4.127509	-2.250951
1	-9.206269	3.645514	-2.709558

[sc^{1}dn]$_2$

6	-9.149492	-1.363017	2.248086
6	-8.432104	-1.044853	1.132366
6	-7.726498	0.256758	1.055709
6	-8.331715	1.416430	1.345363
6	-10.037349	-2.492671	2.321550
7	-10.783471	-3.373664	2.412313
6	-9.124928	-0.559171	3.442061
7	-9.130530	0.064199	4.417672
6	-6.302096	0.203935	0.607095
8	-5.639215	-0.805678	0.585759
8	-5.832306	1.408178	0.244561
6	-4.456874	1.430846	-0.147916
6	-8.379639	-1.916624	-0.057713
6	-8.363071	-1.326666	-1.329560
6	-8.348515	-2.119203	-2.470922
6	-8.321983	-3.508452	-2.355869
6	-8.304017	-4.101566	-1.094203
6	-8.333552	-3.312418	0.050292
1	-9.365760	1.462060	1.665230
1	-7.795394	2.354942	1.252570
1	-4.248374	2.463587	-0.421286
1	-4.297756	0.763897	-0.997341
1	-3.823216	1.112894	0.682295
1	-8.395105	-0.244054	-1.421912
1	-8.363099	-1.650649	-3.449858
1	-8.304440	-4.127714	-3.247260
1	-8.259609	-5.181414	-0.998960
1	-8.290816	-3.782193	1.026807
6	-11.709458	-1.082159	-2.840492
6	-12.048478	-1.908926	-1.763195
6	-11.933387	-1.448519	-0.452845
6	-11.453680	-0.160222	-0.221840
6	-11.088416	0.670209	-1.288982
6	-11.233976	0.200358	-2.606374
6	-10.487272	1.978154	-0.936785
8	-10.226099	2.659356	-2.030357
14	-9.357769	4.150746	-1.879746
6	-9.896450	5.040944	-0.333474
6	-9.819741	5.081636	-3.430604
6	-7.549931	3.680261	-1.859339
1	-11.809753	-1.451808	-3.856680
1	-12.404088	-2.918046	-1.948906
1	-12.201302	-2.093860	0.377438
1	-11.348789	0.222047	0.790987
1	-10.952765	0.849842	-3.430006
1	-9.441942	6.036562	-0.292920
1	-9.609429	4.484455	0.561796
1	-10.983988	5.160262	-0.315684
1	-9.568768	4.502333	-4.324126
1	-9.284692	6.035130	-3.484858
1	-10.892616	5.294658	-3.454039
1	-6.917525	4.565204	-1.731091
1	-7.266272	3.195596	-2.799168
1	-7.331491	2.984062	-1.042640

ts1$_{cis}$

6	-9.360525	-1.240939	2.190574
6	-8.584919	-1.063824	1.071111
6	-7.985698	0.233713	0.739081
6	-8.586057	1.430160	1.008866
6	-10.219613	-2.380284	2.363195
7	-10.959700	-3.259091	2.514986
6	-9.446273	-0.280492	3.254342
7	-9.533518	0.474861	4.128820
6	-6.663931	0.201404	0.063237
8	-5.888000	-0.725263	0.108624
8	-6.385165	1.348555	-0.595415
6	-5.082579	1.395762	-1.182055
6	-8.404649	-2.186713	0.120778
6	-8.459330	-1.945485	-1.257556
6	-8.321648	-2.991230	-2.161812
6	-8.100378	-4.288618	-1.700086
6	-8.015831	-4.532793	-0.330375
6	-8.169854	-3.489224	0.576245
1	-9.465315	1.515460	1.629904
1	-8.052967	2.350619	0.801909
1	-5.001502	2.379633	-1.642364
1	-4.977248	0.608041	-1.930722
1	-4.316333	1.263045	-0.415639
1	-8.630949	-0.935460	-1.618827
1	-8.386679	-2.791807	-3.226997
1	-7.984629	-5.105363	-2.405728
1	-7.824885	-5.536536	0.034604
1	-8.080910	-3.681776	1.640513
6	-11.322728	-0.819069	-3.140644
6	-11.889049	-1.676301	-2.190558
6	-11.887927	-1.336966	-0.838496
6	-11.298885	-0.141312	-0.435680
6	-10.709710	0.716686	-1.373928
6	-10.738924	0.374422	-2.737036
6	-10.080143	1.947288	-0.853035
8	-9.757036	2.763959	-1.817420
14	-9.164665	4.368939	-1.503968
6	-9.814627	4.934592	0.148895
6	-9.875276	5.352017	-2.920573
6	-7.303106	4.295217	-1.576365
1	-11.337449	-1.091867	-4.191434
1	-12.330324	-2.615893	-2.508903
1	-12.324733	-2.005630	-0.103552
1	-11.287374	0.143292	0.613115
1	-10.288968	1.048730	-3.459543
1	-9.535406	5.979865	0.319408
1	-9.418185	4.335690	0.972758
1	-10.905991	4.864668	0.180790
1	-9.533481	4.957129	-3.881869
1	-9.565630	6.400027	-2.856244
1	-10.968696	5.320650	-2.909004
1	-6.886116	5.306999	-1.536010
1	-6.975401	3.827092	-2.509987
1	-6.893086	3.714418	-0.745930

cp$_{cis}$

6	-9.053174	-1.390797	1.969571
6	-8.524226	-1.076509	0.749611
6	-8.154574	0.337312	0.430280
6	-8.861696	1.560049	0.983988
6	-9.707775	-2.635629	2.274999
7	-10.279375	-3.598510	2.569998
6	-9.054661	-0.474505	3.077803
7	-9.064141	0.233805	3.994284
6	-6.701876	0.509084	0.054148
8	-5.975491	-0.397513	-0.274727
8	-6.283702	1.777650	0.156679
6	-4.921145	1.996749	-0.217758
6	-8.336317	-2.087599	-0.310696
6	-8.349428	-1.712009	-1.663996
6	-8.179445	-2.660975	-2.663273
6	-7.962211	-3.997952	-2.333161
6	-7.893431	-4.375058	-0.993837
6	-8.073469	-3.428919	0.008376
1	-9.631431	1.440609	1.734528
1	-8.214709	2.416150	1.135034
1	-4.739741	3.057482	-0.052482
1	-4.775879	1.735935	-1.268426
1	-4.258017	1.387543	0.398778
1	-8.477797	-0.672989	-1.942433
1	-8.204589	-2.352390	-3.703288
1	-7.825020	-4.737713	-3.115575
1	-7.687385	-5.405607	-0.724772
1	-7.970448	-3.736586	1.042097
6	-12.121293	-0.063354	-2.459137
6	-13.012480	-0.497505	-1.480267
6	-12.663681	-0.378710	-0.137220
6	-11.433996	0.166069	0.226086
6	-10.524175	0.588412	-0.750172
6	-10.888820	0.473077	-2.097105
6	-9.178374	1.179780	-0.416069
8	-8.602414	1.863457	-1.470717
14	-8.863607	3.516454	-1.756719
6	-10.421455	4.058230	-0.869325
6	-9.034726	3.666241	-3.612156
6	-7.370672	4.474145	-1.157295
1	-12.382265	-0.144705	-3.509667
1	-13.971467	-0.921020	-1.760289
1	-13.350036	-0.705705	0.637079
1	-11.204122	0.258377	1.282311
1	-10.195119	0.806381	-2.862296
1	-10.636645	5.106621	-1.102216
1	-10.324672	3.972847	0.217733
1	-11.282989	3.458350	-1.180038
1	-8.185140	3.196509	-4.117633
1	-9.069609	4.717151	-3.917092
1	-9.951582	3.182736	-3.963324
1	-7.494096	5.543639	-1.358447
1	-6.467496	4.134237	-1.674428
1	-7.212919	4.343176	-0.082346

ts2$_{cis}$

6	-9.441000	-1.173726	1.999875
6	-8.576106	-1.016368	0.898564
6	-8.183199	0.217964	0.356762
6	-8.988826	1.499052	0.515906
6	-10.136250	-2.395550	2.251284
7	-10.746849	-3.361832	2.458600
6	-9.723595	-0.111599	2.900052
7	-9.940634	0.794594	3.597368
6	-7.015109	0.269395	-0.514665
8	-6.103215	-0.526977	-0.575667
8	-7.015296	1.393457	-1.311031
6	-5.893996	1.494305	-2.184838
6	-8.170180	-2.280027	0.216732
6	-8.459504	-2.451276	-1.140722
6	-8.140565	-3.644226	-1.781012
6	-7.520164	-4.671997	-1.071419
6	-7.232120	-4.506061	0.282262
6	-7.565833	-3.318781	0.926924
1	-9.726434	1.430597	1.319591
1	-8.362581	2.365924	0.734615
1	-6.123895	2.301968	-2.880991
1	-5.745069	0.558135	-2.725048
1	-4.986289	1.723695	1.619756
1	-8.941925	-1.644669	-1.689381
1	-8.375631	-3.772526	-2.833195
1	-7.265688	-5.601439	-1.571344
1	-6.751244	-5.304228	0.838493
1	-7.349034	-3.186803	1.982901
6	-11.537073	-0.443298	-3.249813
6	-12.240366	-1.298138	-2.396729
6	-12.140294	-1.154836	-1.012708
6	-11.317951	-0.173222	-0.474038
6	-10.587991	0.672548	-1.327964
6	-10.722012	0.547903	-2.721317
6	-9.691154	1.686603	-0.784663
8	-9.484175	2.712401	-1.527000
14	-8.911329	4.330873	-1.134229
6	-9.969741	4.866442	0.305362
6	-9.324587	5.224029	-2.712956
6	-7.088142	4.401781	-0.762062
1	-11.620253	-0.558396	-4.325340
1	-12.870438	-2.077592	-2.813101
1	-12.689946	-1.813569	-0.349017
1	-11.254225	-0.059930	0.601804
1	-10.152836	1.205897	-3.369647
1	-9.738576	4.320948	1.225120
1	-11.032263	4.729218	0.082434
1	-9.806679	5.931006	0.504196
1	-8.781894	4.790038	-3.558214
1	-9.044403	6.279564	-2.639900
1	-10.395221	5.169389	-2.928521
1	-6.857270	5.432401	-0.466804
1	-6.490862	4.166295	-1.645572
1	-6.775095	3.736647	0.044823

int$_1$

6	-9.066578	-0.797504	2.268742
6	-8.500834	-1.013999	0.990324
6	-8.440795	-0.004313	0.020258
6	-9.336336	1.211953	0.134992
6	-9.500946	-1.863817	3.111493
7	-9.877415	-2.713492	3.808481
6	-9.131571	0.512161	2.820191
7	-9.136881	1.611026	3.205985
6	-7.484554	-0.025721	-1.066535
8	-6.616028	-0.829248	-1.332174
8	-7.649185	1.099554	-1.865678
6	-6.810214	1.157625	-3.012559
6	-8.020148	-2.389288	0.682186
6	-8.415478	-3.026633	-0.498759
6	-8.006163	-4.326522	-0.769778
6	-7.185820	-5.002008	0.134438
6	-6.783694	-4.372661	1.310306
6	-7.205743	-3.075119	1.586932
1	-10.230753	0.931645	0.713640
1	-8.893057	2.043275	0.687470
1	-7.092939	2.067344	-3.546074
1	-6.962868	0.280994	-3.646091
1	-5.759907	1.198697	-2.714097
1	-9.038423	-2.489442	-1.209505
1	-8.321487	-4.813021	-1.687578
1	-6.862583	-6.016460	-0.077800
1	-6.146162	-4.893346	2.017444
1	-6.899393	-2.584532	2.505925
6	-11.622610	0.530067	-4.250753
6	-12.073119	-0.725342	-3.834724
6	-11.805640	-1.183197	-2.544462
6	-11.074522	-0.393806	-1.664697
6	-10.621289	0.868932	-2.079357
6	-10.904183	1.330535	-3.374058
6	-9.864088	1.717727	-1.162303
8	-9.779142	2.952407	-1.464632
14	-8.966913	4.307644	-0.639733
6	-9.861729	4.572374	0.970400
6	-9.295828	5.659826	-1.876116
6	-7.155348	3.909018	-0.493584
1	-11.833577	0.878883	-5.255849
1	-12.637112	-1.349288	-4.520994
1	-12.162465	-2.155790	-2.223281
1	-10.860104	-0.754839	-0.663782
1	-10.545081	2.307349	-3.680342
1	-9.604932	3.840446	1.743305
1	-10.945673	4.549783	0.818201
1	-9.607287	5.566676	1.354380
1	-8.859450	5.417968	-2.849822
1	-8.854719	6.601652	-1.534948
1	-10.369689	5.819555	-2.009295
1	-6.640444	4.790106	-0.093962
1	-6.726228	3.679438	-1.472820
1	-6.950881	3.066733	0.172054

$int_{1'}$

6	-8.914208	-0.620794	1.781032
6	-8.201146	-1.157410	0.669584
6	-7.761184	-0.380201	-0.398614
6	-7.607442	1.109732	-0.268243
6	-9.046426	-1.307805	3.023183
7	-9.168394	-1.840674	4.048349
6	-9.603648	0.608398	1.678386
7	-10.176555	1.613210	1.513818
6	-7.529796	-0.954758	-1.720798
8	-7.768113	-2.081518	-2.106438
8	-7.053888	-0.005639	-2.593288
6	-6.943727	-0.442754	-3.943485
6	-7.920222	-2.619456	0.707004
6	-8.940075	-3.533669	0.982214
6	-8.668087	-4.898122	1.025259
6	-7.370064	-5.357927	0.813763
6	-6.344200	-4.448488	0.555688
6	-6.619291	-3.087323	0.498401
1	-7.402196	1.417926	0.761491
1	-6.744290	1.440358	-0.867910
1	-6.552193	0.407787	-4.501114
1	-7.924712	-0.735675	-4.327946
1	-6.267758	-1.296828	-4.019920
1	-9.949074	-3.172024	1.158616
1	-9.468911	-5.600660	1.232640
1	-7.156049	-6.421413	0.856439
1	-5.329852	-4.801761	0.398951
1	-5.827146	-2.372847	0.293306
6	-10.713473	1.976850	-3.925546
6	-11.447452	0.796729	-3.799689
6	-11.310020	-0.001531	-2.664258
6	-10.426942	0.358930	-1.652218
6	-9.701798	1.553510	-1.769330
6	-9.853637	2.363782	-2.905594
6	-8.706960	1.986429	-0.780714
8	-8.637800	3.211289	-0.455614
14	-9.834778	4.505477	-0.192104
6	-11.562045	3.891602	-0.499590
6	-9.279069	5.827964	-1.386724
6	-9.502639	4.956730	1.576367
1	-10.810357	2.590885	-4.814625
1	-12.126294	0.496365	-4.591542
1	-11.884298	-0.916264	-2.567515
1	-10.328920	-0.258992	-0.767825
1	-9.262296	3.270188	-3.002574
1	-12.252187	4.619397	-0.056589
1	-11.719716	2.933416	0.002958
1	-11.802250	3.793073	-1.560669
1	-8.200071	5.993921	-1.315259
1	-9.776761	6.771728	-1.139509
1	-9.527925	5.586733	-2.423975
1	-10.141304	5.793213	1.878630
1	-8.459774	5.254243	1.717514
1	-9.718590	4.099667	2.219616

$ts3_{cis}$

6	-8.526380	-0.171608	1.484314
6	-7.887230	-0.818457	0.383706
6	-7.281386	-0.165882	-0.680626
6	-7.321850	1.333054	-0.829178
6	-9.486807	-0.852892	2.290651
7	-10.301275	-1.365144	2.943518
6	-8.322421	1.179415	1.856975
7	-8.181877	2.294203	2.168973
6	-6.470449	-0.903617	-1.656746
8	-6.019739	-2.024330	-1.552645
8	-6.222788	-0.158434	-2.781080
6	-5.399049	-0.800485	-3.750444
6	-8.050154	-2.304340	0.365485
6	-8.690679	-2.932520	-0.706735
6	-8.890273	-4.308599	-0.698483
6	-8.443898	-5.072320	0.379634
6	-7.806806	-4.451158	1.451634
6	-7.621684	-3.071580	1.450169
1	-6.786032	1.886247	-0.051523
1	-6.828646	1.589766	-1.781778
1	-5.280151	-0.082592	-4.561287
1	-5.876120	-1.714034	-4.111933
1	-4.429890	-1.058928	-3.318305
1	-9.017228	-2.337162	-1.556266
1	-9.389180	-4.787083	-1.535604
1	-8.596119	-6.147137	0.385241
1	-7.460912	-5.039366	2.295524
1	-7.138537	-2.582099	2.290732
6	-11.411587	1.712795	-3.472634
6	-12.035556	0.524730	-3.089808
6	-11.548091	-0.207213	-2.008040
6	-10.426058	0.227131	-1.308571
6	-9.812605	1.432155	-1.676351
6	-10.314365	2.176385	-2.757816
6	-8.640985	2.004535	-0.992935
8	-8.671866	3.230337	-0.667362
14	-9.968766	4.308511	-0.067207
6	-11.284980	3.210935	0.652997
6	-10.542829	5.314671	-1.534163
6	-9.043727	5.350997	1.152700
1	-11.779177	2.276318	-4.323731
1	-12.903018	0.169352	-3.636777
1	-12.038527	-1.124591	-1.699916
1	-10.077024	-0.326315	-0.446925
1	-9.815098	3.092691	-3.058469
1	-11.973179	3.830664	1.238550
1	-10.846816	2.472569	1.332279
1	-11.865264	2.692983	-0.115807
1	-9.701049	5.610934	-2.168486
1	-11.010214	6.234211	-1.165366
1	-11.278413	4.785166	-2.144795
1	-9.728225	6.059460	1.631087
1	-8.256323	5.923833	0.653613
1	-8.591698	4.716836	1.918998

References

(1) Frisch, M. J.; Trucks, G. W.; Schlegel, H. B.; Scuseria, G. E.; Robb, M. A.; Cheeseman, J. R.; Scalmani, G.; Barone, V.; Petersson, G. A.; Nakatsuji, H.; Li, X.; Caricato, M.; Marenich, A. V.; Bloino, J.; Janesko, B. G.; Gomperts, R.; Mennucci, B.; Hratchian, H. P.; Ortiz, J. V.; Izmaylov, A. F.; Sonnenberg, J. L.; Williams-Young, D.; Ding, F.; Lipparini, F.; Egidi, F.; Goings, J.; Peng, B.; Petrone, A.; Henderson, T.; Ranasinghe, D.; Zakrzewski, V. G.; Gao, J.; Rega, N.; Zheng, G.; Liang, W.; Hada, M.; Ehara, M.; Toyota, K.; Fukuda, R.; Hasegawa, J.; Ishida, M.; Nakajima, T.; Honda, Y.; Kitao, O.; Nakai, H.; Vreven, T.; Throssell, K.; J. A. Montgomery, J.; Peralta, J. E.; Ogliaro, F.; Bearpark, M. J.; Heyd, J. J.; Brothers, E. N.; Kudin, K. N.; Staroverov, V. N.; Keith, T. A.; Kobayashi, R.; Normand, J.; Raghavachari, K.; Rendell, A. P.; Burant, J. C.; Iyengar, S. S.; Tomasi, J.; Cossi, M.; Millam, J. M.; Klene, M.; Adamo, C.; Cammi, R.; Ochterski, J. W.; Martin, R. L.; Morokuma, K.; Farkas, O.; Foresman, J. B.; Fox, D. J. Gaussian 16, Revision C.01, Gaussian, Inc.: Wallingford CT, 2019.

(2) (a) Zhao, Y.; Truhlar, D., The M06 suite of density functionals for main group thermochemistry, thermochemical kinetics, noncovalent interactions, excited states, and transition elements: two new functionals and systematic testing of four M06-class functionals and 12 other functionals. Theor. Chem. Acc. 2008, 120, 215-241; (b) Zhao, Y.; Truhlar, D. G., Density functionals with broad applicability in chemistry. *Acc. Chem. Res.* **2008**, *41*, 157-67.

(3) (a) Ditchfield, R.; Hehre, W. J.; Pople, J. A., Self-Consistent Molecular-Orbital Methods. IX. An Extended Gaussian-Type Basis for Molecular-Orbital Studies of Organic Molecules. *J. Chem. Phys.* **1971,** *54*, 724-728; (b) Hehre, W. J.; Ditchfield, R.; Pople, J. A., Self—Consistent Molecular Orbital Methods. XII. Further Extensions of Gaussian—Type Basis Sets for Use in Molecular Orbital Studies of Organic Molecules. *J. Chem. Phys.* **1972,** *56*, 2257-2261; (c) Hariharan, P. C.; Pople, J. A., Accuracy of AH_n Equilibrium Geometries by Single Determinant Molecular Orbital Theory. *Mol. Phys.* **1974,** *27*, 209-214; (d) Gordon, M. S., The Isomers of Silacyclopropane. *Chem. Phys. Lett.* **1980,** *76*, 163-168; (e) Hariharan, P. C.; Pople, J. A., The Influence of Polarization Functions on Molecular Orbital Hydrogenation Energies. *Theor. Chim. Acta* **1973,** *28*, 213-222.

(4) (a) Peng, C.; Ayala, P. Y.; Schlegel, H. B.; Frisch, M. J., Using Redundant Internal Coordinates to Optimize Equilibrium Geometries and Transition States. *J. Comput. Chem.* **1996,** *17*, 49-56; (b) Peng, C.; Bernhard Schlegel, H., Combining Synchronous Transit and Quasi-Newton Methods to Find Transition States. *Isr. J. Chem.* **1993,** *33*, 449-454.

(5) (a) Carpenter, J. E.; Weinhold, F., Analysis of the geometry of the hydroxymethyl radical by the "different hybrids for different spins" natural bond orbital procedure. *J. Mol. Struct. Theochem* **1988**, *169*, 41-62; (b) Carpenter, J. E. Ph.D. Thesis. University of Wisconsin Madison WI, 1987; (c) Foster, J. P.; Weinhold, F., Natural hybrid orbitals. *J. Am. Chem. Soc.* **1980**, *102*, 7211-7218; (d) Reed, A. E.; Weinhold, F., Natural Bond Orbital Analysis of Near-Hartree–Fock Water Dimer. *J. Chem. Phys.* **1983**, *78*, 4066-4073; (e) Reed, A. E.; Weinhold, F., Natural localized molecular orbitals. *J. Chem. Phys.* **1985**, *83*, 1736-1740; (f) Reed, A. E.; Weinstock, R. B.; Weinhold, F., Natural population analysis. *J. Chem. Phys.* **1985**, *83*, 735-746; (g) Reed, A. E.; Curtiss, L. A.; Weinhold, F., Intermolecular interactions from a natural bond orbital, donor-acceptor viewpoint. *Chem. Rev.* **1988**, *88*,

899-926; (h) Weinhold, F.; Carpenter, J. E., In *The Structure of Small Molecules and Ions*, Naaman, R.; Vager, Z., Eds. Plenum: New York, **1988**; pp 227-236.

(6) (a) Wiberg, K. B., Application of the Pople-Santry-Segal CNDO Method to the Cyclopropylcarbinyl and Cyclobutyl Cation and to Bicyclobutane. *Tetrahedron* **1968,** *24*, 1083-1096; (b) Yao, M.-L.; Reddy, M. S.; Zeng, W.; Hall, K.; Walfish, I.; Kabalka, G. W., Identification of a Boron-Containing Intermediate in the Boron Tribromide Mediated Aryl Propargyl Ether Cleavage Reaction. *J. Org. Chem.* **2009,** *74*, 1385-1387.

(7) (a) Cancès, E.; Mennucci, B.; Tomasi, J., A new integral equation formalism for the polarizable continuum model: Theoretical background and applications to isotropic and anisotropic dielectrics. *J. Chem. Phys.* **1997,** *107*, 3032-3041; (b) Mennucci, B.; Tomasi, J., Continuum solvation models: A new approach to the problem of solute's charge distribution and cavity boundaries. *J. Chem. Phys.* **1997,** *106*, 5151-5158; (c) Cossi, M.; Barone, V.; Mennucci, B.; Tomasi, J., Ab initio study of ionic solutions by a polarizable continuum dielectric model. *Chem. Phys. Lett.* **1998,** *286*, 253-260.

(8) Marenich, A. V.; Cramer, C. J.; Truhlar, D. G., Universal Solvation Model Based on Solute Electron Density and on a Continuum Model of the Solvent Defined by the Bulk Dielectric Constant and Atomic Surface Tensions. *J. Phys. Chem. B* **2009,** *113*, 6378-6396.
